# Interventions to treat fear of childbirth in pregnancy: a systematic review and meta-analysis

**DOI:** 10.1017/S0033291721002324

**Published:** 2021-09

**Authors:** Rebecca Webb, Rod Bond, Borja Romero-Gonzalez, Rachel Mycroft, Susan Ayers

**Affiliations:** 1 Centre for Maternal and Child Health Research, School of Health Sciences, City, University of London, London, UK; 2 School of Psychology, University of Sussex, Brighton, UK; 3 Psychology Department, Faculty of Education, Campus Duques de Soria. University of Valladolid, Spain; 4 Community Perinatal Psychology, South London and Maudsley NHS Foundation Trust, UK.

**Keywords:** Fear of childbirth, interventions, systematic review, tokophobia

## Abstract

**Background:**

Between 5% and 14% of women suffer from fear of childbirth (FOC) which is associated with difficulties during birth and in postnatal psychological adjustment. Therefore, effective interventions are needed to improve outcomes for women. A systematic review and meta-analysis was used to identify effective interventions for treating women with FOC.

**Methods:**

Literature searches were undertaken on online databases. Hand searches of reference lists were also carried out. Studies were included in the review if they recruited women with FOC and aimed to reduce FOC and/or improve birth outcomes. Data were synthesised qualitatively and quantitatively using meta-analysis. The literature searches provided a total of 4474 citations.

**Results:**

After removing duplicates and screening through abstracts, titles and full texts, 66 papers from 48 studies were identified for inclusion in the review. Methodological quality was mixed with 30 out of 48 studies having a medium risk of bias. Interventions were categorised into six broad groups: cognitive behavioural therapy, other talking therapies, antenatal education, enhanced midwifery care, alternative interventions and interventions during labour. Results from the meta-analysis showed that most interventions reduced FOC, regardless of the approach (mean effect size = −1.27; *z* = −4.53, *p* < 0.0001) and that other talking therapies may reduce caesarean section rates (OR 0.48, 95% CI 0.48–0.90).

**Conclusions:**

Poor methodological quality of studies limits conclusions that can be drawn; however, evidence suggests that most interventions investigated reduce FOC. Future high-quality randomised controlled trials are needed so that clear conclusions can be made.

## Introduction

Research suggests approximately 14% of women are affected by extreme fear of childbirth (FOC), also known as tokophobia (Nilsson et al., 2018; O'Connell, Leahy-Warren, Khashan, Kenny, & O'Neill, 2017). The causes of FOC are thought to be linked to obstetric (Fairbrother, Thordarson, & Stoll, 2018; Haines, Pallant, Karlström, & Hildingsson, 2011; Sydsjö et al., 2013; Sydsjö et al., 2014), psychological (Dencker et al., 2019; Hall, Stoll, Hutton, & Brown, 2012; Jokić-Begić, Žigić, & Nakić Radoš, 2014; Lukasse, Vangen, Ãian, & Schei, 2011) and socio-demographic factors (Haines et al., 2011; O'Connell, Leahy-Warren, Kenny, O'Neill, & Khashan, 2019; Ryding et al., 2015). FOC can impact on women's birth choices and outcomes. For example, those with FOC are more likely to opt for an elective caesarean section (CS) (Eide, Morken, & Bærøe, 2019; Ryding et al., 2015; Sydsjö, Sydsjö, Gunnervik, Bladh, & Josefsson, 2012), choose an epidural, have longer labours (Dencker et al., 2019; Logtenberg et al., 2018; Reck et al., 2013) or have an emergency CS (Sydsjö et al., 2012).

FOC has also been found to be related to postnatal psychological adjustment. For example, FOC is associated with postnatal post-traumatic stress disorder (Capik & Durmaz, 2018; Söderquist, Wijma, & Wijma, 2004; Wijma, Ryding, & Wijma, 2002; Wijma, Söderquist, & Wijma, 1997), depression and anxiety (Räisänen et al., 2013; Rouhe, Salmela-Aro, Gissler, Halmesmäki, & Saisto, 2011) as well as antenatal depression and anxiety (Andersson, Sundström-Poromaa, Wulff, Åström, & Bixo, 2004). The long-term impact of FOC on mother–infant outcomes has yet to be investigated; however, research has evaluated the impact of the psychological conditions associated with FOC such as anxiety and depression (Stein et al., 2014). Antenatal mood disorders have been associated with altered patterns of foetal behaviour and heart rate responses (Kinsella & Monk, 2009), more fearful or anxious behaviour in the infant, and increased risk of poor development and adverse child outcomes (Talge, Neal, & Glover, 2007). Furthermore, antenatal depressive symptoms have been linked to poorer maternal responsiveness 12 months postnatally (Pearson et al., 2012).

The development of FOC has still yet to be established in the literature; however, a systematic review of 89 studies found that a variety of factors may contribute to the development (Rondung, Thomtén, & Sundin, 2016). In a theory of fear acquisition, Rachman (1977) proposed that fears are developed through three pathways (conditioning, vicarious experiences and transmission of information), and the literature appears to support this model (Rondung et al., 2016). For example, in terms of conditioning, research suggests that negative birth experiences cause future FOC (Lukasse et al., 2011; Lukasse, Schei, & Ryding, 2014; Nilsson, Lundgren, Karlström, & Hildingsson, 2012). Research also suggests that vicarious experiences such as viewing a live birth is associated with a reduction in fear (Stoll & Hall, 2013) and research also suggests that the transmission of information such as negative childbirth stories (Melender, 2002; Tsui et al., 2007) and public discourses of birth (Fenwick, Hauck, Downie, & Butt, 2005; Melender, 2002) also contribute to the development of FOC.

The systematic review also suggested that cognitive aspects may play an important role in FOC (Rondung et al., 2016). For example, women with childbirth fear more commonly report having childbirth-related thoughts compared with women with no fear (Hildingsson, Thomas, Karlström, Olofsson, & Nystedt, 2010). Furthermore, FOC is negatively correlated with childbirth self-efficacy (Beebe, Lee, Carrieri-Kohlman, & Humphreys, 2007) and a woman's appraisal of her ability to cope with stressful situations (Ryding, Wijma, Wijma, & Rydhström, 1998; Söderquist et al., 2004). The review found that behavioural aspects are also important (Rondung et al., 2016) with FOC being associated with avoidance of pregnancy (Tsui et al., 2007), or avoidance of vaginal delivery (Dehghani, Sharpe, & Khatibi, 2014; D'Cruz & Lee, 2014; Matinnia et al., 2015; Nieminen, Stephansson, & Ryding, 2009). Physiological aspects may also be associated with FOC, such as sleep disturbances, tachycardia, tenseness, restlessness and nervousness (Rondung et al., 2016). This complex conceptualisation of the potential causes of FOC suggests that interventions for FOC need to focus on strategies to improve psychological, cognitive, behavioural and physiological aspects of the fear.

To prevent poorer outcomes related to FOC, early detection and evidence-based interventions are key, and should therefore be available to women. Reviews of the evidence suggest that interventions with an educational component may reduce FOC (Moghaddam Hosseini, Nazarzadeh, & Jahanfar, 2018; Stoll, Swift, Fairbrother, Nethery, & Janssen, 2018; Striebich, Mattern, & Ayerle, 2018). However, previous reviews used narrow inclusion criteria such as requiring a minimum score on a FOC measure (Striebich et al., 2018); requiring FOC to be measured twice (Stoll et al., 2018); or only including clinical trials (Moghaddam Hosseini et al., 2018). This means the reviews include very few studies (range = 7–15) and may exclude studies that could provide further insight into effective FOC treatments. Further, the previous reviews did not evaluate evidence quantitatively using meta-analysis. This may have led to a bias in conclusions where interventions that have been more widely examined are more likely to be evaluated as promising. Therefore, this study aimed to conduct a systematic review and meta-analysis to identify the effectiveness of FOC interventions for reducing FOC or rates of caesarean by request during the perinatal period.

## Methods

### Search strategies

Literature searches and study selection were conducted according to the Preferred Reporting Items for Systematic Reviews and Meta-analyses (PRISMA) guidelines (Moher et al., 2015) (online Supplementary Table S1). The protocol was registered with PROSPERO (CRD42018093095). Online databases were used to identify papers. Searches were carried out in Cochrane Library (1996–present), EMBASE (1947–present), Healthcare Management Information Consortium (HMIC; 1983–present), Medline (1946–present); PsychInfo (1967–present), PsychARTICLES (1806–present), PubMed (1996–present), SCOPUS (2004–present) and Web of Science (1997–present). Pre-planned searches were carried out using search terms combined with Boolean operators ‘OR’ and ‘AND’ (e.g. pregnancy OR perinatal OR postnat* AND fear of childbirth OR tokophobia AND Caesarean OR abdom* deliver* AND counselling OR intervention). Full search syntax can be found in the supporting information. Searches were conducted up to and including June 2019. Two searches were carried out: (1) desire for an elective CS and interventions; (2) FOC and interventions (see Appendix S1). Desire for an elective CS was used as a proxy measure for FOC. Reference lists of all identified papers were also searched.

### Inclusion and exclusion criteria

The following inclusion criteria were applied: Participants – women in the perinatal period (the UK definition of perinatal period was used: pregnancy – 1 year after birth; NHS England, 2019) with FOC or tokophobia; Intervention – any intervention that was for women with FOC; Outcome – a measure of FOC or birth outcomes. Studies were excluded if they were conference abstracts, theses, non-English publications or non-empirical papers.

### Study selection and data extraction

The results from both searches were imported into Eppi-Reviewer 4. All duplicate papers were removed and studies screened for eligibility based on their title and abstract by one reviewer (RW). A proportion (10% *n* = 284) were double screened by BRG. Reviewers agreed on inclusion/exclusion of studies 99% (*n* = 282/284) of the time. Studies that were not eligible were excluded. Full texts of studies that appeared to meet criteria or where it was unclear were then reviewed by one reviewer (RW) to determine whether they should be included. A proportion (10%, *n* = 9) were double screened by BRG. Reviewers agreed on inclusion/exclusion of studies 100% of the time. A decision to only double screen a proportion of titles and abstracts and papers was made based on the high level of agreement on screening suggesting that the inclusion and exclusion criteria were clear and that screening was accurate, the similar approach to double coding used in other reviews (Furuta, Sandall, & Bick, 2012; Lucas, Olander, Ayers, & Salmon, 2019; Sambrook Smith, Lawrence, Sadler, & Easter, 2019).

A data extraction sheet was designed and used to extract relevant information from the full texts. This included: (1) measure used; (2) language of the measure; (3) country; (4) number of participants; (5) participant group; (6) participant demographics; (7) design of the study; (8) norming data for the measure; (9) cut-off scale of the measure; (10) how the measure was administered. The primary outcome measure was reduction in FOC. Other outcome measures were self-efficacy, obstetric outcomes and childbirth experience. Data extraction for all studies was completed by RW. BRG completed data extraction for a proportion of studies (10%, *n* = 7). Agreement was high (88%). RW and BRG extracted effect sizes and statistics for the meta-analysis in duplicate.

### Quality assessment

The Cochrane Risk of Bias Tool (Higgins et al., 2011) was used to assess quality. Due to the nature of psychological intervention studies, both participants and personnel were aware of the intervention; therefore, as all studies would score negatively on the performance bias items, this was not assessed. The tool was adapted so that each bias criterion could be answered as ‘yes’, ‘no’ or ‘not applicable’ (n/a). Items that were scored yes were assigned a score of 1, items that were scored no were assigned a score of 0. These were then averaged (excluding the answers scored n/a) and multiplied by 100. Studies that scored between 0 and 33 were labelled as having a high risk of bias, those scoring between 34 and 66 were labelled as having a medium risk of bias, studies that scored between 67 and 100 were labelled as having a low risk of bias.

### Data analysis

Studies were synthesised narratively then meta-analysis used to determine whether FOC interventions were effective for two types of outcome: (1) FOC in late pregnancy; and (2) CS birth. Potential moderators of the effectiveness of FOC interventions were examined, i.e. risk of bias, country, study design, sample (only women with FOC *v.* all women; nulliparous *v.* mixed parity), intensity of the intervention and type of intervention. Studies were excluded from the meta-analysis if they did not report effect sizes for relevant outcomes (*n* = 31), were based on samples with men only (*n* = 1), did not have a control group (*n* = 5), or where the FOC intervention was provided intrapartum (*n* = 1). Effect sizes were calculated as Cohen's *d* using the difference between the pre- and post-test means to control for any pretest group differences, and the posttest standard deviations (Lipsey & Wilson, 2001). For Haapio, Kaunonen, Arffman, and Åstedt-Kurki (2017), Cohen's *d* was derived from the odds ratio (Borenstein, Hedges, Higgins, & Rothstein, 2009). Meta-analysis was carried out using R where the computation of a *Q* statistic was made. To see if publication bias was of any concern in this analysis, a funnel plot and trim and fill analysis (Duval & Tweedie, 2000) were carried out using R.

## Results

### Study characteristics

**Fig. 1.**
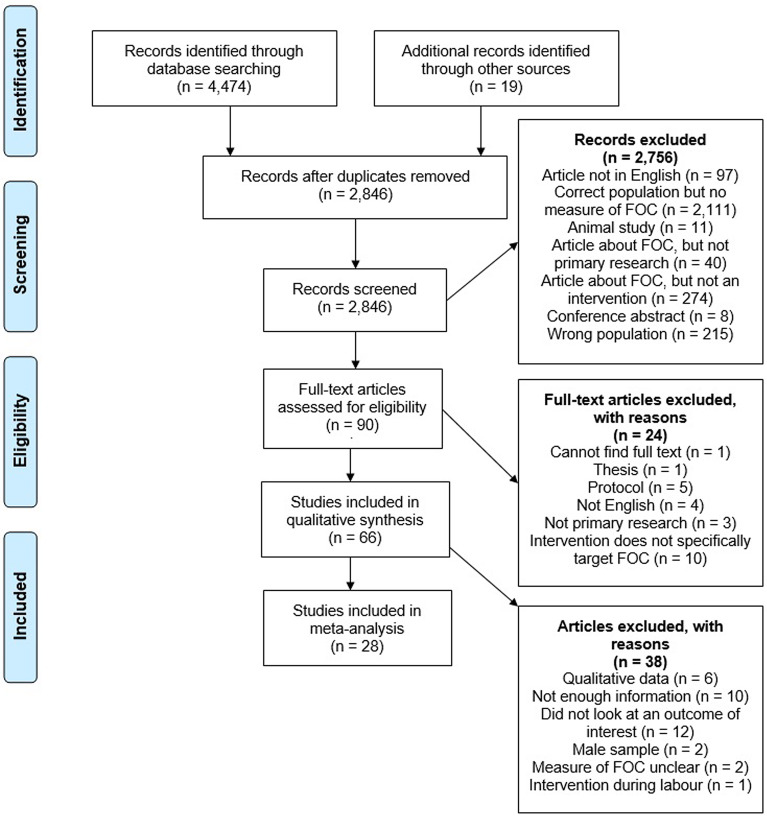
PRISMA Flow Diagram.

Searches identified a total of 4474 citations. Hand searches of reference lists of key papers identified a further 19 papers. After removing duplicates and screening through abstracts, titles and full texts, 66 papers from 48 studies remained for inclusion in the review (Fig. 1). Participants within the studies varied. Only three studies reported ethnicity, all recruited white women (range: 17–71%) and two studies reported recruiting Black women (range: 25–50%). University education was reported by 19 studies, 12 of these reported more than half of participants having completed university. Of the 18 studies that reported marital status, between 57% and 100% of the participants were married or cohabiting. Most papers (*n* = 27) used the Wijma Delivery Expectance Questionnaire (WDEQ-A) to measure FOC. Scores of ⩽37 are indicative of mild fear, scores of 38–65 moderate fear, scores of 66–84 severe fear, and scores of ⩾85 clinical fear (Wijma, Wijma, & Zar, 1998). Average WDEQ-A scores ranged from 29.7 to 130.

One paper described their outcome variable as ‘tokophobia’ (Pour-Edalati, Moghadam, Shahesmaeili, & Salehi-Nejad, 2019) and one paper used the terms FOC and tokophobia interchangeably (Bulez, Ceber Turfan, & Sogukpinar, 2019). The remaining studies used FOC as their outcome variable. The majority of papers examined FOC interventions with women (see online Supplementary Table S2). The exceptions were two studies with fathers (Bergström, Rudman, Waldenström, & Kieler, 2013; Ryding et al., 2018) and one with couples (Ahmadi et al., 2018). Studies were mostly conducted in non-English speaking countries: Iran (*n* = 8), Sweden (*n* = 9), Turkey (*n* = 9). Sample sizes ranged from 10 to 1887. Three studies had a high risk of bias, 30 had a medium risk of bias and 15 had a low risk of bias (online Supplementary Table S3).

### Interventions identified

Interventions were grouped into six broad categories:

- Cognitive/cognitive behavioural (11 papers from six studies): these were interventions which used strategies to change cognitions (e.g. psychoeducation, thought restructuring, problem solving) and behaviours (e.g. exposure, relaxation).
- Other talking therapies (16 papers from 12 studies): interventions that used therapeutic conversation (e.g. counselling, haptotherapy, psychotherapy).
- Antenatal education (18 papers from 13 studies): these were interventions where the main focus was education about pregnancy and birth.
- Enhanced midwifery care (six papers from three studies) comprised continuity of carer or a midwife-led visit to delivery suite.
- Alternative interventions (13 papers from 12 studies): interventions involving specific therapies such as hypnobirthing, stand-alone relaxation, Pilates, art therapy.
- Interventions during labour (two papers from two studies) used intrapartum music or emotional freedom technique (EFT)

#### Cognitive/cognitive behavioural

Six studies examined cognitive behavioural therapy (CBT) delivered online (*n* = 2; Baylis, Ekdahl, Haines, & Rubertsson, 2019; Hildingsson & Rubertsson, 2019; Larsson et al., 2017; Larsson, Hildingsson, Ternström, Rubertsson, & Karlström, 2019; Nieminen et al., 2015; Nieminen, Andersson, Wijma, Ryding, & Wijma, 2016; Rondung et al., 2018) or face-to-face (*n* = 4; Kordi, Bakhshi, Masoudi, & Esmaily, 2017; Saisto, Salmela-Aro, & Nurmi, 2001; Sydsjö et al., 2015; Uçar & Golbasi, 2019). Studies either had no control group (*n* = 1; Nieminen et al., 2015, 2016) or compared the intervention to other talking therapies (*n* = 2; Baylis et al., 2019; Hildingsson & Rubertsson, 2019; Larsson et al., 2017, 2019; Rondung et al., 2018; Saisto et al., 2001) or standard medical care (SMC; *n* = 3; Kordi et al., 2017; Sydsjö et al., 2015; Uçar & Golbasi, 2019). Two studies had a low risk of bias and the rest medium.

Three studies, including online CBT, found a reduction in FOC symptoms (Kordi et al., 2017; Nieminen et al., 2016; Uçar & Golbasi, 2019). However, there was no impact of CBT on birth preference, birth mode (Larsson et al., 2017) or birth experience (Hildingsson & Rubertsson, 2019), in fact one study found CBT was associated with an increase in negative birth outcomes (Sydsjö et al., 2015).

#### Other talking therapies

Twelve studies examined other talking therapies (excluding CBT) delivered face-to-face (*n* = 11; Ahmadi et al., 2018; Andaroon, Kordi, Kimiaei, & Esmaeily, 2017; Halvorsen, Nerum, Sørlie, & Øian, 2010; Henriksen, Borgen, Risløkken, & Lukasse, 2018; Klabbers, Paarlberg, & Vingerhoets, 2018; Klabbers, Wijma, Paarlberg, Emons, & Vingerhoets, 2019; Larsson, Karlström, Rubertsson, & Hildingsson, 2015; Nerum, Halvorsen, Sørlie, & Øian, 2006; Ryding, Persson, Onell, & Kvist, 2003; Sjogren, 1998; Soltani, Eskandari, Khodakarami, Parsa, & Roshanaei, 2017; Sydsjö et al., 2012) or over the telephone (*n* = 1; Fenwick et al., 2015; Toohill et al., 2014; Toohill, Callander, Gamble, Creedy, & Fenwick, 2017; Turkstra et al., 2017). These approaches are a very diverse group, drawing on different theoretical frameworks including generic counselling, haptotherapy and psychotherapy. Studies either had no control group (*n* = 2) or compared other talking therapies to SMC (*n* = 7) or no intervention (*n* = 3). Two studies had a low risk of bias, one had high risk and the remaining medium risk.

Three studies, including other talking therapies carried out over the phone, found a reduction in FOC (Andaroon et al., 2017; Soltani et al., 2017; Toohill et al., 2014). However, two studies found no change in women's FOC after receiving other talking therapy (Larsson et al., 2015; Ryding et al., 2003). All three studies that looked at the impact of other talking therapies on birth preference found a reduction in desire for a CS (Fenwick et al., 2015; Halvorsen et al., 2010; Nerum et al., 2006). Three studies found other talking therapy was associated with a lower risk of CS (Ahmadi et al., 2018; Fenwick et al., 2015; Toohill et al., 2017); however, two studies found an increased risk of CS (Henriksen et al., 2018; Sydsjö et al., 2012).

#### Antenatal education

Thirteen studies examined antenatal education classes. All but two (Khedr & Eldeen, 2017; Kulkarni, Wright, & Kingdom, 2014) interventions were delivered face-to-face. Studies either had no control group (*n* = 2; Khedr & Eldeen, 2017; Kulkarni et al., 2014) or compared antenatal education to SMC (*n* = 10) or no intervention (*n* = 1; Taheri, Mazaheri, Khorsandi, Hassanzadeh, & Amiri, 2014). Five studies were RCTs with low risk of bias (Bergström et al., 2013; Haapio et al., 2017; Masoumi et al., 2016; Ozdemir, Cilingir, Ilhan, Yildiz, & Ohanoglu, 2018; Rouhe et al., 2015).

All but one study (Masoumi et al., 2016) found that antenatal education was associated with a reduction of FOC in women (El-Malky, El-Homosy, Ashour, & Shehada, 2018; Gökçe İsbir, İnci, Önal, & Dikmen-Yıldız, 2016; Haapio et al., 2017; Karabulut, Coşkuner Potur, Doğan Merih, Cebeci Mutlu, & Demirci, 2016; Kizilirmak & Başer, 2016; Serçekuş & Başkale, 2016; Taheri et al., 2014) and men (Bergström et al., 2013). Antenatal education was also associated with a change in birth preferences (Ozdemir et al., 2018), an increased likelihood of having a spontaneous vaginal birth (Rouhe et al., 2015) and a more positive birth experience (Rouhe et al., 2013).

#### Enhanced midwifery care

Three studies examined enhanced midwifery care, including a continuity of care model (*n* = 2; Hildingsson, Rubertsson, Karlström, & Haines, 2018; Hildingsson, Karlström, Rubertsson, & Haines, 2019; Hildingsson et al., 2019; Lyberg & Severinsson, 2010a, 2010b) or a midwife-led visit to a delivery suite (Sydsjö et al., 2014). One study had a high risk of bias, and the remaining had a medium risk of bias. The results showed that continuity of care was evaluated positively by women (Lyberg & Severinsson, 2010a, 2010b) and was associated with reduced FOC, increased satisfaction with care (Hildingsson et al., 2018; Hildingsson et al., 2019a) and an improved birth experience (Hildingsson, Rubertsson, Karlström, & Haines, 2019b). The impact of visiting the delivery suite with a midwife prior to labour was associated with a shorter duration of labour for multiparous women. However, rate of emergency CS was higher in the intervention group (Sydsjö et al., 2014).

#### Alternative interventions

Twelve studies examined alternative interventions, including relaxation (e.g. guided relaxation, meditation/mindfulness, hypnobirthing; Baleghi, Akerdi, & Pasha, 2016; Bulez et al., 2019; Byrne, Hauck, Fisher, Bayes, & Schutze, 2014; Fisher et al., 2012; Hunter et al., 2011; Pour-Edalati et al., 2019), exercise (*n* = 2; Guder, Yalvac, & Vural, 2018; Guszkowska, 2014), art therapy (*n* = 2; Sezen & Ünsalver, 2019; Wahlbeck, Kvist, & Landgren, 2018) or group psychodynamic (Saisto, Toivanen, Salmela-Aro, & Halmesmäki, 2006), role play (Navaee & Abedian, 2015) and heart rate monitoring (Narita, Shinohara, & Kodama, 2018). Ten studies had control groups; no studies were RCTs.

Relaxation-style interventions and art therapy were associated with a decrease in FOC (Baleghi et al., 2016; Bulez et al., 2019; Byrne et al., 2014; Fisher et al., 2012; Pour-Edalati et al., 2019; Sezen & Ünsalver, 2019; Wahlbeck et al., 2018); however, all but one of these studies had a medium or high risk of bias. Results for the remaining interventions were less clear. Role play reduced FOC; however, a reduction in FOC was also found in the control group (Navaee & Abedian, 2015). One exercise intervention including yoga, Pilates and body ball found no impact on FOC (Guszkowska, 2014) whereas another Pilates course was found to reduce FOC (Guder et al., 2018). Lastly, heart rate variability biofeedback was associated with lower FOC over time (from 32–34 to 36–67 weeks) (Narita et al., 2018).

#### Interventions during labour

Two interventions were carried out during labour, one had low risk of bias and the other had medium risk. One used an RCT to evaluate the effect of EFT [exposure therapy and somatic stimulation using acupressure points (i.e. tapping) compared to breathing awareness or SMC and found a reduction of FOC in the intervention group (Irmak Vural & Aslan, 2019)]. Another RCT using marching songs and cheerful music during labour found no impact on FOC, sense of power and self-control (Phumdoung, Youngvanichsate, & Wongmuneeworn, 2011).

### Quantitative meta-analysis

#### Characteristics of studies

Twenty-eight papers from 22 studies were included in the meta-analysis (see online Supplementary Table S4). The majority of papers (79%) were carried out in Scandinavian (Sweden or Finland) or Middle-Eastern (Iran, Turkey or Egypt) countries. A variety of types of intervention were employed, the most frequent being antenatal education (*n* = 10; 36%). The intensity of interventions varied from six or more sessions (high intensity) to two or fewer sessions. Control groups were mostly routine care, although in six papers the control was some form of counselling. Design of the studies included randomised controlled trials (*n* = 12), quasi-experimental (*n* = 14) and a pilot study (*n* = 2). Papers were recent with a median publication year of 2017.

#### Publication bias

**Fig. 2.**
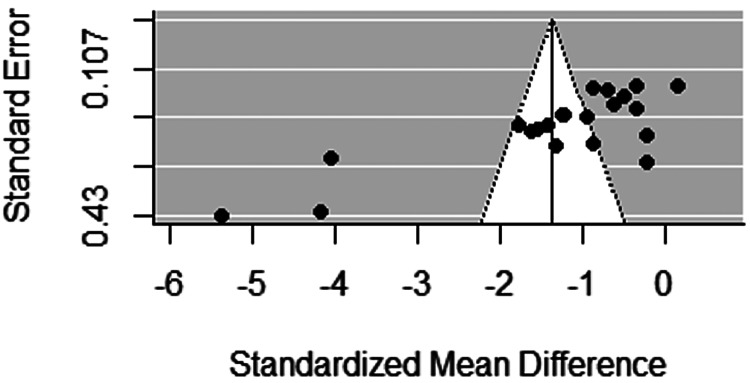
Publication bias funnel plot.

The trim and fill analysis and funnel plot showed that there were 0 estimated studies missing indicating that there is no significant funnel plot asymmetry (model estimate = −1.3673; s.e. = 0.3006, *p* < 0.0001; *τ*^2^ = 1.837; s.e. = 0.600). As can be seen from Fig. 2, studies are evenly distributed around the central effect size and there is no evidence of studies that have non-significant or opposite results being omitted.

#### Types of outcome

For the majority of papers (*n* = 23), the outcome was a measure of FOC. The remaining papers (*n* = 12) recorded the mode of childbirth (whether or not a CS). There was overlap between papers (see online Supplementary Table S4).

#### Fear of childbirth

Most papers (*n* = 16) used the Wijma Delivery Expectancy/Experience Questionnaire to measure FOC (Wijma et al., 1998) (W-DEQ).

**Fig. 3.**
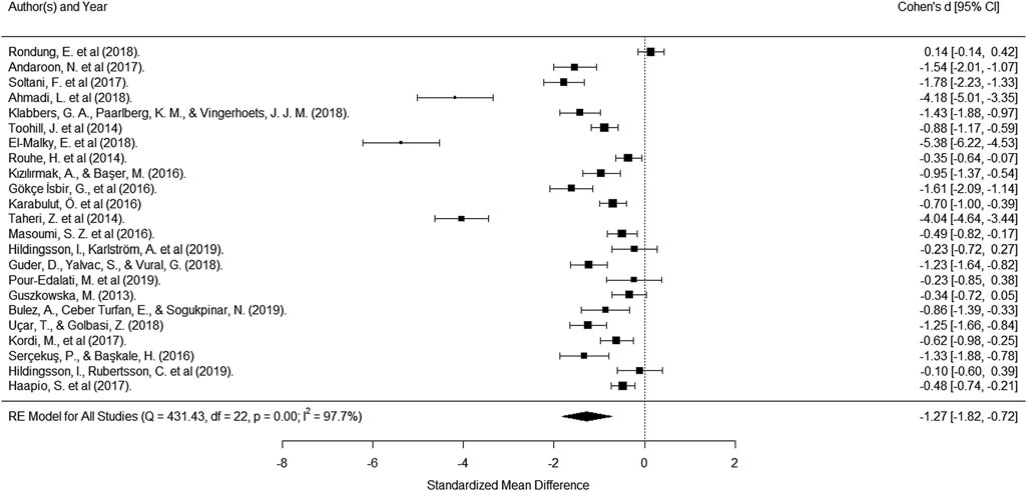
Fear of childbirth forest plot.

The overall mean effect size was −1.27 (*z* = −4.53, *p* < 0.0001) indicating that women in the intervention group experienced much less FOC compared to those in the control group. There was, however, considerable heterogeneity in the size of the effect reported (*Q* = 431.43, *p* < 0.0001; *I*^2^ = 97.7). All papers reported a lower FOC following intervention except Rondung et al. (2018) (Fig. 3 and Table 1). In three studies (Ahmadi et al., 2018; El-Malky et al., 2018; Taheri et al., 2014), the effect of the intervention was greater than four standard deviations, and an analysis of studentised residual indicated that these were significant outliers. When the analysis was re-run without these outliers, the overall effect was smaller but large and remained significant (*d* = −0.80, *z* = −6.50, *p* < 0.0001). Significant heterogeneity of effect sizes remained (*Q* = 138.80, *p* < 0.0001; *I*^2^ = 87.3).

**Table 1.** Effect sizes for fear of childbirth interventions on FOC reduction

Author and year	*n*	Country	Risk of bias	Effect size Cohen's *d*	95% CI
CBT					
Kordi et al. (2017)	120	Iran	Low	−0.62	−0.98 to −0.25
Rondung et al. (2018)	258	Sweden	Low	0.14	−0.14 to 0.42
Uçar and Golbasi (2019)	111	Turkey	Medium	−1.25	−1.66 to −0.84
			**Range:**	**0.14, −0.62**	
Other talking therapies					
Ahmadi et al. (2018)	71	Iran	Medium	−4.18	−5.01 to −3.35
Andaroon et al. (2017)	93	Iran	Low	−1.54	−2.01 to −1.07
Klabbers et al. (2018)	134	The Netherlands	Medium	−1.43	−1.88 to −0.97
Soltani et al. (2017)	106	Iran	Medium	−1.78	−2.23 to −1.33
Toohill et al. (2014)	339	Australia	Low	−0.88	−1.17 to −0.59
			**Range:**	**−0.88, −4.18**	
Antenatal education					
El-Malky et al. (2018)	100	Egypt	High	−5.38	−6.22 to −4.53
Haapio et al. (2017)	659	Finland	Low	−0.48	−0.74 to −0.21
Gökçe İsbir et al. (2016)	90	Turkey	Medium	−1.61	−2.09 to −1.14
Karabulut et al. (2016)	192	Turkey	Medium	−0.70	−1.00 to −0.39
Kizilirmak and Başer (2016)	99	Turkey	Medium	−0.95	−1.37 to −0.54
Masoumi et al. (2016)	160	Iran	Low	−0.49	−0.82 to −0.17
Rouhe et al. (2015)	330	Australia	Low	−0.35	−0.64 to −0.07
Serçekuş and Başkale (2016)	63	Turkey	Medium	−1.33	−1.88 to −0.78
Taheri et al. (2014)	130	Iran	Low	−4.04	−4.64 to −3.44
			**Range:**	**−0.35, −5.38**	
Enhanced midwifery care					
Hildingsson et al. (2019a)^a^	70	Sweden	Medium	−0.23	−0.72 to 0.27
Hildingsson and Rubertsson (2019)^a^	70	Sweden	Medium	−0.10	−0.60 to 0.39
			**Range:**	**−0.10, −0.23**	
Alternative interventions					
Bulez et al. (2019)	60	Turkey	High	−0.86	−1.39 to −0.33
Guder et al. (2018)	108	Cyprus	Medium	−1.23	−1.64 to −0.82
Guszkowska (2014)	109	Poland	Medium	−0.34	−0.72 to 0.05
Pour-Edalati et al. (2019)	41	Iran	Medium	−0.23	−0.85 to 0.38
			**Range:**	**−0.23, −0.86**	

a Results from the same study.

Examination of moderators found country was the only moderator of effectiveness (Table 2). Studies in Middle-Eastern countries reported much larger effect sizes (*d* = −1.54, *z* = −2.23, *p* = 0.026, *n* = 14) than those from Scandinavian countries (*d* = −0.20, *z* = −0.34, *p* = 0.731, *n* = 5). Whether the intervention was antenatal education or other talking therapies did not significantly moderate the effect. It was not possible to examine other intervention types because there were too few studies.

**Table 2.** Moderators of effect of intervention on fear of childbirth (*k* = 23 unless otherwise stated)

Moderators	*Q*M	*Q*E
Risk of bias	0.18	413.17**
Country (*k* = 19)	4.98*	303.00**
Whether or not RCT	1.10	302.41**
Sample: only women with FOC *v*. all women	2.37	407.84**
Sample: nulliparous *v*. mixed parity (*k* = 21)	1.43	408.31**
Intensity of intervention (*k* = 19)	0.63	358.19**
Other talking therapies *v*. antenatal education (*k* = 14)	0.09	314.46**

*p < 0.05; **p < 0.01.

#### Mode of delivery

**Fig. 4.**
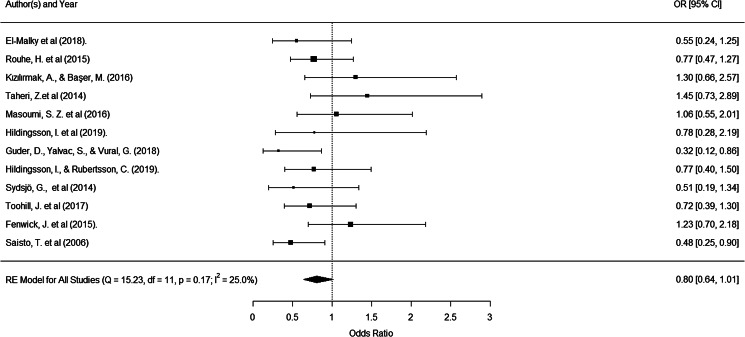
Caearean section by choice forest plot.

Twelve studies recorded whether birth was by CS (Fig. 4 and Table 3). Overall, the odds of a CS were lower in the intervention group compared to the control group, but this difference was not statistically significant (OR = 0.80, *z* = −1.85, *p* = 0.065). The test for homogeneity was also not significant [*Q* (df = 11) = 15.23, *p* = 0.172]. Examination of studentised residuals indicated there were no significant outliers.

**Table 3.** Effect sizes for fear of childbirth interventions on CS rate reduction

Author and year	*n*	Country	Risk of bias	Effect size (Cohen's *d*)	95% CI
CBT					
Hildingsson and Rubertsson (2019)	258	Sweden	Low	0.77	0.40–1.50
Sydsjö et al. (2015)	612	Sweden	Medium	0.51	0.19–1.34
			**Range:**	**0.51, 0.77**	
Other talking therapies					
Fenwick et al. (2015)^a^	184	Australia	Low	1.23	0.70–2.18
Toohill et al. (2017)^a^	184	Australia	Low	0.72	0.39–1.30
			**Range:**	**0.72, 1.23**	
Antenatal education					
El-Malky et al. (2018)	100	Egypt	High	0.55	0.24–1.25
Kizilirmak and Başer (2016)	99	Turkey	Medium	1.30	0.66–2.57
Masoumi et al. (2016)	160	Iran	Low	1.06	0.55–2.01
Rouhe et al. (2015)	330	Finland	Low	0.77	0.47–1.27
Taheri et al. (2014)	130	Iran	Low	1.45	0.73–2.89
			**Range:**	**0.55, 1.45**	
Enhanced midwifery care					
Hildingsson et al. (2019a)	70	Sweden	Medium	0.78	0.28–2.19
			**Range:**	**0.78**	
Alternative interventions					
Guder et al. (2018)	108	Cyprus	Medium	0.32	0.12–0.86
Saisto et al. (2006)	187	Finland	Medium	0.48	0.25–0.90
			**Range:**	**0.32, 0.48**	

a Results from the same study.

Two significant modifiers were found: country and type of intervention (Table 4). In Scandinavian countries, the odds of a CS were significantly lower in the intervention group (OR 0.66, 95% CI 0.49–0.90), whereas in Middle-Eastern countries, women in the intervention group were more likely to have a CS (OR 1.07, 95% CI 0.76–1.52). Those who undertook talking therapies were less likely to have a CS (OR 0.48, 95% CI 0.48–0.90) compared to those who received antenatal education (OR 1.01, 95% CI 0.78–1.31).

**Table 4.** Moderators of effect of intervention on fear of childbirth (*k* = 12 unless otherwise stated)

Moderators	*Q*M	*Q*E
Risk of bias	1.61	13.34
Country (*k* = 9)	4.11*	5.53
Whether or not RCT (*k* = 11)	2.36	9.77
Sample: only FOC *v*. all women	0.71	14.14
Sample: nulliparous *v*. mixed parity (*k* = 11)	1.44	12.62
Intensity of intervention (*k* = 8)	1.65	11.19
Other talking therapies *v*. antenatal education (*k* = 7)	4.52	5.31

*p < 0.05; **p < 0.01.

## Discussion

### Main findings

This review aimed to identify interventions that reduced FOC and CS rates. The review identified 66 papers from 48 studies that investigated six types of intervention. Most studies were carried out in non-English speaking countries and only two recruited fathers-to-be. The quality of the studies varied with 30 out of 48 having a medium risk of bias.

Results from the meta-analysis suggest that most interventions, regardless of the approach, reduce FOC. This is in line with results from the qualitative synthesis which found that within each intervention approach, the majority of studies found a reduction in FOC. The studies that did not find a reduction of FOC were counselling run by midwives (Ryding et al., 2003), self-reported counselling (Larsson et al., 2015), antenatal education classes run by midwives with a focus on physical wellbeing (Masoumi et al., 2016), an exercise intervention including yoga, pilates and body ball (Guszkowska, 2014) and listening to marching songs during labour (Phumdoung et al., 2011).

The effect of interventions on CS rates was varied and insignificant overall, with studies of CBT (Larsson et al., 2019) finding no reduction in CS rates, and enhanced midwifery care finding increased likelihood of CS (Sydsjö et al., 2014). Talking therapies were more consistently associated with reduced CS rates whether delivered by telephone (Fenwick et al., 2015; Toohill et al., 2017) or face-to-face (Ahmadi et al., 2018). Results from the moderator analysis support this, in that women who undertook talking therapies were less likely to have a CS compared to those who received antenatal education. This suggests talking therapies may be potentially effective for reducing CS rates in women with FOC. However, the majority of these studies had a medium risk of bias and the results were not completely consistent with two studies finding that other talking therapies were associated with an increased risk of CS (Henriksen et al., 2018; Sydsjö et al., 2012). More research is therefore needed.

### Interpretation

The results from the qualitative and quantitative analysis of this review suggest that most intervention approaches investigated reduce FOC. Only five studies across all intervention groups found null results in terms of FOC reduction, compared to 25 studies that found a reduction in FOC. It may therefore be the delivery of an intervention, rather than the theoretical underpinning that makes it effective at reducing FOC. This is supported by a recent meta-synthesis of women's experiences of interventions for FOC (O'Connell, Khashan, & Leahy-Warren, 2020) that found interventions with a woman-centred ethos, where women felt listened to and where a trusting relationship was able to develop were crucial for women to move from fear to ownership of childbirth. Further, most women felt empowered when they attended FOC interventions, and this was facilitated by supportive alliances, education and birth choices. It could therefore be suggested that providing women with a supportive space to explore their fear is more important than providing a specific approach in terms of reducing FOC. However, more research is needed to understand whether this is the case.

Another potential reason for this finding is that there is a wide range of different fears involved in FOC as well as individualised responses (Wigert et al., 2020). Further, studies do not always differentiate between primary tokophobia (women who have not given birth before) and secondary (women who have given birth before) tokophobia. This is an important distinction to make, because secondary tokophobia usually develops after a previous traumatic birth (Bhatia & Jhanjee, 2012) and, although not specific to childbirth, there is an evidence base about how trauma and PTSD can be treated [National Institute for Health and Care Excellence (NICE), 2018].

The findings from previous systematic reviews that identified interventions with an educational component as being promising at reducing FOC (Moghaddam Hosseini et al., 2018; Stoll et al., 2018; Striebich et al., 2018) warrants further exploration. This review found that interventions with an educational component were the most studied (*n* = 19 studies), but not the most effective. It is therefore possible that the findings from previous systematic reviews can be explained by the fact that interventions with an educational component appeared to be most promising because they had been studied more. However, it is not clear if this is the only explanation, therefore more high-quality RCTs are needed to examine the effectiveness of different types and components of interventions on FOC.

The varied and insignificant effect of FOC interventions on CS rates is unsurprising given the many physiological, psychosocial, contextual, organisational and cultural factors that influence whether a woman wants, requires or has a CS. This is illustrated by the finding that country of study moderated the effect of FOC interventions on CS rates, suggesting cultural and organisational context are important. For example, FOC interventions were found to reduce CS rates amongst women in Scandinavian countries and increase rates in Middle Eastern countries (Iran, Egypt and Turkey). This is perhaps reflective of maternity care in these countries. Iran and Egypt both provide a medicalised model of maternity care, with high mortality and very little antenatal education (Aghlmand et al., 2008; Choices and Challenges in Changing Childbirth Research Network, 2005; El-Kurdy, Hassan, Hassan, & El-Nemer, 2017; TorkZahrani, 2008). Further, the rates of CS are higher in these countries at 48–52% (Azami-Aghdash, Ghojazadeh, Dehdilani, Mohammadi, & Asl Amin Abad, 2014; Ministry of Health and Population et al., 2015; Santas & Santhas, 2018) than in Scandinavian countries (15–20%) (Pyykönen et al., 2017). Additionally, it is important to note that in some cases, a planned CS can improve women's birth experience, such as where there is not sufficient time for FOC intervention, or where FOC has not improved following intervention. A planned CS may be the next most appropriate clinical step and can allow for women to feel a sense of control of their birth experience. This suggests individual, cultural and organisational norms may influence whether FOC interventions affect CS rates, and the wider care environment should be taken into consideration when selecting a FOC intervention.

### Strengths and limitations

A strength of this review is the broad inclusion criteria, meaning all identified studies on FOC interventions were included. Another strength is the meta-analysis which provides a novel quantitative understanding of the effectiveness of FOC interventions in more robustly designed studies. Limitations of the review include the decision to only double screen 10% of abstracts and full texts. This may have meant some papers were missed; however, the high concordance of the double screening makes this seem unlikely. Further limitations are the low number of studies with different intervention types that could be included in the meta-analysis. There was great heterogeneity of the effect sizes for the FOC outcome across the studies (e.g. *Q* = 431.43; *Q* = 138.80). This makes interpretation of the results more difficult. Classification of interventions was challenging because they were complex interventions with multiple components. Some components could overlap between intervention categories. Components of interventions were also minimally described in many papers. Similarly, the methodological quality of studies was varied, with 38 out of the 48 studies had medium to high risk of bias. The way studies were carried out was also variable. For example, different outcome measures were used making it difficult to compare the baseline levels of FOC in all studies. Furthermore, those that used the WDEQ-A and were comparable recruited women with a range of WDEQ-A scores making results difficult to interpret (*M* = 29.7–130). Additionally, where participant demographics were reported, women were often highly educated, married and white, meaning these results may not be generalisable to those from more marginalised populations. Due to these limitations, the results from these studies should be interpreted cautiously.

## Conclusion

Overall, this review suggests that interventions for FOC are effective in reducing FOC but have variable effects on CS rates. FOC interventions do not affect CS rates overall, but this is influenced by cultural and organisational context. High-quality RCTs of different FOC interventions that not only evaluate type of intervention but also determine which components of interventions are most effective for particular presentations are needed in order to design optimal FOC interventions.

## Supplementary material

For supplementary material accompanying this paper visit https://doi.org/10.1017/S0033291721002324. click here to view supplementary material

## References

[ref1] Aghlmand, S., Akbari, F., Lameei, A., Mohammad, K., Small, R., & Arab, M. (2008). Developing evidence-based maternity care in Iran: A quality improvement study. BMC Pregnancy and Childbirth, 8(1), 1–8. doi: 10.1186/1471-2393-8-20. 18554384 PMC2443790

[ref2] Ahmadi, L., Karami, S., Faghihzadeh, S., Jafari, E., Oskoei, A., & Kharaghani, R. (2018). Effect of couples counseling based on the problem-solving approach on the fear of delivery, self-efficacy, and choice of delivery mode in the primigravid women requesting elective cesarean section. Preventative Care in Nursing & Midwifery Journal, 7(4), 32–40. URL: http://zums.ac.ir/nmcjournal/article-1-751-en.html

[ref3] Andaroon, N., Kordi, M., Kimiaei, S., & Esmaeily, H. (2017). The effect of individual counseling program by a midwife on fear of childbirth in primiparous women. Journal of Education and Health Promotion, 6, 1–7. doi: https://doi.org/10.4103/jehp.jehp_172_16. 10.4103/jehp.jehp_172_16 PMC5747210 29296598

[ref4] Andersson, L., Sundström-Poromaa, I., Wulff, M., Åström, M., & Bixo, M. (2004). Implications of antenatal depression and anxiety for obstetric outcome. Obstetrics and Gynecology, 104(3), 467–476. doi: 10.1097/01.AOG.0000135277.04565.e9. 15339755

[ref5] Azami-Aghdash, S., Ghojazadeh, M., Dehdilani, N., Mohammadi, M., & Asl Amin Abad, R. (2014). Prevalence and causes of cesarean section in Iran: Systematic review and meta-analysis. Iranian Journal of Public Health, 43(5), 545-555. PMID: 26060756 26060756 PMC4449402

[ref6] Baleghi, M., Akerdi, E. M., & Pasha, Y. Z. (2016). The effect of relaxation on childbirth and an increase in natural childbirth. Journal of Babol University of Medical Sciences, 18(8), 14–19. URL: http://jbums.org/article-1-6191-en.html

[ref7] Baylis, R., Ekdahl, J., Haines, H., & Rubertsson, C. (2019). Women's experiences of internet-delivered cognitive behaviour therapy (iCBT) for fear of birth. Women and Birth, 33(3), e227–e233. doi: 10.1016/j.wombi.2019.05.006. 31160244

[ref8] Beebe, K. R., Lee, K. A., Carrieri-Kohlman, V., & Humphreys, J. (2007). The effects of childbirth self-efficacy and anxiety during pregnancy on prehospitalization labor. Journal of Obstetric, Gynecologic & Neonatal Nursing, 36(5), 410–418. 10.1111/j.1552-6909.2007.00170.x 17880311

[ref9] Bergström, M., Rudman, A., Waldenström, U., & Kieler, H. (2013). Fear of childbirth in expectant fathers, subsequent childbirth experience and impact of antenatal education: Subanalysis of results from a randomized controlled trial. Acta Obstetricia et Gynecologica Scandinavica, 92(8), 967–973. doi: 10.1111/aogs.12147. 23590647

[ref10] Bhatia, M., & Jhanjee, A. (2012). Tokophobia: A dread of pregnancy. Industrial Psychiatry Journal, 21(2), 158. doi: 10.4103/0972-6748.119649. 24250052 PMC3830168

[ref11] Borenstein, M., Hedges, L. V., Higgins, J. P. T., & Rothstein, H. R. (2009). Introduction to meta-analysis. John Wiley & Sons: Chichester, United Kingdom. Retrieved from 10.1002/9780470743386.

[ref12] Bulez, A., Ceber Turfan, E., & Sogukpinar, N. (2019). Evaluation of the effect of hypnobirthing education during antenatal period on fear of childbirth. The European Research Journal, 5(2), 350–354. doi: 10.18621/eurj.371102.

[ref13] Byrne, J., Hauck, Y., Fisher, C., Bayes, S., & Schutze, R. (2014). Effectiveness of a mindfulness-based childbirth education pilot study on maternal self-efficacy and fear of childbirth. Journal of Midwifery and Women's Health, 59(2), 192–197. doi: 10.1111/jmwh.12075. 24325752

[ref14] Capik, A., & Durmaz, H. (2018). Fear of childbirth, postpartum depression, and birth-related variables as predictors of posttraumatic stress disorder after childbirth. Worldviews on Evidence-Based Nursing, 15(6), 455–463. 10.1111/wvn.12326. 30281197

[ref15] Choices and Challenges in Changing Childbirth Research Network (2005). Routines in facility-based maternity care: Evidence from the Arab World. BJOG : An International Journal of Obstetrics and Gynaecology, 112(9), 1270–1276. doi: 10.1111/j.1471-0528.2005.00710.x. 16101607 PMC1457116

[ref16] D'Cruz, L., & Lee, C. (2014). Childbirth expectations: An Australian study of young childless women. Journal of Reproductive and Infant Psychology, 32(2), 199–211. doi: 10.1080/02646838.2013.875134.

[ref17] Dehghani, M., Sharpe, L., & Khatibi, A. (2014). Catastrophizing mediates the relationship between fear of pain and preference for elective caesarean section. European Journal of Pain, 18(4), 582–589. doi: 10.1002/j.1532-2149.2013.00404.x. 24115590

[ref18] Dencker, A., Nilsson, C., Begley, C., Jangsten, E., Mollberg, M., Patel, H., … Sparud-Lundin, C. (2019). Causes and outcomes in studies of fear of childbirth: A systematic review. Women and Birth, 32(2), 99–111. doi: 10.1016/j.wombi.2018.07.004. 30115515

[ref19] Ministry of Health and Population, El-Zanty and Associates, & The DHS Program ICF International. (2015). *Egypt Demographic and Health Survey* 2014. Ministry of Health and Population and ICF International. Retrieved from https://dhsprogram.com/pubs/pdf/FR302/FR302.pdf.

[ref20] Duval, S., & Tweedie, R. (2000). Trim and fill: A simple funnel-plot-based method of testing and adjusting for publication bias in meta-analysis. Biometrics, 56(2), 455–463. doi: 10.1111/j.0006-341X.2000.00455.x. 10877304

[ref21] Eide, K. T., Morken, N. H., & Bærøe, K. (2019). Maternal reasons for requesting planned cesarean section in Norway: A qualitative study. BMC Pregnancy and Childbirth, 19(1), 102. doi: 10.1186/s12884-019-2250-6. 30922267 PMC6440101

[ref22] El-Kurdy, R., Hassan, S. I., Hassan, N. F., & El-Nemer, A. (2017). Antenatal education on childbirth self-efficacy for Egyptian primiparous women: A randomized control trial. IOSR Journal of Nursing and Health Science, *6,* 15–23. 10.9790/1959-0604021523.

[ref23] El-Malky, E., El-Homosy, S., Ashour, E., & Shehada, Y. (2018). Effectiveness of antenatal nursing intervention on childbirth's fears, psychological – wellbeing and pregnancy outcomes in primipara's women. Journal of Nursing Science, 4(2), 17–24.

[ref24] Fairbrother, N., Thordarson, D. S., & Stoll, K. (2018). Fine tuning fear of childbirth: The relationship between Childbirth Fear Questionnaire subscales and demographic and reproductive variables. Journal of Reproductive and Infant Psychology, 36(1), 15–29. doi: 10.1080/02646838.2017.1396300. 29517300

[ref25] Fenwick, J., Hauck, Y., Downie, J., & Butt, J. (2005). The childbirth expectations of a self-selected cohort of Western Australian women. Midwifery, 21(1), 23–35. doi: 10.1016/j.midw.2004.07.001. 15740814

[ref26] Fenwick, J., Toohill, J., Gamble, J., Creedy, D. K., Buist, A., Turkstra, E., … Ryding, E. L. (2015). Effects of a midwife psycho-education intervention to reduce childbirth fear on women's birth outcomes and postpartum psychological wellbeing. BMC Pregnancy and Childbirth, 15(1), 1–8. doi: 10.1186/s12884-015-0721-y. 26518597 PMC4628230

[ref27] Fisher, C., Hauck, Y., Bayes, S., & Byrne, J. (2012). Participant experiences of mindfulness-based childbirth education: A qualitative study. BMC Pregnancy and Childbirth, 12(1), 1–10. doi: 10.1186/1471-2393-12-126. 23145970 PMC3534482

[ref28] Furuta, M., Sandall, J., & Bick, D. (2012). A systematic review of the relationship between severe maternal morbidity and post-traumatic stress disorder. BMC Pregnancy and Childbirth, 12, 125. doi: 10.1186/1471-2393-12-125. 23140343 PMC3582425

[ref29] Gökçe İsbir, G., İnci, F., Önal, H., & Dikmen-Yıldız, P. (2016). The effects of antenatal education on fear of childbirth, maternal self-efficacy and post-traumatic stress disorder (PTSD) symptoms following childbirth: An experimental study. Applied Nursing Research, 32, 227–232. doi: 10.1016/j.apnr.2016.07.013. 27969033

[ref30] Guder, D., Yalvac, S., & Vural, G. (2018). The effect of pregnancy Pilates-assisted childbirth preparation training on childbirth fear and neonatal outcomes: A quasi-experimental/quantitative research. Quality and Quantity, 52(6), 2667–2679.

[ref31] Guszkowska, M. (2014). The effect of exercise and childbirth classes on fear of childbirth and locus of labor pain control. Anxiety, Stress and Coping, 27(2), 176–189. doi: 10.1080/10615806.2013.830107. 24199962

[ref32] Haapio, S., Kaunonen, M., Arffman, M., & Åstedt-Kurki, P. (2017). Effects of extended childbirth education by midwives on the childbirth fear of first-time mothers: An RCT. Scandinavian Journal of Caring Sciences, 31(2), 293–301. doi: 10.1111/scs.12346. 27439382

[ref33] Haines, H., Pallant, J. F., Karlström, A., & Hildingsson, I. (2011). Cross-cultural comparison of levels of childbirth-related fear in an Australian and Swedish sample. Midwifery, 27(4), 560–567. doi: 10.1016/j.midw.2010.05.004. 20598787

[ref34] Hall, W. A., Stoll, K., Hutton, E. K., & Brown, H. (2012). A prospective study of effects of psychological factors and sleep on obstetric interventions, mode of birth, and neonatal outcomes among low-risk British Columbian women. BMC Pregnancy and Childbirth, 12(1), 1–10. doi: 10.1186/1471-2393-12-78. 22862846 PMC3449197

[ref35] Halvorsen, L., Nerum, H., Sørlie, T., & Øian, P. (2010). Does counsellor's attitude influence change in a request for a caesarean in women with fear of birth? Midwifery, 26(1), 45–52. doi: 10.1016/j.midw.2008.04.011. 18621452

[ref36] Henriksen, L., Borgen, A., Risløkken, J., & Lukasse, M. (2018). Fear of birth: Prevalence, counselling and method of birth at five obstetrical units in Norway. Women and Birth, 33(1), 97–104. doi: 10.1016/j.wombi.2018.11.008. 30522889

[ref37] Higgins, J. P. T., Altman, D. G., Gøtzsche, P. C., Jüni, P., Moher, D., Oxman, A. D., … Sterne, J. A. C. (2011). The Cochrane Collaboration's tool for assessing risk of bias in randomised trials. BMJ *(*Online*)*, 343, 1-0. doi: 10.1136/bmj.d5928. PMC3196245 22008217

[ref38] Hildingsson, I., Karlström, A., Rubertsson, C., & Haines, H. (2019a). Women with fear of childbirth might benefit from having a known midwife during labour. Women and Birth, 32(1), 58–63. doi: 10.1016/j.wombi.2018.04.014. 29773474

[ref39] Hildingsson, I., & Rubertsson, C. (2019). Childbirth experiences among women with fear of birth randomized to internet-based cognitive therapy or midwife counseling. Journal of Psychosomatic Obstetrics & Gynecology, 41(3), 205–214. doi: 10.1080/0167482X.2019.1634047. 31244352

[ref40] Hildingsson, I., Rubertsson, C., Karlström, A., & Haines, H. (2018). Caseload midwifery for women with fear of birth is a feasible option. Sexual and Reproductive Healthcare, 16, 50–55. doi: 10.1016/j.srhc.2018.02.006. 29804775

[ref41] Hildingsson, I., Rubertsson, C., Karlström, A., & Haines, H. (2019b). A known midwife can make a difference for women with fear of childbirth- birth outcome and women's experiences of intrapartum care. Sexual and Reproductive Healthcare, 21, 33–38. doi: 10.1016/j.srhc.2019.06.004. 31395231

[ref42] Hildingsson, I., Thomas, J., Karlström, A., Olofsson, R. E., & Nystedt, A. (2010). Childbirth thoughts in mid-pregnancy: Prevalence and associated factors in prospective parents. Sexual & Reproductive Healthcare, 1(2), 45–53. doi: 10.1016/j.srhc.2009.11.003. 21122596

[ref43] Hunter, L., Bormann, J., Belding, W., Sobo, E. J., Axman, L., Reseter, B. K., … Miranda Anderson, V. (2011). Satisfaction and use of a spiritually based mantram intervention for childbirth-related fears in couples. Applied Nursing Research, 24(3), 138–146. doi: 10.1016/j.apnr.2009.06.002. 20974063

[ref44] Irmak Vural, P., & Aslan, E. (2019). Emotional freedom techniques and breathing awareness to reduce childbirth fear: A randomized controlled study. Complementary Therapies in Clinical Practice, 35, 224–231. doi: 10.1016/j.ctcp.2019.02.011. 31003663

[ref45] Jokić-Begić, N., Žigić, L., & Nakić Radoš, S. (2014). Anxiety and anxiety sensitivity as predictors of fear of childbirth: Different patterns for nulliparous and parous women. Journal of Psychosomatic Obstetrics and Gynecology, 35(1), 22–28. doi: 10.3109/0167482X.2013.866647. 24328559

[ref46] Karabulut, O., Coşkuner Potur, D., Doğan Merih, Y., Cebeci Mutlu, S., & Demirci, N. (2016). Does antenatal education reduce fear of childbirth? International Nursing Review, 63(1), 60–67. doi: 10.1111/inr.12223. 26612181

[ref47] Khedr, N., & Eldeen, M. (2017). Effect of healthy instructions on reducing pregnant women's fear of normal delivery and preferences for cesarean delivery. American Journal of Nursing Science, 6(3), 176–184. doi: https://doi.org/0.11648/j.ajns.20170603.15.

[ref48] Kinsella, M. T., & Monk, C. (2009). Impact of maternal stress, depression and anxiety on fetal neurobehavioral development. Clinical Obstetrics and Gynecology, 52(3), 425. doi: 10.1097/GRF.0b013e3181b52df1. 19661759 PMC3710585

[ref49] Kizilirmak, A., & Başer, M. (2016). The effect of education given to primigravida women on fear of childbirth. Applied Nursing Research, 29, 19–24. doi: 10.1016/j.apnr.2015.04.002. 26856483

[ref50] Klabbers, G. A., Paarlberg, K. M., & Vingerhoets, J. J. M. (2018). Does haptotherapy benefit mother-child bonding in women with high fear of childbirth? International Journal of Haptonomy and Haptotherapy, 3(1), 1–7.

[ref51] Klabbers, G. A., Wijma, K., Paarlberg, K. M., Emons, W. H. M., & Vingerhoets, A. J. J. M. (2019). Haptotherapy as a new intervention for treating fear of childbirth: A randomized controlled trial. Journal of Psychosomatic Obstetrics and Gynecology, 40(1), 38–47. doi: 10.1080/0167482X.2017.1398230. 29157055

[ref52] Kordi, M., Bakhshi, M., Masoudi, S., & Esmaily, H. (2017). Effect of a childbirth psychoeducation program on the level of fear of childbirth in primigravid women. Evidence Based Care Journal, 7(3), 26–34. doi: 10.22038/EBCJ.2017.25676.1575.

[ref53] Kulkarni, A., Wright, E., & Kingdom, J. (2014). Web-based education and attitude to delivery by caesarean section in nulliparous women. Journal of Obstetrics and Gynaecology Canada, 36(9), 768–775. doi: 10.1016/S1701-2163(15)30478-3. 25222355

[ref54] Larsson, B., Hildingsson, I., Ternström, E., Rubertsson, C., & Karlström, A. (2019). Women's experience of midwife-led counselling and its influence on childbirth fear: A qualitative study. Women and Birth, 32(1), e88–e94. doi: 10.1016/j.wombi.2018.04.008. 29709431

[ref55] Larsson, B., Karlström, A., Rubertsson, C., & Hildingsson, I. (2015). The effects of counseling on fear of childbirth. Acta Obstetricia et Gynecologica Scandinavica, 94(6), 629–636. doi: 10.1111/aogs.12634. 25772528

[ref56] Larsson, B., Karlström, A., Rubertsson, C., Ternström, E., Ekdahl, J., Segebladh, B., & Hildingsson, I. (2017). Birth preference in women undergoing treatment for childbirth fear: A randomised controlled trial. Women and Birth, 30(6), 460–467. doi: 10.1016/j.wombi.2017.04.004. 28495462

[ref57] Lipsey, M. W., & Wilson, D. B. (2001). Practical meta-analysis. Applied Social Research Methods Series. SAGE Publications. doi: 10.1016/j.autneu.2007.06.087.

[ref58] Logtenberg, S. L. M., Verhoeven, C. J., Rengerink, K. O., Sluijs, A. M., Freeman, L. M., Schellevis, F. G., & Mol, B. W. (2018). Pharmacological pain relief and fear of childbirth in low risk women; secondary analysis of the RAVEL study. BMC Pregnancy and Childbirth, 18(1), 1–9. doi: 10.1186/s12884-018-1986-8. 30144796 PMC6109320

[ref59] Lucas, G., Olander, E. K., Ayers, S., & Salmon, D. (2019). No straight lines – young women's perceptions of their mental health and wellbeing during and after pregnancy: A systematic review and meta-ethnography. BMC Women's Health, 19(1), 1–17. doi: 10.1186/s12905-019-0848-5. 31806005 PMC6896260

[ref60] Lukasse, M., Schei, B., Ryding, E. L., & Bidens Study Group. (2014). Prevalence and associated factors of fear of childbirth in six European countries. Sexual & Reproductive Healthcare, 5(3), 99–106. doi: 10.1016/j.srhc.2014.06.007. 25200969

[ref61] Lukasse, M., Vangen, S., Ãian, P., & Schei, B. (2011). Fear of childbirth, women's preference for cesarean section and childhood abuse: A longitudinal study. Acta Obstetricia et Gynecologica Scandinavica, 90(1), 33–40. doi: 10.1111/j.1600-0412.2010.01024.x. 21275913

[ref62] Lyberg, A., & Severinsson, E. (2010a). Fear of childbirth: Mothers’ experiences of team-midwifery care – a follow-up study. Journal of Nursing Management, 18(4), 383–390. doi: 10.1111/j.1365-2834.2010.01103.x. 20609042

[ref63] Lyberg, A., & Severinsson, E. (2010b). Midwives’ supervisory styles and leadership role as experienced by Norwegian mothers in the context of a fear of childbirth. Journal of Nursing Management, 18(4), 391–399. doi: 10.1111/j.1365-2834.2010.01083.x. 20609043

[ref64] Masoumi, S. Z., Kazemi, F., Oshvandi, K., Jalali, M., Esmaeili-Vardanjani, A., & Rafiei, H. (2016). Effect of training preparation for childbirth on fear of normal vaginal delivery and choosing the type of delivery among pregnant women in Hamadan, Iran: A randomized controlled trial. Journal of Family & Reproductive Health, 10(3), 115, PMID: 28101112. 28101112 PMC5241355

[ref65] Matinnia, N., Faisal, I., Juni, M. H., Herjar, A. R., Moeini, B., & Osman, Z. J. (2015). Fears related to pregnancy and childbirth among primigravidae who requested caesarean versus vaginal delivery in Iran. Maternal and Child Health Journal, 19(5), 1121–1130. 25269852 10.1007/s10995-014-1610-0

[ref66] Melender, H. L. (2002). Experiences of fears associated with pregnancy and childbirth: A study of 329 pregnant women. Birth (Berkeley, Calif), 29(2), 101–111. doi: 10.1046/j.1523-536X.2002.00170.x. 12051188

[ref67] Moghaddam Hosseini, V., Nazarzadeh, M., & Jahanfar, S. (2018). Interventions for reducing fear of childbirth: A systematic review and meta-analysis of clinical trials. Women and Birth, 31(4), 254–262. doi: 10.1016/j.wombi.2017.10.007. 29126794

[ref68] Moher, D., Shamseer, L., Clarke, M., Ghersi, D., Liberati, A., Petticrew, M., … Stewart, L. A. (2015). Preferred reporting items for systematic review and meta-analysis protocols (PRISMA-P) 2015 statement. Systematic Reviews, 4(1), 1–9. doi: 10.1186/2046-4053-4-1. 25554246 PMC4320440

[ref69] Narita, Y., Shinohara, H., & Kodama, H. (2018). Resting heart rate variability and the effects of biofeedback intervention in women with low-risk pregnancy and prenatal childbirth fear. Applied Psychophysiology Biofeedback, 43(2), 113–121. doi: 10.1007/s10484-018-9389-1. 29476282

[ref70] National Institute for Health and Care Excellence (NICE) (2018). Post-traumatic stress disorder: NICE Guidelines NG116. Appendix C: Evidence reviews for psychological, psychosocial and other non-pharmacological intervention for the prevention of PTSD in adults. Retrieved 23 March 2021, from https://www.nice.org.uk/guidance/NG116. 32757552

[ref71] Navaee, M., & Abedian, Z. (2015). Effect of role play education on primiparous women's fear of natural delivery and their decision on the mode of delivery. Iranian Journal of Nursing and Midwifery Research, 20(1), 41–46. PMID: 25709689. PMC4325412 25709689

[ref72] Nerum, H., Halvorsen, L., Sørlie, T., & Øian, P. (2006). Maternal request for cesarean section due to fear of birth: Can it be changed through crisis-oriented counseling? Birth (Berkeley, Calif), 33(3), 221–228. doi: 10.1111/j.1523-536X.2006.00107.x. 16948722

[ref73] NHS England (2019). Perinatal mental health. Retrieved 23 March 2021, from https://www.england.nhs.uk/mental-health/perinatal/.

[ref74] Nieminen, K., Andersson, G., Wijma, B., Ryding, E. L., & Wijma, K. (2016). Treatment of nulliparous women with severe fear of childbirth via the Internet: A feasibility study. Journal of Psychosomatic Obstetrics and Gynecology, 37(2), 37–43. doi: 10.3109/0167482X.2016.1140143. 26918752

[ref75] Nieminen, K., Malmquist, A., Wijma, B., Ryding, E. L., Andersson, G., & Wijma, K. (2015). Nulliparous pregnant women's narratives of imminent childbirth before and after internet-based cognitive behavioural therapy for severe fear of childbirth: A qualitative study. BJOG: An International Journal of Obstetrics and Gynaecology, 122(9), 1259–1265. doi: 10.1111/1471-0528.13358. 25817045

[ref76] Nieminen, K., Stephansson, O., & Ryding, E. L. (2009). Women's fear of childbirth and preference for cesarean section – a cross-sectional study at various stages of pregnancy in Sweden. Acta Obstetricia et Gynecologica Scandinavica, 88(7), 807–813. doi: 10.1080/00016340902998436. 19488882

[ref77] Nilsson, C., Hessman, E., Sjöblom, H., Dencker, A., Jangsten, E., Mollberg, M., … Begley, C. (2018). Definitions, measurements and prevalence of fear of childbirth: A systematic review. BMC Pregnancy and Childbirth, 18(1), 1–15. doi: 10.1186/s12884-018-1659-7. 29329526 PMC5766978

[ref78] Nilsson, C., Lundgren, I., Karlström, A., & Hildingsson, I. (2012). Self reported fear of childbirth and its association with women's birth experience and mode of delivery: A longitudinal population-based study. Women and Birth, 25(3), 114–121. 21764400 10.1016/j.wombi.2011.06.001

[ref79] O'Connell, M. A., Khashan, A. S., & Leahy-Warren, P. (2020). Women's experiences of interventions for fear of childbirth in the perinatal period: A meta-synthesis of qualitative research evidence. Women and Birth, *34*, e309–e321. doi: 10.1016/j.wombi.2020.05.008. 32522443

[ref80] O'Connell, M. A., Leahy-Warren, P., Kenny, L. C., O'Neill, S. M., & Khashan, A. S. (2019). The prevalence and risk factors of fear of childbirth among pregnant women: A cross-sectional study in Ireland. Acta Obstetricia et Gynecologica Scandinavica, 98(8), 1014–1023. doi: 10.1111/aogs.13599. 30821844

[ref81] O'Connell, M. A., Leahy-Warren, P., Khashan, A. S., Kenny, L. C., & O'Neill, S. M. (2017). Worldwide prevalence of tocophobia in pregnant women: Systematic review and meta-analysis. Acta Obstetricia et Gynecologica Scandinavica, 96(8), 907–920. doi: 10.1111/aogs.13138. 28369672

[ref82] Ozdemir, M. E., Cilingir, I. U., Ilhan, G., Yildiz, E., & Ohanoglu, K. (2018). The effect of the systematic birth preparation program on fear of vaginal delivery and quality of life. Archives of Gynecology and Obstetrics, 298(3), 561–565. doi: 10.1007/s00404-018-4835-0. 29961135

[ref83] Pearson, R. M., Melotti, R., Heron, J., Joinson, C., Stein, A., Ramchandani, P. G., & Evans, J. (2012). Disruption to the development of maternal responsiveness? The impact of prenatal depression on mother-infant interactions. Infant Behavior and Development, 35(4), 613–626. doi: 10.1016/j.infbeh.2012.07.020. 22982260

[ref84] Phumdoung, S., Youngvanichsate, S., & Wongmuneeworn, W. (2011). The effects of instrumental marching and cheerful music on women's sense of power, self-control, fear of childbirth, and second-stage duration during their second stage of labor. Songklanagarind Medical Journal, 29(4), 163–173.

[ref85] Pour-Edalati, M., Moghadam, N., Shahesmaeili, A., & Salehi-Nejad, P. (2019). Exploring the effect of mindfulness-based stress reduction on childbirth fear among single-child mothers in the city of Kerman, Iran (2017): A clinical trial study. Medical Surgery Nursing Journal, 7(4), e90247. doi: 10.5812/msnj.90247.

[ref86] Pyykönen, A., Gissler, M., Løkkegaard, E., Bergholt, T., Rasmussen, S. C., Smárason, A., … Tapper, A. M. (2017). Cesarean section trends in the Nordic countries – a comparative analysis with the Robson classification. Acta Obstetricia et Gynecologica Scandinavica, 96(5), 607–616. doi: 10.1111/aogs.13108. 28176334

[ref87] Rachman, S. (1977). The conditioning theory of fear acquisition: A critical examination. Behaviour Research and Therapy, 15(5), 375–387. 612338 10.1016/0005-7967(77)90041-9

[ref88] Räisänen, S., Lehto, S. M., Nielsen, H. S., Gissler, M., Kramer, M. R., & Heinonen, S. (2013). Fear of childbirth predicts postpartum depression: A population-based analysis of 511 422 singleton births in Finland. BMJ Open, 3(11), 1-7. doi: 10.1136/bmjopen-2013-004047. PMC3845069 24293208

[ref89] Reck, C., Zimmer, K., Dubber, S., Zipser, B., Schlehe, B., & Gawlik, S. (2013). The influence of general anxiety and childbirth-specific anxiety on birth outcome. Archives of Women's Mental Health, 16(5), 363–369. doi: 10.1007/s00737-013-0344-0. 23558948

[ref90] Rondung, E., Ternström, E., Hildingsson, I., Haines, H. M., Sundin, Ö, Ekdahl, J., … Rubertsson, C. (2018). Comparing internet-based cognitive behavioral therapy with standard care for women with fear of birth: Randomized controlled trial. Journal of Medical Internet Research, 5(3), e10420. doi: 10.2196/10420. PMC6109226 30097422

[ref91] Rondung, E., Thomtén, J., & Sundin, Ö (2016). Psychological perspectives on fear of childbirth. Journal of Anxiety Disorders, 44, 80–91. 27788373 10.1016/j.janxdis.2016.10.007

[ref92] Rouhe, H., Salmela-Aro, K., Gissler, M., Halmesmäki, E., & Saisto, T. (2011). Mental health problems common in women with fear of childbirth. BJOG: An International Journal of Obstetrics and Gynaecology, 118(9), 1104–1111. doi: 10.1111/j.1471-0528.2011.02967.x. 21489127

[ref93] Rouhe, H., Salmela-Aro, K., Toivanen, R., Tokola, M., Halmesmäki, E., Ryding, E. L., & Saisto, T. (2015). Group psychoeducation with relaxation for severe fear of childbirth improves maternal adjustment and childbirth experience-a randomised controlled trial. Journal of Psychosomatic Obstetrics and Gynecology, 36(1), 1–9. doi: 10.3109/0167482X.2014.980722. 25417935

[ref94] Rouhe, H., Salmela-Aro, K., Toivanen, R., Tokola, M., Halmesmäki, E., & Saisto, T. (2013). Obstetric outcome after intervention for severe fear of childbirth in nulliparous women – randomised trial. BJOG: An International Journal of Obstetrics and Gynaecology, 120(1), 75–84. doi: 10.1111/1471-0528.12011. 23121002

[ref95] Ryding, E. L., Lukasse, M., Van Parys, A. S., Wangel, A. M., Karro, H., Kristjansdottir, H., … Laanpere, M. (2015). Fear of childbirth and risk of cesarean delivery: A cohort study in six European countries. Birth (Berkeley, Calif), 42(1), 48–55. doi: 10.1111/birt.12147. 25676793

[ref96] Ryding, E. L., Persson, A., Onell, C., & Kvist, L. (2003). An evaluation of midwives’ counseling of pregnant women in fear of childbirth. Acta Obstetricia et Gynecologica Scandinavica, 82(1), 10–17. doi: 10.1034/j.1600-0412.2003.820102.x. 12580833

[ref97] Ryding, E. L., Read, S., Rouhe, H., Halmesmäki, E., Salmela-Aro, K., Toivanen, R., … Saisto, T. (2018). Partners of nulliparous women with severe fear of childbirth: A longitudinal study of psychological well-being. Birth (Berkeley, Calif), 45(1), 88–93. doi: 10.1111/birt.12309. 28892237

[ref98] Ryding, E., Wijma, B., Wijma, K., & Rydhström, H. (1998). Fear of childbirth during pregnancy may increase the risk of emergency cesarean section. Acta Obstetricia et Gynecologica Scandinavica, 77(5), 542–547. 9654177

[ref99] Saisto, T., Salmela-Aro, K., & Nurmi, J. E. (2001). A randomized controlled trial of intervention in fear of childbirth. Obstetrics and Gynecology, 98(5), 820–826. doi: 10.1016/S0029-7844(01)01552-6. 11704175

[ref100] Saisto, T., Toivanen, R., Salmela-Aro, K., & Halmesmäki, E. (2006). Therapeutic group psychoeducation and relaxation in treating fear of childbirth. Acta Obstetricia et Gynecologica Scandinavica, 85(11), 1315–1319. doi: 10.1080/00016340600756920. 17091410

[ref101] Sambrook Smith, M., Lawrence, V., Sadler, E., & Easter, A. (2019). Barriers to accessing mental health services for women with perinatal mental illness: Systematic review and meta-synthesis of qualitative studies in the UK. BMJ Open, 9(1), e024803. 10.1136/bmjopen-2018-024803 PMC6347898 30679296

[ref102] Santas, G., & Santhas, F. (2018). Trends of caesarean section rates in Turkey. Journal of Obstetrics and Gynaecology, 38(5), 658–662. doi: 10.1080/01443615.2017.1400525. 29519178

[ref103] Serçekuş, P., & Başkale, H. (2016). Effects of antenatal education on fear of childbirth, maternal self-efficacy and parental attachment. Midwifery, 34, 166–172. 10.1016/j.midw.2015.11.016. 26656473

[ref104] Sezen, C., & Ünsalver, BÖ (2019). Group art therapy for the management of fear of childbirth. Arts in Psychotherapy, 64, 9–19. doi: 10.1016/j.aip.2018.11.007.

[ref105] Sjogren, B. (1998). Fear of childbirth and psychosomatic support – a follow up of 72 women. Acta Obstetricia Et Gynecologica Scandinavica, 77, 819–825. 9776595 10.1080/j.1600-0412.1998.770807.x

[ref106] Söderquist, J., Wijma, K., & Wijma, B. (2004). Traumatic stress in late pregnancy. Journal of Anxiety Disorders, 18(2), 127–142. doi: 10.1016/S0887-6185(02)00242-6. 15033212

[ref107] Soltani, F., Eskandari, Z., Khodakarami, B., Parsa, P., & Roshanaei, G. (2017). The effect of self-efficacy oriented counselling on controlling the fear of natural delivery in primigravida women. Journal of Pharmaceutical Sciences and Research, 9(10), 1757–1761.

[ref108] Stein, A., Pearson, R. M., Goodman, S. H., Rapa, E., Rahman, A., McCallum, M., … Pariante, C. M. (2014). Effects of perinatal mental disorders on the fetus and child. The Lancet, 384(9956), 1800–1819. doi: 10.1016/S0140-6736(14)61277-0. 25455250

[ref109] Stoll, K., & Hall, W. A. (2013). Attitudes and preferences of young women with low and high fear of childbirth. Qualitative Health Research, 23(11), 1495–1505. doi: 10.1177/1049732313507501. 24108088

[ref110] Stoll, K., Swift, E. M., Fairbrother, N., Nethery, E., & Janssen, P. (2018). A systematic review of nonpharmacological prenatal interventions for pregnancy-specific anxiety and fear of childbirth. Birth (Berkeley, Calif), 45(1), 7–18. doi: 10.1111/birt.12316. 29057487

[ref111] Striebich, S., Mattern, E., & Ayerle, G. M. (2018). Support for pregnant women identified with fear of childbirth (FOC)/tokophobia – a systematic review of approaches and interventions. Midwifery, 61, 97–115. doi: 10.1016/j.midw.2018.02.013. 29579696

[ref112] Sydsjö, G., Angerbjörn, L., Palmquist, S., Bladh, M., Sydsjö, A., & Josefsson, A. (2013). Secondary fear of childbirth prolongs the time to subsequent delivery. Acta Obstetricia et Gynecologica Scandinavica, 92(2), 210–214. doi: 10.1111/aogs.12034. 23066797

[ref113] Sydsjö, G., Bladh, M., Lilliecreutz, C., Persson, A. M., Vyoni, H., & Josefsson, A. (2014). Obstetric outcomes for nulliparous women who received routine individualized treatment for severe fear of childbirth – a retrospective case control study. BMC Pregnancy and Childbirth, 14(1), 1–7. doi: 10.1186/1471-2393-14-126. 24694283 PMC4234140

[ref114] Sydsjö, G., Blomberg, M., Palmquist, S., Angerbjörn, L., Bladh, M., & Josefsson, A. (2015). Effects of continuous midwifery labour support for women with severe fear of childbirth. BMC Pregnancy and Childbirth, 15(1), 1–5. doi: 10.1186/s12884-015-0548-6. 25976219 PMC4495950

[ref115] Sydsjö, G., Sydsjö, A., Gunnervik, C., Bladh, M., & Josefsson, A. (2012). Obstetric outcome for women who received individualized treatment for fear of childbirth during pregnancy. Acta Obstetricia et Gynecologica Scandinavica, 91(1), 44–49. doi: 10.1111/j.1600-0412.2011.01242.x. 21787365

[ref116] Taheri, Z., Mazaheri, M. A., Khorsandi, M., Hassanzadeh, A., & Amiri, M. (2014). Effect of educational intervention on self-efficacy for choosing delivery method among pregnant women in 2013. International Journal of Preventive Medicine, 5(1), 1247, PMID: 25400882. 25400882 PMC4223943

[ref117] Talge, N. M., Neal, C., & Glover, V. (2007). Antenatal maternal stress and long-term effects on child neurodevelopment: How and why? Journal of Child Psychology and Psychiatry and Allied Disciplines, 48(3–4), 245–261. doi: 10.1111/j.1469-7610.2006.01714.x. PMC11016282 17355398

[ref118] Toohill, J., Callander, E., Gamble, J., Creedy, D. K., & Fenwick, J. (2017). A cost effectiveness analysis of midwife psycho-education for fearful pregnant women – a health system perspective for the antenatal period. BMC Pregnancy and Childbirth, 17(1), 1–7. doi: 10.1186/s12884-017-1404-7. 28693447 PMC5504805

[ref119] Toohill, J., Fenwick, J., Gamble, J., Creedy, D. K., Buist, A., Turkstra, E., & Ryding, E. L. (2014). A randomized controlled trial of a psycho-education intervention by midwives in reducing childbirth fear in pregnant women. Birth (Berkeley, Calif), 41(4), 384–394. doi: 10.1111/birt.12136. PMC4257571 25303111

[ref120] TorkZahrani, S. (2008). Commentary: Childbirth education in Iran. The Journal of Perinatal Education, 17(3), 51–54. doi: 10.1624/105812408X329601. 19436412 PMC2517184

[ref121] Tsui, M. H., Pang, M. W., Melender, H. L., Xu, L., Lau, T. K., & Leung, T. N. (2007). Maternal fear associated with pregnancy and childbirth in Hong Kong Chinese women. Women & Health, 44(4), 79–92. doi: 10.1300/J013v44n04_05. 17456465

[ref122] Turkstra, E., Mihala, G., Scuffham, P. A., Creedy, D. K., Gamble, J., Toohill, J., & Fenwick, J. (2017). An economic evaluation alongside a randomised controlled trial on psycho-education counselling intervention offered by midwives to address women's fear of childbirth in Australia. Sexual and Reproductive Healthcare, 11, 1–16. doi: 10.1016/j.srhc.2016.08.003. 28159118

[ref123] Uçar, T., & Golbasi, Z. (2019). Effect of an educational program based on cognitive behavioral techniques on fear of childbirth and the birth process. Journal of Psychosomatic Obstetrics and Gynecology, 40(2), 146–155. doi: 10.1080/0167482X.2018.1453800. 29583056

[ref124] Wahlbeck, H., Kvist, L. J., & Landgren, K. (2018). Gaining hope and self-confidence – an interview study of women's experience of treatment by art therapy for severe fear of childbirth. Women and Birth, 31(4), 299–306. doi: 10.1016/j.wombi.2017.10.008. 29100948

[ref125] Wigert, H., Nilsson, C., Dencker, A., Begley, C., Jangsten, E., Sparud-Lundin, C., … Patel, H. (2020). Women's experiences of fear of childbirth: A metasynthesis of qualitative studies. International Journal of Qualitative Studies on Health and Well-being, 15(1), 1704484. doi: 10.1080/17482631.2019.1704484. 31858891 PMC6968519

[ref126] Wijma, K., Ryding, E. L., & Wijma, B. (2002). Predicting psychological well-being after emergency caesarean section: A preliminary study. Journal of Reproductive and Infant Psychology, 20(1), 25–36. doi: 10.1080/02646830220106776.

[ref127] Wijma, K., Söderquist, J., & Wijma, B. (1997). Posttraumatic stress disorder after childbirth: A cross sectional study. Journal of Anxiety Disorders, 11(6), 587–597. doi: 10.1016/S0887-6185(97)00041-8. 9455721

[ref128] Wijma, K., Wijma, B., & Zar, M. (1998). Psychometric aspects of the W-DEQ; A new questionnaire for the measurement of fear of childbirth. Journal of Psychosomatic Obstetrics and Gynaecology, 19(2), 84–97. doi: 10.3109/01674829809048501. 9638601

