# Supplementary material for: Interventions to treat fear of childbirth in pregnancy: a systematic review and meta-analysis
Source: Psychol Med. 2021 Jun 25;51(12):1964–77. doi: 10.1017/S0033291721002324 (PMC8426149; doi:10.1017/S0033291721002324)
Supplement: Supplementary file 1 [file S0033291721002324sup001.docx]

**Interventions to treat fear of childbirth in pregnancy: a systematic review and meta-analysis**

**Supplementary Information**

**Appendix S1: Search Strategy**

Search terms used for systematic searches

| Population | Fear of Childbirth | Caesarean section by maternal choice | Measurement |
| --- | --- | --- | --- |
| *natal | Fear of childbirth | Caesarean section | *therapy |
| *partum | Tocophobia | Caesarean* | Counselling |
| Pregnan* | Tokophobia | Abdom* deliver* | Intervention |
| Birth |  | extraction | Psych* |
| Labour OR labor |  | C section |  |
| Parent* |  | c-section |  |
| Mother* |  | csection |  |
|  |  | AND |  |
|  |  | Patient preference |  |
|  |  | Choice |  |
|  |  | Choose |  |
|  |  | Request |  |
|  |  | prefer |  |
|  |  | Deci* |  |
|  |  | Seek |  |
|  |  | Plan |  |
|  |  | Elect* |  |
|  |  | Schedule* |  |
|  |  | Prearrange* |  |
|  |  | Non-emergency |  |
|  |  | Demand |  |

**Databases used for searching:**

MEDLINE, PsycINFO, PsychARTICLES, EMBASE, Cochrane Library, HMIC, Web of Science, PubMed and Scopus databased

**Table S1. PRISMA Checklist**

(Moher et al., 2016)

| **Section/topic** | **#** | **Checklist item** | **Reported on page #** |
| --- | --- | --- | --- |
| **TITLE** | | |  |
| Title | 1 | Identify the report as a systematic review, meta-analysis, or both. | 1 |
| **ABSTRACT** | | |  |
| Structured summary | 2 | Provide a structured summary including, as applicable: background; objectives; data sources; study eligibility criteria, participants, and interventions; study appraisal and synthesis methods; results; limitations; conclusions and implications of key findings; systematic review registration number. | 2 |
| **INTRODUCTION** | | |  |
| Rationale | 3 | Describe the rationale for the review in the context of what is already known. | 3-4 |
| Objectives | 4 | Provide an explicit statement of questions being addressed with reference to participants, interventions, comparisons, outcomes, and study design (PICOS). | 4 |
| **METHODS** | | |  |
| Protocol and registration | 5 | Indicate if a review protocol exists, if and where it can be accessed (e.g., Web address), and, if available, provide registration information including registration number. | 4 |
| Eligibility criteria | 6 | Specify study characteristics (e.g., PICOS, length of follow-up) and report characteristics (e.g., years considered, language, publication status) used as criteria for eligibility, giving rationale. | 4 |
| Information sources | 7 | Describe all information sources (e.g., databases with dates of coverage, contact with study authors to identify additional studies) in the search and date last searched. | Appendix S1 |
| Search | 8 | Present full electronic search strategy for at least one database, including any limits used, such that it could be repeated. | Appendix S1 |
| Study selection | 9 | State the process for selecting studies (i.e., screening, eligibility, included in systematic review, and, if applicable, included in the meta-analysis). | 4 |
| Data collection process | 10 | Describe method of data extraction from reports (e.g., piloted forms, independently, in duplicate) and any processes for obtaining and confirming data from investigators. | 5 |
| Data items | 11 | List and define all variables for which data were sought (e.g., PICOS, funding sources) and any assumptions and simplifications made. | 5 |
| Risk of bias in individual studies | 12 | Describe methods used for assessing risk of bias of individual studies (including specification of whether this was done at the study or outcome level), and how this information is to be used in any data synthesis. | 5 |
| Summary measures | 13 | State the principal summary measures (e.g., risk ratio, difference in means). | 5-6 |
| Synthesis of results | 14 | Describe the methods of handling data and combining results of studies, if done, including measures of consistency (e.g., I^2^) for each meta-analysis. | 5-6 |

**Table S2. Studies included in the review**

|  |  | **Cognitive/cognitive behavioural therapy (k = 11 from 6 studies)** | | | | | | | | |
| --- | --- | --- | --- | --- | --- | --- | --- | --- | --- | --- |
| **Paper No** | **Study no** | **Citation**  **Country**  **Design** | **Participant characteristics**  **Number in sample (n)** | **Measure used to determine FOC** | **Intervention** | **Control** | **Randomised?** | **Outcome measure** | **Outcome** | **Risk of bias** |
| 1 | 1 | (K. Nieminen et al., 2015)  Sweden  Qualitative data from pre-post interventional | Age: M = 29.5  100% cohabiting  80% university degree  N = 15 | WDEQ-A^1^ ≥ 100 | **Internet based CBT principle programme:** consisted of psycho-education, cognitive restructuring, exposure both imaginary and in vivo, as well as relapse prevention. The programme used weekly modules and included questions about the information but also tasks to self-train daily. | n/a | n/a | Semi-structured task carried out within the internet-based intervention. Participants were asked open-ended questions about their upcoming labour and delivery. | Prior to intervention participants saw their own role as anxious, uncertain and lonely. Post intervention participants saw their role with more certainty, and as active subjects with coping mechanisms to deal with the birth. | Medium |
| 2 | 1 | (Katri Nieminen, Andersson, Wijma, Ryding, & Wijma, 2016)  Pre-post | Age M = 30.5,  96.5% cohabiting,  68% university degree  N = 28 | WDEQ-A^1^ ≥ 85 |  |  |  | FOC^1^ | There was a statistically significant decrease of FOC during treatment, with F(3.4, 48.2) = 27.35, p<0.0001. The Bonferroni post hoc correction revealed that the decrease started after module three (difference in W-DEQ sum score from pre-treatment to module four: 16.6± 6.4, p = 0.02), which corresponded with the introduction of exposure tasks. |  |
| 3 | 2 | (T. Saisto, Salmela-Aro, & Nurmi, 2001)  Finland  RCT | **Intervention (n = 85):**  Age M = 31.2 (SD = 5.1)  91% married/cohabiting. 71.4% work full time.  **Control (n = 91):**  Age M = 31.9 (SD = 4.8)  92% married or cohabiting  70.8% work full time | Five or more affirmative answers on Areskog questionnaire^2^ or request for cesarean. | **Intensive therapy:** Intensive therapy was an intensive cognitive therapy delivered by obstetrician with training and cognitive therapy and the psychology of childbirth. The main principles of the therapy are to focus on one target problem involving the active role of the therapist and reformulation of the problem during a limited time.  **Intervention details:** M = 3.8 sessions with obstetrician and M = 1 with midwife. | Yes | **Conventional therapy:** M = 2 sessions, standard information about birth and pros and cons of vaginal and caesarean delivery | Birth mode  Birth related concerns^9^  FOC^2^ | **Birth mode:** Caesarean for psychosocial reasons was chosen by 20 women in the intensive therapy group (36% of those originally requesting it) and by 26 women in the conventional therapy group (41%) (p = .05).  Labour was shorter in intensive group (M = 6.83 hours) compared to conventional group (M = 8.56 hours) (p = .039)  **Birth related concerns:** T1: Before randomization the women in the intensive therapy group mentioned birth related concerns more frequently (M = 0.56) than did those in conventional therapy group (M = 0.39). After therapy birth-related concerns in the intensive therapy group had decreased, but in the conventional therapy group they had increased (P = .022).  **FOC**  No significant difference in birth related fear between groups, although the intensive group had a tendency for lower levels of fear. Fear of labour pain decreased in intensive therapy group (from M = 4.76 to M = 4.46), compared with the conventional therapy group (increase from M = 4.4 to M = 4.5) (P = .041). | Medium |
| 4 | 3 | (Larsson et al., 2017)  Sweden  RCT | **Intervention (n = 127)**  70.9% aged between 25-35  60.6% primiparous  95.3% living with partner  56.7% completed university or college  **Control (n = 131)**  73.3% aged between 25-35  58.8% primiparous  93.1% living with partner  50.4% completed university or college  p>.05 | Fear of birth scale^3^ ≥ 60 | **Internet based CBT (ICBT)**  The program uses an educative approach where fear is presented as an experience including cognition, behaviour and physical sensations. The participants are taught to identify different aspects of their emotional experience (fear), how to avoid negative emotions and how to use alternative strategies in terms of acceptance, mindfulness and exposure.  There were eight treatment modules. The modules consisted of text material and assignments closely related to the content for each speciﬁc module.  When the required assignment was completed for the active module, the psychologist gave the women written feedback via the portal.  In addition to this feedback system, women in the intervention group could communicate with their psychologist through the portal at any time for support. | **Counselling from midwives** | **Yes –** Computer based randomisation system | Birth preference  Birth outcomes  Satisfaction with treatment  Perceived effect of treatment | **Birth preference**  **Intervention:**  Pre – Vaginal 66%  Post – Vaginal 88%  **Control**  Pre – Vaginal 76%  Post – Vaginal 80%  Not sig  **Mode of birth**  **Intervention:**  74.1% vaginal birth  7.4% instrumental birth  3.7% planned caesarean  14.8% emergency caesarean  **Control**  58.8% vaginal birth  10% instrumental birth  10% planned caesarean  17% emergency caesarean  p>.05  **Satisfaction with treatment**  **Intervention**  61.4% less than satisfied  **Control**  26% less than satisfied  P<.001  **Perceived effect**  **Intervention**  56.8% fear increased/did not affect fear  **Control**  19.2% fear increased/did not affect fear  P<.001 | Low |
| 5*  ▲ | 3 | (Rondung et al., 2018) |  |  |  |  |  | Treatment adherence  FOC^3^ | **Treatment adherence**  81% commended treatment. Mean time logged onto portal was 39.96 minutes (SD = 49.88; Range = 1-244 minutes).  **FOC**  **Intervention:**  Pre-intervention M = 71.76 (SD = 5.73)  Post-intervention: M = 67.15 (SD = 8.62)  Follow up: M = 41.03 (SD =22.45)*  **Standard care**  Pre-intervention M = 69.92 (SD = 7.33)  Post-intervention M = 65.73 (SD = 9.65)  Follow up: M = 47.87 (SD =24.10)*  *P = .049 for follow up |  |
| 6 | 3 | (Baylis, Ekdahl, Haines, & Rubertsson, 2019) | Qualitative data from 19 women who received ICBT  Age: 25-38; 16 women university education; 14 women first time mothers |  |  |  |  | Qualitative data | Experiences of iCBT: useful, meaningful and helpful, BUT the intervention did not necessarily relate to their own specific fears.  Relationship over the internet: some women felt ICBT was positive, supportive and flexible. The majority would have preferred more face to face treatment and found it difficult to work alone. Some women found it time consuming especially if they had other children.  Helpful but not enough: Women reported ICBT was not helpful if they have already had negative experience of birth. Women also found maintaining motivation difficult |  |
| 7*  ► | 3 | (Hildingsson & Rubertsson, 2019) |  |  |  |  |  | Birth outcomes  Childbirth experience | **Birth outcomes**  **Intervention:**  Induction of labour: 22.2%  Vaginal birth: 70.7%  Positive/very positive birth experience: 54.8%  **Control:**  Induction of labour: 16.9%  Vaginal birth: 60.6%  8Positive/very positive birth experience: 50.0%  p>.05  **Childbirth experience**  There was no statistically significant difference in the perceptions of the birth experience, regardless of the treatment method. |  |
| 8 | 3 | (Larsson, Hildingsson, Ternström, Rubertsson, & Karlström, 2019) | 27 women who received midwife counselling as part of RCT  Majority aged between 24-38; 18 first time mothers; 16 had unassisted vaginal birth |  |  |  |  | Qualitative data | The majority of women expressed that counselling and the birth experience contributed to a less troublesome level of fear or that they now had the capacity to manage their fear. A few women stated that they had no worries or fears at all after the counselling and birth.  Improved attitude towards giving birth: The information that women received by the counselling midwives is described as crucial for feeling ‘safe in the uncertainty’. The preparedness, and in some cases the processing, gave the women an improved attitude toward the approaching birth and several thought that this preparation was an important part of their experiencing a positive birth.  The majority of women  would consider receiving counselling again in a future pregnancy if needed. |  |
| 9*  ▲ | 4 | (Uçar & Golbasi, 2019)  Turkey  Quasi-experimental – pre-post | **Intervention (n = 52)**  Age: M = 25.3 (SD = 4.0)  50% completed high school  92.3% not working  **Control (n = 59)**  Age: M = 25.7 (SD = 4.3)  45.8% completed high school  96.6% not working  No significant differences between groups. | WDEQ-A^1^ | **CBT & Educational programme**  The main purpose of the educational program was to enable pregnant women to cope with their childbirth fears using CBT within the scope of childbirth preparation education. The cognitive behavioral approaches used during the educational program were recording non-functional thoughts, questioning automatic thoughts, teaching the ABC model, question and answer, relaxation techniques, homework, demonstration and feedback.  All sessions were conducted in groups of five to nine participants. The program was completed in 3 weeks over six sessions that occurred once a week, two sessions per day. Each session took approximately 45 minutes with a 15-minute break before the second. | SMC | No - The pregnant women presenting to the polyclinic between December 2012 and March 2013 constituted the control group and those presenting between April and July 2013 constituted the intervention group. | FOC^1^  Obstetric outcomes | **FOC**  **Intervention:**  Pre: M = 63.9 (SD = 26.8)  Post: M = 39.4 (SD = 21.0)  T = 8.686, p <.001  **Control**  Pre: M = 58.4 (SD = 23.5)  Post: M = 63.5 (SD = 25.5)  T = 3.01, p = .004  There was also a significant difference between groups at post-test (t = 8.987, p <.001).  **Obstetric outcomes**  Pain severity: Intervention M = 6.3 (SD = 1.9); Control: M = 8.3 (SD = 1.4) (t = 6.009, p <.001)  Length of stage 2 labour in minutes: Intervention M = 22.4 (SD = 5.5); Control: M = 27.1 (SD = 12.6) (t = 2.182, p =.033).  Childbirth satisfaction: Intervention: M = 3.5 (SD = 1.0); Control M = 2.8 (SD = 1.2) (t = -3.404, p = .001).  No significant differences in induction of labour, amniotomy, episiotomy, laceration, or foetal distress. | Medium |
| 10*  ▲ | 5 | (Kordi, Bakhshi, Masoudi, & Esmaily, 2017)  Iran  RCT | **Intervention group (n = 60**)  Age M = 23.2 (SD =3.6)  41.7% completed high school  87.6% housewife  **Control (n = 62)**  Age M = 24.2 (SD = 4.4)  43.5% completed high school  80.6% housewife  p>.05 | WDEQA^1^≥66 | **Psychoeducational programme**  Session 1: Explaining delivery, emotional changes, dealing with stress, understanding relationship between thoughts, behaviours and feelings, motherhood skills, relaxation.  Session 2: Role of thoughts in behaviour, logical and illogical thoughts, ABC model, relaxation  Session 3: problem solving, self esteem, relaxation  90minutes per session, run by a psychologist. | SMC | Yes – draw bag | FOC^1^  Birth outcomes | **FOC**  Pre intervention:  Intervention M = 91.4 (SD = 20.4)  Control M = 88.0 (SD = 16.1) (P>.05)  Post intervention:  Intervention: M = 83.5 (SD = 21.7)  Control M = 92.6 (SD = 18.4)  P = .007  Also a significant difference between T1 & T1 for intervention group (p =.003)  **Birth outcomes**  No significant differences | Low |
| 11*  ► | 6 | (Sydsjö et al., 2015)  Sweden  Quasi-experimental | **Intervention (n = 181)**  70.6% aged 25-34; 19.4% had normal BMI;  42.5% low white-collar worker  **Control (n = 431)**  59.6% were aged 25-34; 26.5% normal BMI; 46% low white collar worker  Significant differences between age, BMI & employment group | DSM-IV^4^ | **Individualised counselling -**  based on psycho-education e.g. determine the woman’s knowledge on childbirth and carefully educate her in relaxation, explain the physiological features of panic and anxiety; and cognitive behaviour theory e.g. assess thoughts, measure feelings and discuss avoidance and how to alter the reactions on certain thoughts.  For most of the women, an individual visit to the delivery ward was part of the treatment as an exposure for the fear situation. The number of sessions attended by each woman was based on each woman’s individual needs. | SMC | No – women didn’t receive therapy because they didn’t have FOC | Obstetric outcomes | **Treatment outcomes**  The FOC women who had participated in counselling/  treatment had on the average 1.6 sessions with a midwife, 1 session with a physician and 0.6 sessions with a psychotherapist, thus 3.2 visits per woman (range 1–12 sessions).  **Obstetric outcomes**  **Unassisted vaginal delivery**: Intervention 51.7%; Control 62.9%  **Instrumental vaginal delivery**: Intervention: 14.4%; Control: 18.3%  **Emergency CS:** Intervention: 14.4%; Control 15.5%  **Elective CS:** Intervention: 19.4%; Control: 3.2%  P <.001 | Medium |
|  |  | **Other talking therapies (k = 16 papers from 12 studies)** | | | | | | | | |
| **Paper no.** | **Study no.** | **Citation**  **Country**  **Design** | **Participant characteristics**  **Number in sample (n)** | **Measure used to determine FOC** | **Intervention** | **Control** | **Randomised?** | **Outcome measure** | **Outcome** | **Risk of bias** |
| 12*  ▲ | 7 | (Ahmadi et al., 2018)  Iran  Quasi-experimental | **Control (n = 35)**  Age: Women: M = 25.58 (SD = 3.63)  Partners: M = 31.42 (SD = 3.21)  Education: Women: M = 14.24 years (SD = 2.54)  Partners: M = 14.00 years (SD = 2.64)  50.00% 5^th^ economic class  42.1% 3^rd^ social class  **Intervention (n = 36)**  Age: Women: M = 25.87 (SD = 3.62)  Partners: M = 30.89 (SD = 3.60)  Education: Women: M = 13.97 years (SD = 2.83)  Partners: M = 13.94 years (SD = 2.83)  52.60% 5^th^ economic class  52.6% 3^rd^ social class | WDEQ-A^1^ | **Couples counselling -**  3 weekly counselling sessions based on a problem-solving approach. Covered communication, education on labour and birth, causes of fears, overcoming negative self talk, and problem solving. | SMC | No | Women’s knowledge  Women’s FOC^1^  Women’s self-efficacy^5^  Men’s knowledge  Birth mode | **Women’s knowledge**  Improvement in both intervention (Pre: M = 5.86; SD = 1.72; Post: M = 9.25; SD = .93, p <.001) and control (Pre: M = 4.48; SD = 1.2; Post: M = 5.51; SD = 1.12, p <.001)  **Women’s FOC**  Reduction only in intervention group (Pre: M = 87.58; SD = 14.98; Post: M = 63.12; SD = 17.08, p <.001  **Women’s self-efficacy**  Improvement in both intervention (Pre: M = 79.50 SD = 6.88; Post: M = 135.53; SD = .3.83, p <.001) and control (Pre: M = 71.97; SD = 16.11; Post: M = 76.62; SD = 15.76, p <.001)  **Men’s knowledge**  Improvement in both intervention (Pre: M = 5.20; SD = 2.04; Post: M = 9.14; SD = .80, p <.001) and control (Pre: M = 4.11; SD = 1.09; Post: M = 5.28; SD = 79, p <.001)  **Birth mode**  36 women in the control, compared to 14 women in the intervention had a caesarean section (p <.001).  24 women in the intervention, compared to 2 in the control had a vaginal delivery (p<.001) | Medium |
| 13*  ▲ | 8 | (Andaroon, Kordi, Kimiaei, & Esmaeily, 2017)  Iran  RCT | No significant differences between groups in terms of education, occupation, or socioeconomic status  N = 93 (45 in intervention group) | WDEQ-A^1^ = 38-84 | **Individual counselling**  Individual counselling was done face to face. Covered topics such as expressing emotions, communication feelings, beliefs and expectations, strategies to reduce the fear.  3 sessions lasting 45-60 minutes | SMC | Yes – by draw | FOC | **Intervention:**  Pre: M = 62.87; SD = 12.66  During: M = 39.73; SD = 17.09  After birth: M = 35.89; SD = 12.64  **Control**  Pre: M = 63.89 (SD = 14.13)  During: M = 65.67 (SD = 15.02)  After birth: M = 61.69 (SD = 12.55)  Significant differences between groups during the intervention and after birth. | Low |
| 14 | 9 | (Halvorsen, Nerum, Sørlie, & Øian, 2010)  Norway  Non-experimental | Two samples of women referred to crisis counselling for their wish to have a CS because of their FOC  **Sample 1:**  Age M = 31 years (SD = 5.1),  85/86 married/cohabIting56/86 university college education  N = 86  **Sample 2:**  Age M = 31 years (SD = 6.5)  81/107 married cohabiting,  49/107 university education  N = 107 | Assessment of the following five dichotomous variable for grading fear of birth:(1)sleep problems(worries and nightmares)and difficulty concentrating; (2) physiological manifestations of anxiety, such as trembling, sweating, rapid pulse and respiration (3)little or no insight into what the fear of birth represented; (4)large degree of experienced loss of control and predictability of the impending birth; and(5)fear of dying during pregnancy or birth. If four or five criteria were present, the fear of birth was defined as ‘severe’, and if three were present, it was defined as ‘moderate’. | **Crisis oriented counselling:** conducted by 2 midwives with training in mental health. The counselling was individualised. Included initial thorough examination of current and previous mental health status. | n/a | n/a | Request for a CS | **Sample 1:**  86% didn’t want to have a CS after counselling,  70% had vaginal birth after counselling.  In sample 1 a multiple logistic regression showed that vaginal birth was positively associated with counselling by midwife A (OR = 12.44; p<0.001) and negatively associated with severe FOC (OR = 0.169; p<0.010).  **Sample 2:**  95% didn’t want a CS after counselling.  80% had vaginal birth after counselling.  In sample 2, vaginal birth was negatively associated with severe FOC (OR = 0.121; p < 0.004) and mental health problems (OR = 0.053; p ¼ 0.018). Counselling with a midwife was not a predictor. | Medium |
| 15 | 10 | (Henriksen, Borgen, Risløkken, & Lukasse, 2018)  Norway  Non-experimental | Women attending one of five hospitals in Norway, with or without FOC.  **Women with FOC (n =258):**  39.1% aged 31-35; 59.2% over 13 years of education;  20.3% with symptoms of depression  24.9% received hospital counselling for FOC  18.6% wanted CS  **Women without FOC (n = 1,887):**  38.5% aged 25-30  74% over 13 years of education  5.7% with symptoms of depression  6.6% received hospital counselling  5.2% wanted a caesarean  P<.05 for all listed demographics | WDEQ-A^1^ ≥ 85 | **Hospital counselling** - taken from woman’s health record about whether they had received hospital counselling. Differed across hospitals:  **Oslo:** Individual consultations with midwives/doctors; no special methods used; goal was to make an individual birth plan; no recorded number of hours; given to 20/63 (88.89%) of women with FOC.  **Dranmen**: individual consultations, used empathetic communication; goal to help each woman feel safe, prevent depression and feel that they can cope with motherhood, provided by midwives and obstetricians, 3-4 hours in total, given to 24/39 (61.54%) of women with FOC.  **Tromso**: individual consultations, offered cognitive therapy. main goal was to map why woman had FOC, teach them how to cope, help them to feel safe; provided by midwives or obstetricians, 4 hours per woman; given to 4/45 (57.78%) of women.  **Alesund**: individual consultations, no special method used, tried to identify women with previous difficult birth, provided by midwives and obstetrician, no record of hours, 16/61 (85.25%) of women received counselling.  **Trondheim**: individual consultations, mix of cognitive therapy and empathetic communication, goal for women to achieve personal growth and cope with pregnancy and birth, 1.75hr per woman, 12/51 (50.98%) of women received counselling. | No counselling | No | Birth outcomes | Of the women who had FOC (not did not necessarily receive counselling)  **Oslo:**  63.5% vaginal birth; 19% assisted vaginal; 15.9% elective CS; 1.6% emergency CS.  **Buskerud:**  71.7% vaginal birth; 10.5% assisted vaginal; 15.8% elective CS; 2.6% emergency CS.  Tromso:  68.9% vaginal; 6.7% assisted vaginal; 11.1% elective CS; 13.1% emergency CS.  Alesund: Vagina: 75.4%; assisted vaginal 4.9%; elective CS 13.1%; emergency CS: 6.6%.  Trondheim: Vaginal birth: 66.7%; assisted vaginal: 5.9%; elective CS: 13.7%; emergency CS: 13.7%  Women with FOC who had no counselling were more likely to have a vaginal birth (79.5% vs 58.2%), less likely to have a planned CS (5% vs 22.8%). | High |
| 16 | 11 | (Larsson, Karlström, Rubertsson, & Hildingsson, 2015)  Sweden  Non-experimental | 71.4% aged 25-35;  64% multiparous;  95.7% living with partner;  63.2% college/university education;  69.1% FOC  N = 70 | The question regarding childbirth fear was: “Worries and fears are common feelings among women when facing childbirth. To what extent do you experience worry and fear at present?” The women answered using a four-point rating scale ranging from “a great deal” to “not at all.” In the analysis the variables were dichotomized into “a great deal/very much” and somewhat/not at all. If answering after birth, the question was framed for future births. | **Counselling -** Participants were asked about any counselling they received 2 months after birth "did you receive counselling due to fear of give birth "yes/no". If answered yes they were asked to provide more information about who provided the counselling and their level of satisfaction. | No counselling | No | Type of counselling reported  FOC one year after counselling  Type of birth | **Type of counselling**  40/70 women received counselling delivered by a specially trained midwife. 21/70 given counselling by regular midwife  **FOC after counselling**  Women who received counselling were still more fearful of birth (40.7%) compared to those who didn’t receive counselling (13%).  There was no signiﬁcant change in fear from mid-pregnancy to 1 year after birth (p = 0.198). Women who received counselling expressed more negative birth experiences compared with the group without counselling 2 months after giving birth (OR 2.0, 95% CI 1.2–3.3).  **Type of birth**  Women who received counselling were more likely to plan a CS 2.1 vs 5.8%.  No significant differences in vaginal birth rates, instrumental birth rates or emergency section rates  36.2% vs 7.2% would prefer to have a planned c section next time | Medium |
| 17 | 12 | (Nerum, Halvorsen, Sørlie, & Øian, 2006)  Norway  Non-experimental | Women referred to crisis counselling because of their FOC  Age M = 31.3  85% multiparous  85% married/cohabiting  41% had college education or less  N = 86 | Assessment of the following five dichotomous variable for grading FOC:(1)sleep problems(worries and nightmares)and difficulty concentrating; (2) physiological manifestations of anxiety, such as trembling, sweating, rapid pulse and respiration (3)little or no insight into what the FOC represented; (4)large degree of experienced loss of control and predictability of the impending birth; and(5)fear of dying during pregnancy or birth. If four or five criteria were present, the FOC was defined as ‘severe’, and if three were present, it was defined as ‘moderate’. | **Crisis oriented counselling:** Emphasis on the following things - (a) good contact and alliance; (b) charting birth-related feelings and concerns; (c) charting the life situation, present and previous psychological and somatic health and obstetric risk; (d) formulating and processing anxiety and life events that could be activated by the impending birth; (f) distinguishing between birth as the provoking factor and difficult life themes as the cause of FOC; and (g) helping the woman to see solutions other than giving birth by planned caesarean. | n/a | n/a | Request for CS | After the intervention:  86% of women changed their thinking about mode of birth, and prepared themselves for vaginal birth. This was split as following:  93% low obstetric risk women changed their mind  83% high obstetric risk women changed their minds.  100% of women with moderate FOC changed their mind for having a CS after intervention.  79.3% of women with severe FOC changed their mind. | Medium |
| 18 | 13 | (Ryding, Persson, Onell, & Kvist, 2003)  Sweden  Quasi-experimental | Median age = 30 years  20/53 women requested as CS for their FOC  Women who had contacted the FOC team  N = 53 intervention  N = 53 comparison | Not clear, but all women were under the care of a FOC team – a team of midwives who have been trained in counselling. | **Counselling:** on the ward, run by midwives. Discussion of fears, building confidence, writing a birth plan | n/a | Comparison group that were selected by researchers and matched for parity | Childbirth experience^6^ | **Childbirth experience:**  **Intervention:** M = 44.3 (SD = 20.5)  **Comparison:** M = 29.7 (SD = 7.4)  The mean difference was 14.6 (95% CI 9.4–19.7, p0.0001.)  “Those women who had been treated for FOC reported a rather more frightening experience of delivery, and more frequent symptoms of post-traumatic stress related to delivery than did the women in the comparison group” p. 10. | Medium |
| 19*  ▲ | 14 | (Klabbers, Wijma, Paarlberg, Emons, & Vingerhoets, 2019)  The Netherlands  RCT | **Haptotherapy (n = 51)** 62.7% first time parents; 70.5% high school education; Age M = 32.8 (SD = 4.6); WDEQ-A M = 101.1.  **Psychoeducation (n = 39)**  43.6% first time parents; 53.8% high school education level; Age M = 31.8 (SD = 3.9); WDEQ-A M = 104.5  **Control (n = 44)**  56.8% first time mothers; 61.4% high school education; M = 32.6 (SD = 5.3); WDEQ-A M = 98.6 | WDEQ-A^1^ ≥ 85 | **Haptotherapy -** Haptotherapy claims to facilitate the development of specific skills changing the cognitive appraisal of giving birth and labeling childbirth as a more normal and positive life event, which may ultimately lower FOC. Consisted of training women in a combination of skills, which are taught in eight 1 h sessions between gestational week 20 and 36.  **Psycho-education via the Internet -** consisted of eight modules (and a brief test) during a period of 8 weeks between gestational week 20 and 36, providing information about the normal course of  pregnancy, labor and birth | SMC | Yes | Adherence to intervention | **Adherence to intervention**  Eleven assigned to the psychoeducation group switched to the haptotherapy group on their own initiative, as did 14 who had been assigned to the care as usual group.  32 participants dropped out (haptotherapy: n = 9; psychoeducation: n = 14, care as usual:n = 9.  **FOC**  Haptotherapy versus psychoeducation via Internet (mean difference in change = 8.75: p = .250)  Haptotherapy versus care as usual (mean difference in change = 11.09, p = .049). | Medium |
| 20 | 14 | (Klabbers, Paarlberg, & Vingerhoets, 2018) |  |  |  |  |  | Mother-child bonding^23^ | There were no differences between women in the hapotherapy arm compared to control and psychoeducation (p = .121).  However, haptotherapy improved mother-child bonding in women who had low mother-child bonding scores prior to the intervention. | Medium |
| 21*  ▲ | 15 | (Soltani, Eskandari, Khodakarami, Parsa, & Roshanaei, 2017)  Iran  RCT | **Intervention (n = 53)**  Age M = 25.5 (SD = 3.5)  **Control (n =53)**  Age M = 25.7 (SD = 4.6) | CAQ^8^ | **Self-efficacy oriented counselling -**  6 sessions lasting between 60-90 minutes. Covers vaginal delivery, goals, exercising, expressing successful experiences, hearing others experiences, religious prayers, replacing irrational thoughts | SMC | Yes – doesn’t say how | FOC^8^  Self-efficacy^8^ | **FOC**  There was a significant decrease in FOC in the intervention group compared to the control group.  **Self-efficacy**  Pre intervention  Intervention M = 246.43 (SD = 57.7)  Control: M = 258.67 (SD = 48.86) p>.05  Post-intervention  Intervention M = 292.6 (SD = 54.24)  Control: M = 2546.75 (SD = 54.22) p<.001 | Medium |
| 22 | 16 | (Sjogren, 1998)  Turkey  Non-experimental | **Intervention (n = 72)**  Age: Median = 33 (range 22-42)  44 women had given birth before  **Control (n = 72)**  Matched controls, demographics not reported. Authors report that “Marital status, educational level, and types of occupation (medical and health care, service, office, economics, computer work, technicians, child care, teaching and official positions) and involuntary infertility proved to be similar in the groups” | Not clear | **Psychotherapy -** A systematic obstetrical and psychological history was taken by the author (obstetrician/gynecologist, trained in psychotherapy).  The ability of the individual woman  to benefit from conventional psychotherapy was evaluated. If a woman was very embarrassed by talking about herself, or if she just wanted to discuss her right to decide the model of the impending delivery, it was decided it would be very difficult or impossible to help her by conventional, verbal psychotherapy. As a result of this evaluation, some women in the study group were referred to a psychotherapist.  The goals of the treatment were to identify the components of the anxiety, to reduce the anxiety and, if possible, to encourage the women to consider a vaginal delivery. The psychotherapy given by the therapist was eclectic, because this group of women was heterogeneous. | SMC | No – matched controls | 32-item questionnaire covering pregnancy, experience of birth, physical and mental health after delivery and health of infant. | Treatment acceptance  According to the assessment, twenty-four women were offered conventional psychotherapy and eighteen women accepted.  The mean number of therapeutic meetings of those who had psychotherapy was 8.6 (range 2–14).  **Feelings about delivery, well-being and worry during pregnancy**  The women in the intervention group regarded the pregnancy as a less positive experience (Intervention n = 28; Control n = 40, p =.017), felt emotionally unprepared for the delivery (Intervention n = 18; Control n = 8, p = .01); felt less feelings of vigor (Intervention: n = 44; Control n = 58, p = .002), poor psychic health (Intervention n = 37; Control n = 17, p = .038); more worry about their own health (Intervention n = 18; Control n = 9, p = .038).  experience, and remembered significantly less well-being and more worry  **Emotional experience of delivery**  Delivery easier than expected (Intervention n = 43; Control n = 24, p = .004)  Felt in control of delivery (Intervention n = 46; control n = 29, p =.024)  Women in intervention group were slightly more anxious of their own death (12 vs 7) but this was not significant. | Medium |
| 23 | 17 | (Sydsjö, Sydsjö, Gunnervik, Bladh, & Josefsson, 2012)  Sweden  Quasi-experimental | **Intervention (n = 353)**  67% aged 25-34  95.1% married/cohabiting  45.2% skilled white collar workers  19.3% previous CS  17% previous instrumental delivery  **Control (n = 579)**  54.7% aged 25-34  93.9% married/cohabiting  44.6% skilled white collar workers  8.1% previous CS  7.3% previous instrumental delivery  P<.05 for age, previous CS and previous instrumental delivery | DSM-IV^4^ | **Individual counselling -**  including cognitive-behavioural and psychoeducational strategies. Women most often had one or two consultations at the unit for psychosocial obstetrics and gynecology; 47.3% of them met a specially trained midwife (1–7 sessions), 67.7% had consultations with an obstetrician (1–5 sessions) and 32.4% had consultations with a psychotherapist/psychologist (1–10 sessions). | SMC | No – women didn’t receive therapy because they didn’t have FOC | Obstetric outcomes  Pain relief | **Obstetric outcomes**  **Unassisted vaginal delivery**: intervention 50.9%; control: 74.7%  **Instrumental delivery**: Intervention: 6.8%; control 10.7%  **Emergency CS**: Intervention: 12.8%; control: 10.7%  **Elective CS:** intervention: 29.5%; control: 3.8% p<.001  **Pain relief**  **Epidural:** Intervention: 58.3%; control: 26.7%  <.001  **Pudental block**: Intervention: 3.9%; control: 0.8%  P<.004 | Medium |
| 24*  ▲ | 18 | (Jocelyn Toohill et al., 2014)  Australia  RCT | **Intervention (n = 170)**  Age M = 29 (SD = 5.9) Education of year 12 or less; 48.5%  Nulliparous: 57.4%  **Control (n = 169):**  Age M = 29.2 (SD = 4.98)  Educated year 12 or less: 36.1%  Nulliparous: 59.8% | WDEQ-A^1^ | **BELIEF Telephone psycho-education counselling:** The intervention aims to review women’s current expectations and feelings around FOC, support the expression of feelings, and provide a framework for women to identify and work through distressing elements of childbirth. | SMC | Yes | FOC^1^  Childbirth self-efficacy^8^  Depressive symptoms^15^ | **Fear of chidbirth**  **Intervention**:  Mean score change = 19.52 (SD = 18.59  Effect size 0.59  **Control:**  Mean score change = 9.28 (SD = 16.32).  A change in 20 points was chosen to indicate a clinically meaningful change. More women in intervention group showed improved childbirth fear scores (n = 48/98, 49%) compared to controls (n = 25/96, 26% p = 0.002). After adjusting for preintervention scores, a signiﬁcant difference resulted between groups on postintervention W-DEQ A scores for FOC, (F(1, 191) = 11.6, p = 0.001, partial eta squared = 0.06) with medium effect.  **Childbirth self-efficacy**  Mean change difference: 41.40, p = .002. Effect size: .46.  **Depressive symptoms**  Mean change difference: 5.6 (p =.09). | Low |
| 25*  ► | 18 | (J. Toohill, Callander, Gamble, Creedy, & Fenwick, 2017) | N = 184  Women who returned 6 week postnatal questionnaire |  |  |  |  | Obstetric outcomes  Cost-effectiveness | **Obstetric outcomes**  A larger proportion of women from the intervention group had a vaginal birth (66%) than women in the control group (58%). Women in the intervention group had 2.34 times the odds of having a vaginal birth than women in the control group (95% CI: 1.16–4.73, p = 0.014).  **Cost-effectiveness**  The mean ‘treatment’ cost for women receiving the intervention was AUS$72. The mean cost for health service use, excluding the cost of the intervention, was less in the intervention group (AUS$1193) than the control group (AUS$1236), however this difference was not significant (p = 0.78). |  |
| 26*  ► | 18 | (Fenwick et al., 2015) | N = 184  Women who returned 6 week postnatal questionnaire |  |  |  |  | CS preference  EPDS^15^  Satisfaction with birth  Delivery outcomes | **CS preference**  Less women in the intervention group compared to control said they would like a CS for next birth (16 vs 28, p = .04)  **EPDS**  Intervention: M = 6.2 (SD = 5) vs Control: M = 5.5 (SD = 4.7) (p = .3).  **Satisfaction with birth/Delivery outcomes**  No differences in assisted delivery, elective CS, emergency CS, induction of labour, narcotics in labour, epidural, preterm birth, breastfeeding at 6 weeks, satisfaction with birth mode. Women in the intervention arm were 8% less likely to have CS. |  |
| 27 | 18 | (Turkstra et al., 2017) |  |  |  |  |  | Health service use  EQ-5D-3L^20^ | The numbers of appointments with healthcare providers during the study period were similar in the intervention and control groups. The cost of healthcare use (excluding birth and special care nursery) during the study period was statistically significantly higher in the intervention group.  The probability that the intervention was more effective was 12%, while the probability that the intervention was less costly was 58%. |  |
|  |  | **Antenatal Education (k = 18 papers from 13 studies)** | | | | | | | | |
| **Paper no.** | **Study no.** | **Citation**  **Country**  **Design** | **Participant characteristics**  **Number in sample (n)** | **Measure used to determine FOC** | **Intervention** | **Control** | **Randomised?** | **Outcome measure** | **Outcome** | **Risk of bias** |
| 28 | 19 | (Bergström, Rudman, Waldenström, & Kieler, 2013)  Sweden  RCT | **Intervention (n = 39)**  Age: M = 32 (SD = 5.4); Married/cohabiting: 100%;  Secondary school education or less: 63% *  Planned pregnancy: 74%  Partner with from FOC: 33%  **Control (n = 44)**  Age: M = 33 (SD = 5.1); Married or cohabiting: 98%;  Secondary school education or less: 41%*  Planned pregnancy: 75%;  Partner with FOC: 28%  *p<.05 | WDEQ-A^1^ ≥ 60 | **Antenatal education:** Used the psychoprophylaxis model where the focus was on the man’s role as a coach during labour. Massage, breathing, emotional support and relaxation were taught.  **Intervention details:**  Four 2-hour sessions | SMC | Yes – internet based system | Childbirth experience^6^ | Men with FOC and in intervention group had lower risk of experiencing the childbirth as frightening (adjusted OR 0.30; 95% CI 0.10–0.95) and feeling unprepared for the birth (adjusted OR 0.20; 95% CI 0.05–0.86) | Low |
| 29*  ▼ | 20 | (El-Malky, El-Homosy, Ashour, & Shehada, 2018)  Quasi-experimental, pre-post  Egypt | **Intervention (n = 50)**  Age: M = 21.68 (SD = .33)  50% living in urban area  44% received higher education  46% housewife  **Control (n = 50)**  Age: M = 21.68 (SD = .33)?  54% living in urban area  38% received higher education  40% housewife | CAQ^8^ | **Antenatal education**  Class 1: definition of normal labour, preparation for labour, prenatal exercise, overview of phases and stages of labour (90 minutes)  Class 2: strategies to cope with fear through counselling and expression of feeling, breathing, relaxation (90 minutes)  Class 3: Discussion of issues, practicing of relaxation techniques (90 minutes) | No | SMC | Psychological wellbeing^24^  FOC^8^  Obstetric outcomes | **Psychological wellbeing:**  Pre-intervention: Intervention M = 31.46 (SD = 0.45)  Control: M = 31.30 (SD = 0.47)  Post-intervention  Intervention: M = 63.50 (SD = 0.41)  Control: M = 31.30 (SD = 0.47) p<.001  Difference significant between groups and for T1 & T2 for intervention group  **FOC**  Pre-intervention: Intervention M = 48.88 (SD = 0.71)  Control: M = 49.16 (SD = 0.59)  Post-intervention  Intervention: M = 25.50 (SD = 0.63)  Control: M = 31.30 (SD = 0.47) p<.001  Difference significant between groups and for T1 & T2 for intervention group  **Obstetric outcomes**  No significant differences between type of labour | High |
| 30*  ▲ | 21 | (Gökçe İsbir, İnci, Önal, & Yıldız, 2016)  Turkey  Quasi-experimental, pre-post | **Control (n = 46):**  Age M = 25.3  University graduates: 52.2%  **Intervention (n = 44):**  Age M = 26.8  University graduates: 70.5% | WDEQ-A^1^ | **Antenatal education:** based on Dick Read’s “Natural Labor” and Lamaze’s “Psychoprophylaxis hypnobirthing”  **Intervention details:**  Groups of 5-8 women  16 hours, made up of 4 sessions | No | SMC | FOC^1^  Childbirth self-efficacy^10^ | **FOC**:  T1: M = 66.8 (SD = 23.7)  T2: M = 30.4 (SD = 18.07  p <.001  **Childbirth self-efficacy:**  T1: M = 224.1(54.5)  T2: M = 297.9(SD = 17.8)  P<.001 | Medium |
| 31*  ▲ | 22 | (Karabulut, Coşkuner Potur, Doğan Merih, Cebeci Mutlu, & Demirci, 2016)  Turkey  Quasi-experimental, pre-post | **Intervention (n = 69)**:  Age M = 28.87 (SD = 4.54)  60.9% at university.  **Control (n = 123):**  Age M = 25.73 (SD = 5.35)  20.3% university  Education p <.05 | WDEQ-A^1^ | **Education intervention:** 5 weekly sessions covering the following-   1. Health in pregnancy 2. Birth and breathing exercises 3. Breastfeeding 4. Baby care 5. Postpartum period   **Intervention details:**  2 hour long sessions, groups of 6-10 couples | SMC | No – self volunteered to take part | FOC^1^ | **Intervention group:**  28 weeks  M = 53.25 (SD = 25.75)  Median = 54 at 28 weeks  Post intervention  M = 33.72 (SD = 24.33)  Median = 27  P<.001  **Control group**  28 weeks  M = 44.32 (SD = 5.00)  Median = 43 at 28 weeks  Post intervention  M = 41.97 (SD = 24.64)  Median = 41 post intervention  P = .02 | Medium |
| 32 | 23 | (Khedr & Eldeen, 2017)  Egypt  Pre-post | **Video group (n = 32)**  Age: 46.9% aged 24-29  46.9% completed secondary school or university  50% employed  **Lecture group (n = 32)**  Age: 50.0% aged 24-29  50.0% completed secondary school or university  37.5% employed  P<.05 | ‘Feelings of Fear and Security Associated with Pregnancy and Childbirth’^7^ | **Healthy instructions –** delivered via video or lecture.  **Video**: groups of 4-5 women, 30-minute video about normal delivery (definition, advantages, reduction of labour pain, preparation for delivery)  **Lecture** groups of 6-8 women who listened to a 30-minute lecture about normal delivery | None | n/a | Preference for mode of delivery  FOC^7^ | **Preference for mode of delivery**  Pre-intervention:  50% of women in **video** group wanted a CS; compared to 56.3% of women in **lecture** group (p>.05)  Post intervention  . 12.5% of women in **video** group wanted a CS compared to 34.4% (p =.04).  **FOC**  Pre intervention: 27% of women had low fear; 7% had moderate fear; 30% had high fear.  Post intervention: 47% had low fear; 6% moderate fear; 11% high fear  P<.001 | Medium |
| 33*  ▼ | 24 | (Kizilirmak & Başer, 2016)  Turkey  Quasi-experimental, pre-post | **Intervention group (n = 50):**  Age M = 22.2 (SD = 3.9)  38% graduated high school.  **Control group (n = 49)**  Age M = 22.5 (SD = 3.7)  44.9% graduated high school | WDEQ-A^1^ ≥ 85 | **Education intervention:** two sessions:   1. Labour: signs of labour, what to do, going to hospital 2. Delivery room, breathing, midwifery care, episiotomy | SMC | No – women admitted March and May controls, October and December were intervention | FOC^1^ | **Intervention:**  T1: M = 61.1  T2: M = 42  P <.001  60% felt confident during first stage of labour  **Control:**  Means increased by 2 points  42.3% felt confident during first stage of labour | Medium |
| 34 | 25 | (Kulkarni, Wright, & Kingdom, 2014)  Canada  Non -experimental | Nulliparous, singleton uncomplicated pregnancy. Majority (n = 40) born in Canada and were Caucasian.  Age M = 32.89, 23.3% postgraduate degree, 17.8% university degree  N = 73 | None | **E-intervention:** educational website. Further information available in paper’s appendix (systematic review author does not have access) | n/a | n/a | FOC^11^ | There was no significant change from baseline to final survey in the level of fear regarding vaginal delivery (P = 0.19).  Thirteen women changed from being either “neutral,” a “little fearful,” or “not fearful at all” regarding CS in the baseline survey to being “fearful” or “very fearful” (P = 0.05).  Participants had a significantly higher level of fear regarding CS compared to vaginal delivery in both the baseline survey (P = 0.03) and the final survey (P = 0.04). | Medium |
| 35 | 26 | (Ozdemir, Cilingir, Ilhan, Yildiz, & Ohanoglu, 2018)  Turkey  Block RCT | **Intervention (n = 50)**  Age M = 29.62 (SD = 4.40)  38% college education  92% married  54% housewife  **Control (n = 50)**  Age M = 30.12 (SD = 5.59)  34% college education  98% married  64% housewife  p>.05 | Request for CS | **Systematic birth preparation program group –** women received an education program which consisted of four 2-h sections. The content of the program included prenatal, postnatal, and neonatal care. Participants were informed about physical, mental changes, and possible emergencies in pregnancy. Nutritional support was provided. Physical exercise program supported by yoga and pilates was applied. Breathing techniques, hydrotherapy, aromatherapy, and reflexology were taught to control birth pain without medication. | SMC | Yes – block randomisation | Quality of life^24^  Depression^15^  Birth outcomes | **Quality of life**  Significant differences between groups in terms of physical, psychological and environmental quality of life.  **Depression**  No significant differences between groups  **Birth outcomes**  78% of women in intervention compared to 56% in control had a vaginal delivery (p = .03)  8% of women in intervention group requested a CS compared to 14% in control group (p = ,02) | Low |
| 36*  ▲ | 27 | (Serçekuş & Başkale, 2016)  Turkey  Quasi-experimental, pre-post | **Intervention:**  Women  Age M = 28.8 (SD = 2.2)  83.9% high school, 71% employed  Men  Age M = 31.8 (SD = 4.3)  83.9% university.  N = 31 couples  **Control**:  Women  Age M = 27.7 (SD = 4.5)  University 68.8  81.2% employed. Men  Age M = 29.9 (SD = 3.7)  university 75%  N = 32 couples | WDEQ-A^1^ | **Educational groups:** Content: nutrition, physiology and psychological changes and how to cope, mechanisms of labour, discussion of feelings of birth, dealing with FOC, coping with pain, physical and emotional postpartum changing, interaction, breastfeeding, new-born care  **Intervention details:** groups of 4-6 couples, once a week (120 minutes) 8 weeks. | SMC | No | FOC^1^  Childbirth self efficacy^10^  Attachment^12^ | **FOC**  **Intervention:**  T1: M = 60.7 (SD = 25.1),  T2: M = 37.9 (SD = 23.4).  **Control**:  T1:M = 54 (SD = 18.9),  T2:M = 59.9 (SD = 19.1). Significant difference between post-test scores for the groups (p <.0001)  **Childbirth self-efficacy**  **Intervention:**  T1: M = 219.3(SD = 25.1)  T2: M = 257.6(SD = 44.9).  **Control:**  T1: M = 222.7(SD = 37.4)  T2: M = 224.1(SD=37.8)  Significant difference between groups at time 2 (p = .002)  **Attachment**  No differences between groups | Medium |
| 37*  ▼ | 28 | (Taheri, Mazaheri, Khorsandi, Hassanzadeh, & Amiri, 2014)  Iran  Quasi-experimental, pre-post | **Intervention (n = 65):**  Age M = 26.72 (SD = 4.61)  **Control (n = 65)**  Age M = 27.72 (SD = 5.81) | Questionnaire “delivery fear” 14 questions using Likert score.^5^ | **Educational intervention:** Educational content was developed based on strategies of self-efficacy increase (success in performance, replace experience, verbal persuasion and physiological or emotional states).  **Intervention details:** three 60-90 minute sessions, 8-10 people per group | No intervention | Yes- stratified random sampling | FOC^5^  Childbirth self-efficacy^10^ | **FOC**  **Intervention:**  T1: M = 79.2 (SD = 12.1),  T2: M = 48.9 (SD = 5.8)  p <.001  **Control:**  T1: M = 78.6 (SD = 8.6)  T2: M = 78.8 (SD = 8.9)  P > .05  Significant difference between both groups after intervention p <.001  **Childbirth self-efficacy:**  **Intervention:**  T1: M = 12.8 (SD = 5.4)  T2: M = 29.5 (SD =3.8)  P<.001  **Control:**  T1: M = 12.9 (SD = 3.8)  T2: M = 12.9 (SD = 3.7)  Significant difference between both groups after intervention p <.001 | Low |
| 38 | 29 | (H. Rouhe et al., 2013)  Finland  RCT | **Intervention:**  Age M = 29.3 (SD = 4.6)  38.3% upper white collar workers  40.6% university education  97.2% cohabiting.  **Control**  Age M = 29.4 (SD = 4.8)  37.1% upper white collar workers  40.1% university education  91% married cohabiting  N = 330 | WDEQ-A^1^ ≥ 97 | **Psychoeducation and relaxation intervention:** led by a psychologist, every session began with relaxation with mindfulness exercise. Each session had a different focused topic:   1. Information about fear and anxiety 2. Information about FOC 3. Hospital routines, birth process, pain relief 4. Becoming a family 5. Becoming a mother, recognising signs of postnatal depression 6. Completing preparation for delivery and birth plan   **Intervention details:** each session was 90 minutes long. Six sessions before birth, and one 2-3 months after birth | Yes – in a proportion of 1:2 | SMC and advised to speak to maternity unit about their FOC:  -76 women seen at outpatient maternity clinics for FOC. Here they met an obstetrician M = 1.7 times, and/or a midwife M = 1.3 times.  -30 control women attended an advanced preparation class led by a specialised midwife 2–6 times (M = 2.5 times) | Birth experience^6^  Birth mode  Delivery satisfaction^13^ | **Birth experience**  Significant difference between the groups  **Intervention:** M = 63.0 (SD = 20) **Control:** M = 73.7 (SD =29)  F(199) = 1.1, p = 0.016 Cohen d = 0.35, small effect size)  **Birth mode:** Mothers in intervention group more likely to have spontaneous vaginal delivery  **Delivery satisfaction**  Those in intervention group were more likely to have a positive delivery experience (p=.04)  **Adherence**  131 women were randomised to the intervention, only 90 attended. 76% of women attended all 7 sessions on offer (Rouhe et al 2015b) | Low |
| 39 | 29 | (Salmela-Aro et al., 2012) |  |  |  |  |  | Childbirth preparedness^14^ | **Childbirth preparedness:**  **Intervention:**  Baseline: M = 3.67 (SD = 0.90)  1 month before birth: M = 4.36, SD = 0.97;  **Control:**  Baseline: M = 3.70 (SD = 0.92)  1 month before birth M = 4.18, (SD = 0.97)  The results showed that intervention increased a mother’s preparedness which, in turn, predicted an increase in positive parenting after childbirth among those in the intervention group. |  |
| 40*  ▲ | 29 | (Hanna Rouhe, Salmela-Aro, Toivanen, Tokola, Halmesmäki, Ryding, et al., 2015) |  |  |  |  |  | Birth mode  Delivery satisfaction  WDEQ-B  Depressive symtoms^15^  Post-traumatic stress symptoms^16^ | **Birth mode:** Women who attended group therapy had spontaneous vaginal birth signiﬁcantly more often than did specially treated control women [59 (65.6%) versus 50 (47.2%), P = 0.014]. CS by maternal request (FOC) was performed for 11 women in the intervention (12.2%) versus 24 women in the control group (22.6%) (P = 0.064).  **Delivery satisfaction:** More women in the intervention group had a very positive experience (DSS in the highest quartile) 36.1%, than women in the control group 22.8% (P = 0.04).  **WDEQ-B:** There was a significant difference between groups. Intervention M = 63 (SD = 32) Control M = 73.7 (SD = 29) indicating childbirth experience less fearful in intervention group.  **Postnatal adjustment:** There were significantly less severe postnatal depressive symptoms in the intervention group (EPDS M = 6.4 (SD = 5.4) compared to the control group (M = 8.0, SD= 5.9; p = 0.04, Cohen d = 0.28, small effect size).  No differences in terms of postnatal traumatic stress symptoms. |  |
| 41 | 29 | (Hanna Rouhe, Salmela-Aro, Toivanen, Tokola, Halmesmäki, & Saisto, 2015) |  |  |  |  |  | Satisfaction with life after birth^17^  Service use  Cost analysis | **Satisfaction with life after birth:** No significant differences were found.  **Service use:**  **Intervention group:** had a total of 526 visits to the intervention (M = 5.9 visits per woman), 122 visits to outpatient maternity clinics (M = 0.9 visits per woman) and 94 visits to the emergency maternity clinic (M = 0.7 per woman)  **Control group:** had 276 visits to outpatient maternity clinic (M = 1.2 per woman) and 150 emergency visits (M = 0.7 per woman).  **Cost analysis:**  Biggest differences in costs came from the intervention, which cost €34716 in total. However, when comparing the total cost per woman across the perinatal period the differences were minimal:  **Intervention: €**3.786 per woman  **Control: €**3,830 per woman |  |
| 42 | 29 | (Airo (Toivanen) et al., 2018) |  |  |  |  |  | PANAS^18^  FOC^19^  Personal Goals | **PANAS:** There was a significant decrease in negative emotions from the first session (Wald = 35.43, df = 6, p <.001). The treatment was the most effective regarding the emotions ‘scared’ (d = 0.84), ‘nervous’ (d = 0.66), and ‘jittery’ (d = 0.53).  There was an increase in positive emotion after women gave birth (Wald = 64.41, df = 6, p <.001).  **FOC:**  Pre: M = 7.60 (SD = 1.72)  Post: M = 4.56 (SD = 2.42) Wald = 230.43, df = 6, p < 0.001)  **Personal goals:**  The achievement of goals about the mother’s themselves x2(1) = 11.172, p < 0.001, and childbirth x2(1) = 20.045, p <.001 changed significantly during the intervention. |  |
| 43 | 29 | (Ryding et al., 2018) | Partners of women who participated in RCT.  **Intervention (n = 93)**  Age M = 32.7 (SD = 5.4);  33.3% university degree  **Control (n = 157)**  Age M = 31.1 (SD = 5.0);  45.2% university education | WDEQ-B^6^ ≥ 47 |  |  |  | Partners FOC^1^, symptoms of depression (EPDS)^15^ and PTSD (TES)^16^ | **Intervention:**  **WDEQ-A**  M = 34.2 (SD – 18.5)  **EPDS**  M = 3.5 (SD = 3.0)  **TES**  M = 18.2 (SD = 1.8)  **Control**  **WDEQ-A**  M = 35.2 (SD – 18.8)  **EPDS**  M = 3.9 (SD = 3.9)  **TES**  M = 19.5 (SD = 3.8) |  |
| 44*  ▲ | 30 | (Haapio, Kaunonen, Arffman, & Åstedt-Kurki, 2017)  Finland  RCT | **Intervention:**  48% aged 23-29;  57% married;  47% university education.  N = 338  **Control:**  47% aged 23-29,  58% cohabiting;  43% university degree  N = 321 | Data were collected using two subscales (objects of fears and manifestations of fears) from the questionnaire ‘Feelings of Fear and Security Associated with Pregnancy and Childbirth’^7^ | **Midwife-led intervention:**  Consisted of: information leaflet, a 2 hour childbirth class in the labour room with information about pain relief, push positions and delivery instruments. | SMC | Yes | FOC^7^  Wish to have a caesarean | **FOC**  The women’s everyday lives were less affected by fear in the intervention group in comparison with the control group (31% vs. 41%).  The situation in the intervention group also improved more often (14% vs. 8%) and worsened less often (12% vs. 18%) than in the control group. The difference between the groups was signiﬁcant [OR 0.64, 95% CL 0.44–0.94].  The intervention group had fewer moderate and severe childbirth-related fears than the control group (80% vs. 85%). Especially, severe childbirth-related fears were fewer in the intervention group compared to the control group (3% vs. 10%). Ordinal logistic regression model showed the differences were statistically signiﬁcant [OR 0.58, 95% CL 0.38–0.88].  **Wish to have caesarean**  No impact of intervention on wish to have CS (Intervention: 3% vs Control: 3.9%) | Low |
| 45*  ▼ | 31 | (Masoumi et al., 2016)  Iran  RCT | **Intervention (n = 80):**  Age M = 32.9 (SD = 7.9, range from 25 to 30 years)  **Control (n = 80):**  Age M = 32.9 (SD = 7.9, range from 25 to 30 years). | Chilbirth Attitude Questionnaire (CAQ)^8^ | **Midwife led training preparation:** classes on nutrition, exercise, labour, delivery, pain relief.  **Intervention details:** 10 – 15 people per group. Eight 2-hour sessions. | SMC | Yes – computer system | FOC^8^  Request for CS | **Intervention:**  T1: M = 53 (SD = 19.3)  T2: M = 51.7 (SD = 22.4  p =.24.  **Control:**  T1: M = 49.1, (SD = 21;  T2:M = 58.7 (SD = 21.7)  P = .01  There was an increase in the percentage of women with high fear from 43% to 56% in the control group.  **Request for CS**  There was a drop in women requesting CS in the intervention group, from 80% to 9.3%. It increased from 65.3 to 78.7% in control group (P<.001). | Low |
|  |  | **Enhanced midwifery care (k = 6 papers from 3 studies)** | | | | | | | | |
| **Paper No.** | **Study No.** | **Citation**  **Country**  **Design** | **Participant characteristics**  **Number in sample (n)** | **Measure used to determine FOC** | **Intervention** | **Control** | **Randomised?** | **Outcome measure** | **Outcome** | **Risk of bias** |
| 46 | 32 | (Hildingsson, Rubertsson, Karlström, & Haines, 2018)  Sweden  Feasibility | 60% aged over 30  100% cohabiting with partner  80% university education  N = 10 | FOBS^3^≥60 | **Caseload continuity midwifery model of care -** Women who consented to participate followed the standard visiting schedule for antenatal care. In addition, they were offered one extra visit in gestational week 25 and they were invited to join the psychoprophylaxis course free of charge. The women were assigned a named midwife whom they met during most antenatal visits. The midwife had a co-midwife that shared the on-call shifts, and the women had at least one visit to the co-midwife and both midwives were present at the standardized appointment in gestational week 36 when a summary of the pregnancy and a plan for the birth and the postpartum period, based on the women’s needs, were performed. The midwives were told by the research team to focus a lot on women’s childbirth fear during all visits and to discuss women’s feelings, causes of fear and coping strategies. | None | n/a | FOC  Satisfaction with treatment  Obstetric outcomes | **FOC**  Reduced over time from 68.60 (SD = 8.50) at baselined compared to 35.33 (SD = 23.30) 2 months after birth  70% felt their FOC alleviated  **Satisfaction**  60% of women were very satisfied with their treatment, 40% were satisfied  80% were overall very satisfied with intrapartum care  **Obstetric outcomes**  80% had a vaginal birth | High |
| 47*  ▼ | 32 | (Hildingsson, Karlström, Rubertsson, & Haines, 2019)  Sweden  Pilot study | 58.6% women aged over 32  98.6% living with partner  61.4% university education  N = 70 | FOBS^3^ | **Midwife continuity of care**  women with fear of birth, when possible, were offered continuity of care during labour and birth from the counselling midwife. | None | N/A | Adherence  Service use  Attitudes  Perceived effect of treatment | **Adherence**  34.3% had known midwife at the birth,  **Service use**  Women who had a known midwife during birth had more counselling visits (M = 3.20; SD = 1.97) compared to women without a known midwife (M = 2.06; SD = 1.10)  **Attitudes**  87.5% who had a known midwife at birth compared to 60.0% thought having a known midwife at the birth was important/very important  95.8% of women who had a known midwife compared to 71.7% were satisfied or very satisfied with midwifery counselling  **Perceived effect of treatment**  29.2% of women who had a known midwife compared to 4.5% stated that their childbirth fear disappeared | Medium |
| 48*  ▲ | 32 | (Hildingsson, Rubertsson, Karlström, & Haines, 2019) |  |  |  |  |  | Birth outcomes  Birth experience | **Birth outcomes**  No difference in pain relief used but women who had a known midwife reported a more positive pain experience (OR 1.5, 95% CI 1.09–2.13, Cohens d 0.72).  Spontaneous labour (known midwife = 45.8%; no known midwife: 56.5%; OR = 1.35; 95CI: 0.44-4.13)  Emergency CS (known midwife = 16.7%; no known midwife: 26.1%; OR = 0.47; 95CI: 0.13-1.75)  **Birth experience**  More women with known midwife rated their birth experience as positive/very positive (83.3%) compared to those without a known midwife (52.2%)  OR = 4.2 (95CI: 1.19-14.99) |  |
| 49 | 33 | (Lyberg & Severinsson, 2010a)  Norway  Qualitative, non-experimental | Women who received midwifery care for FOC  All aged between 25-37;  7 previous bad childbirth experience | Not clear | The intervention involved a team of four experienced midwives established to meet the needs of pregnant women. The goal of the approach was to ensure continuity, provide a feeling of security, illuminate individual needs and offer support throughout the pregnancy, childbirth and the perinatal period. | n/a | No | Qualitative analysis of FOC and experience of midwifery care | Women described their FOC, the reasons for the fear and being prepared for childbirth. They also described how being confirmed and treated with dignity by the midwife during pregnancy, childbirth and after the birth was a way to survive the feeling of being trapped and fear of the unpredictable birth.  The encounter(s) with the team midwife made women feel respected and dignified. They were very satisfied with the care provided and the fact that the midwives on the team were always accessible by phone. They praised the team members for this, for their ability to create spiritual and emotional experiences of the situation and for being present and available to meet their needs before, during and after the birth. The women considered the team midwives highly professional due totheir excellent collaboration within the team, their presence and their long experience of maternal care. They reported that the deep relationship with the midwife increased their self-esteem and personal development.. | Medium |
| 50 | 33 | (Lyberg & Severinsson, 2010b) |  |  |  |  |  | Qualitative analysis of midwives supervisory styles and leadership role as experienced by pregnant women and new mothers in the context of a FOC | The midwives supervisory style was stated to create trusting and caring relationships with the women, by understanding her individual situation and being familiar with her needs and wishes. Midwives were described as being sensitive to individual needs and wishes, acting in accordance with individual needs and providing hope and confirmation.  Midwives were also seen as demonstrating a problem-solving capacity through being understanding and explaining the reasons for a FOC  Midwives also showed a willingness, preparedness and courage to support women.  The midwives leadership was described as competent, by assuming the responsibility for managing the process, creating a conducive work climate and empowering women. |  |
| 51 | 34 | (Sydsjo et al., 2014)  Sweden  Quasi-experimental | **Intervention (n = 14)**  Age: M = 31.6 (SD = 5.7);  90.9% married cohabiting;  72.7% employed  Women had FOC  **Control (n = 28)**  Age: M = 29.2 (SD = 3.4);  100% married cohabiting;  84.6% permanently employed.  Women did not have FOC | DSM-IV^4^ | **Visiting delivery suite –**  1. The woman was invited to the delivery ward for a first meeting with the selected midwife.  2. At the first meeting the delivery ward was presented and the delivery rooms were shown. All equipment was explained and tested if this was asked for. An external and sometimes a vaginal examination were offered as well as a CTG (cardiotocogram). The examinations and the foetal surveillance were offered in order to expose the women to these routines.  3. A second visit with the selected midwife at the delivery ward was offered if the woman wished.  4. The woman was encouraged to contact the midwife by telephone or schedule additional visits to the delivery ward for further support or information if needed.  5. If a birth plan /contract had not been established the midwife and the woman wrote an appropriate document.  6. Information was given to each woman that the two assigned midwives would do their utmost to attend and assist the woman’s childbirth;  7. Individual support was continuously offered to the two assigned midwives by the obstetrician in charge at the psychosocial unit | SMC | No – if women didn’t have FOC they did not receive an intervention | Obstetric outcomes | **Duration of labour for multiparous women**: Intervention M = 233.0 (SD=123.1); Control: M = 366.9 (SD = 157.0), p = .047 (no difference for primiparous women)  **Emergency CS:** Intervention 27.3%; Control: 3.8% p =.07  **High level of anxiety suffered during childbirth**: Intervention 80%; Control: 19.2% p =.001  No significant differences for pain relief use (p = .203-.540), effectiveness of pain relief (p =1); experiences of childbirth (p = 1), outcome of childbirth compared to expectations (p = .21) | Medium |
|  |  | **Alternative/other types of interventions (k = 13 papers from 12 studies)** | | | | | | | | |
| **Paper no.** | **Study no.** | **Citation**  **Country**  **Design** | **Participant characteristics**  **Number in sample (n)** | **Measure used to determine FOC** | **Intervention** | **Control** | **Randomised?** | **Outcome measure** | **Outcome** | **Risk of bias** |
| 52 | 35 | (Baleghi, Akerdi, & Pasha, 2016)  Iran  Quasi-experimental, pre-post | Low risk pregnant women.  **Intervention group (n= 57):**  Age M = 25.8  **Control group (n = 55):**  Age M = 26.4  N=112 | "Standardised questionnaire with 20 questions about the causes of childbirth fear (in the area of pain, liability, injury, prematurity)" answered on a 5-point Likert scale. | **Relaxation classes:** Techniques taught for relaxation during childbirth.  **Session details:**  8 sessions, 90 minutes | Routine care | No – categorisation not explained | FOC Questionnaire (Lower scores indicate less fear)  Delivery outcomes | **FOC**  **Control group:**  T1: M = 58.5, SD = 14.2  T2: M = 58.7, SD = 14.9  **Intervention group:**  T1: M = 48.5 SD = 3.9  T2: M = 40.5, SD = 12.4 (p=0.000).  Significant difference between both groups at T2: (*p* = 0.000)  **Delivery outcomes**  Intervention:  49% vaginal delivery  **Control:**  32.7% vaginal delivery  P = .033 | Medium |
| 53*  ▲ | 36 | (Bulez, Ceber Turfan, & Sogukpinar, 2019)  Turkey  Quasi-experimental, pre-post | **Intervention (n = 30)**  Age: M = 25.74 (SD = 5.16, Range: 17-39); 20.87 weeks gestation; 35% secondary education; 35.5% previous birth  **Control (n = 30)**  Age: M = 28.7 (SD = 5.42, Range: 21-37); 24.1 weeks gestation; 20% secondary school graduate; 70% given birth previously | WDEQ-A^1^ | **Hypnobirthing education -** given one hour a week totalling 4 hours in 4 weeks.  Hypnobirthing focuses on teaching self-hypnosis,  breathing slowly, letting oneself go and the art of  enjoying labour calmly and serenely, discovering the  method of delivery without stress, forming a positive  expectation, trust and faith in the spontaneity of  labour. | Antenatal education | No | FOC^1^ | **Intervention:**  Pre: M = 79.47 (SD = 11.58)  Post: M = 67.10 (SD = 11.00)  t =5.21, p <.001.  **Control**  Pre: M = 84.60 (SD = 16.71) | High |
| 54 | 37 | (Fisher, Hauck, Bayes, & Byrne, 2012)  USA  Single arm pilot – qualitative data | Nulliparous women, singleton pregnancy.  Age: M = 30.1  89% married/cohabiting  56% had undergraduate degree  N = 18 | WDEQ-A^1^ | **Mindfulness Intervention:** Intergration of mindfulness and birth education. Targets self-efficacy, communication and decision making  **Intervention details:**  8 sessions  2.5 hours long | No | N/A | Qualitative data | Women felt empowered, and a sense of community. Women reported feelings of awakening existing potential, being an informed active participant in the birth process, ability to stay calm and work as a team, challenge of applying mindfulness beyond birth; being in a community of likeminded parents. | Medium |
| 55 | 37 | (Byrne, Hauck, Fisher, Bayes, & Schutze, 2014) |  |  |  |  |  | Childbirth fear^1^  Childbirth self- efficacy^8^  Adherence | **Childbirth fear**  T1: M = 61.42  T2: M = 38.93  P <.001  **Childbirth self-efficacy:**  T1: M = 171.69  T2:M = 224.54  P<.001  **Adherence:** all 18 participants completed programme, but meditation homework adherence was low (average 3.6 sessions per week). |  |
| 56 | 38 | (Hunter et al., 2011)  USA  Quasi-experimental | Actively involved in military. 20 pregnant women and 10 partners.  **Intervention (N = 20)**  15 female; 70% white; 25% black, 90% married or cohabiting; 35% completed some college;  **Control (N = 10)**  7 females; 50% black; 33% Asian; 90% married or cohabiting; 44% completed some college | WDEQ-A^1^ | **Mantram repetition intervention:**  Participants were given a mantram handbook, which was a list of common mantrams and were asked to choose one. For homework assignments participants were taught to a) mentally repeat the chosen mantram and b) ignore any other thoughts that intrude. | Yes – table of random numbers | **Control group:** focus on healthy living and responsible decision making | FOC^1^  Adherence to intervention | **FOC**  No means reported. There was a decrease in fear in the intervention group: Baseline to 6 weeks postdelivery (Z = -1.78, p = .07); this became significant when missing values were replaced (z = -2.03, p .05)  **Adherence:**  Mothers reported using mantram 5 days per week, five sessions per day. Median practising was 3.5 mantram sessions per day. | Low |
| 57*  ▼ | 39 | (Guder, Yalvac, & Vural, 2018)  Cyprus  Quasi-experimental, pre-post | **Intervention (n = 54)**  Age: M = 28.05 (SD = 2.91)  61.1% undergraduate degree  100% married  **Control (n = 54)**  Age: M = 28.00 (SD = 3.69)  59.3% undergraduate degree  100% married  p>.05 | WDEQ-A^1^ | **Pilates assisted childbirth preparation training**  8 weeks long  During the first phase of the study, this program was executed twice a week for 2 h each day comprising 1 h of theoretical education, 45 min of pregnancy Pilates session, and 15 min of  breathing exercises in the first 4 weeks. The final 4 weeks of this training program consisted of only pregnancy Pilates and breathing exercises.  . | SMC | No | FOC^1^  Obstetric outcomes | **FOC**  Pre-training  Intervention: M = 57.44 (SD = 23.24)  40.7% moderate FOC  25.9% clinical FOC  Control: M = 57.35 (SD = 19.80)  42.6% moderate FOC  31.5% clinical FOC  p>.05  Post-training  Intervention: M = 33.88 (SD = 22.34)  29.6% moderate FOC  7.4% clinical FOC  Control: M = 60.72 (SD = 21.21)  31.5% moderate FOC  48.1% clinical FOC  p<.001  **Obstetric outcomes**  Out of the women who had given birth via caesarean, 8.1% of women in intervention had planned a CS compared to 25.5% p=.008 | Medium |
| 58*  ▲ | 40 | (Guszkowska, 2014)  Poland  Quasi-experimental, pre-post | Age: M = 29.05  80.7% married  100% higher education  N = 109  N = 62 in intervention group; 47 in control. Demographics not reported for groups separately. | The Fear of Childbirth Scale. This scale has 14 items addressing feelings relating to childbirth, both fears (7 items, e.g. I am worried that something bad will happen to me or my baby during childbirth) and their absence (7 items, e.g. I feel safe when I imagine the childbirth). A four-point response format was used (from 1 = definitely disagree to 4 = definitely agree). | **Exercise intervention:**  Yoga, pilates, body ball, relaxation and breathing exercises  **Intervention details:**  Eight 50-minute long sessions | SMC | No -participants were asked if they wanted to do the exercise classes, those that said no were control | FOC  Locus of control for labour pain | **FOC**  **Intervention:**  T1: M = 32.89  T2: M = 31.06  **Control:**  T1: M = 30.57  T2: M = 30.82  **Locus of labour pain control internal**  **Intervention**: M = 11.94  **Control**: M = 12.63  (p >.05) | Medium |
| 59 | 41 | (Narita, Shinohara, & Kodama, 2018)  Japan  Quasi-experimental | **Intervention (n = 18)**  Age: M = 32.4 (SD = 3.8); 65% nulliparous; WDEQ M = 79.5 (SD = 8.5); PSQI: m = 5.2 (sd = 2.9); Fatigue VAS M = 45 (SD = 21.4)  **Control (n = 22)**  Age M = 32.7 (SD = 5); WDEQ M = 82 (sd = 14.1); PSQI M = 5.3 (SD = 2.6); Fatigue VAS M = 46.4 (SD = 19). | WDEQ-A^1^ ≥ 66 | **Heart rate variability (HRV) Biofeedback** - Women who provided written consent for HRV biofeedback were given a portable HRV biofeedback device which records real-time heart rate information via the finger.  The pregnant women first practiced HRV biofeedback during outpatient consultation on the day of recruitment. They were given the devices after confirming that they could use them easily at home. Each participant was told to practice HRV biofeedback consistently every night before sleeping for 3–4 weeks and to record how well they practiced the method on a recording form. | SMC | No – women didn’t agree to take part in HRV biofeedback | FOC (WDEQ)^1^ Pittsburgh Sleep Quality Index^22^ | There were significant time-dependent (from 32-34 weeks to 36-67 weeks) variations in the W-DEQ (p < 0.001) and PSQI scores (p = 0.012) and a significant time × group interaction in the W-DEQ scores (p = 0.002).  Time-dependent reductions in the W-DEQ scores were significant only in the biofeedback group (paired t-test, p < 0.001), whereas time-dependent increases in the PSQI scores were significant only in the control group (p < 0.001). | Medium |
| 60 | 42 | (Navaee & Abedian, 2015)  Iran  Quasi-experimental | Age M = 24 (SD = 4)  51.4% high school education  58% housewives  N = 35 role play  N = 32 lecture group | Harman Childbirth Attitude  Questionnaire (CAQ)^8^ | **Role play:** around advantages and disadvantages of vaginal delivery. Two facilitators played three scenarios, participants would also get involved in role play. 90 minute session. | **Lecture group:** 90 minute Power-point session | No – cluster sampling | FOC^8^ | **Lecture group**:  T1: M = 39, SD = 7  T2: M = 36.3, SD = 8  p = .047  **Role play group:**  T1: M = 35.6 (SD = 8.5)  T2: M = 30, (SD = 8.6)  p = .001. | Medium |
| 61*  ▲ | 43 | (Pour-Edalati, Moghadam, Shahesmaeili, & Salehi-Nejad, 2019)  Iran  Pre-post test | **Intervention**  Age: M = 30.55 (SD = 3.37)  55% housewives  40% Bachelor’s degree  N = 20  **Control**  Age: M = 30.90 (SD = 3.71)  47.6% housewives  47.6% Bachelor’s degree  N = 21 | CAQ^8^ | **Mindfulness based stress reduction**  8 90 minute sessions. Each session focused on different topics: intuitive guidance, facing obstacles, breathing mindfulness, staying in the present moment, permission to attend, thoughts are not facts, effective self-care, acceptance and change. | Not clear | No | FOC^8^ | There was a significant reduction in FOC scores in the intervention group (Pre: M = 37.85; SD = 5.59, Post: M = 36.35; SD = 5.40, p <.001)  No difference in control arm (p = .46) | Medium |
| 62 | 44 | (Sezen & Ünsalver, 2019)  Turkey  Quasi-experimental, pre-post | **Intervention (n = 15)**  Age M = 28 (SD = 4.9)  73.3% completed university  53.3% house-wife  **Control (n = 15)**  Age M = 26.3 (SD = 4.8)  73.3% completed university  53.3% housewife  Intervention group were older. | WDEQ-A^1^ | **Art therapy –** carried out in groups. Six sessions lasting 130 minutes each.  All sessions involved listening to music and singing together. Sessions covered mask making, drawing, mandala-making, puppet making, taking photographs and collage making. At the beginning of each session there was 40 minutes of psychoeducational information provided by therapist. | SMC | No | FOC^1^  Anxiety^25^  Depression^26^ | **FOC**  Pre intervention  Intervention Median = 51  Control median = 56  Post-intervention  Intervention median = 28  Control median = 55  P<.001  **Anxiety**  Pre intervention  Intervention Median = 24  Control median = 22  Post-intervention  Intervention median = 8  Control median = 23  P<.001  **Depression**  Pre intervention  Intervention Median = 23  Control median = 23  Post-intervention  Intervention median = 7  Control median = 21  P<.001 | Medium |
| 63*  ► | 45 | (Terhi Saisto, Toivanen, Salmela-Aro, & Halmesmäki, 2006)  Finland  Quasi-experimental | **Intervention:**  Age M = 31.49 (SD 4.8 years  93% permanent relationship  76% were working 40% had a university degree.  N = 102  **Control:**  Age M = 31.1 (SD = 4.8).  N = 85 | Referred for consultation because of fear of vaginal delivery and request for a cesarean. Women in experience group had a M = 6.9 (SD = 2.0) on Areskog questionnaire^2^. Control M = 6.0 (SD = 1.6). | **Group psychodynamic therapy:** five weekly themes –   1. Effects of relaxation 2. Stages of delivery 3. Pain relief 4. Parenthood 5. Wishes and written notes addressed to midwife   **Intervention details:**  5 weekly sessions, 120 minutes long. | **Conventional treatment:** met an obstetrician twice for 20-40 minutes to discuss fear and mode of delivery | No – asked if they wanted to take part in group sessions. | Birth mode  Women’s views of the intervention | **Birth mode:** Caesareans due to FOC were performed on 13 subjects (12.7%) in the experimental compared to 19 in the comparison group (22.4%) (Fisher’s exact test p = 0.040.  **Women’s views of intervention:** Women rated intervention as M = 8.5 in terms of benefit to themselves (0 = group had no benefit, 10 = group had maximal benefit) | Medium |
| 64 | 46 | (Wahlbeck, Kvist, & Landgren, 2018)  Sweden  Qualitative data from an RCT | Age: M = 32 (range = 27-41)  11/19 completed high upper secondary school  10/19 were nulliparous  N = 19 | WDEQ-A^1^ | **Art therapy + counselling -**  During pregnancy, participants had received five sessions of art therapy, either one-to-one or in a group setting as an adjunct to usual care (counselling). | Counselling | Yes (but this study only looks at women who received art therapy) | Qualitative | **Gaining hope and self-confidence:**  women described shedding their fear during art therapy.  The women experienced painting as an important tool to promote inner healing processes in the treatment of their FOC.  Art therapy was perceived to take up a lot of the women’s time in everyday life but also to give a time frame in which to identify and process the fear.  Painting and drawing made it possible for the women to open up their deeply hidden feelings, related to having suffered a traumatic birth or other trauma connected to the fear of childbirth. The women expressed that it made them able to connect with the fear and to release it. | Low |
|  |  | **Interventions during labour (k = 2)** | | | | | | | | |
| **Paper no.** | **Study no.** | **Citation**  **Country**  **Design** | **Participant characteristics**  **Number in sample (n)** | **Measure used to determine FOC** | **Intervention** | **Control** | **Randomised?** | **Outcome measure** | **Outcome** | **Risk of bias** |
| 66 | 47 | (Irmak Vural & Aslan, 2019)  Turkey  RCT | **Intervention 1 - EFT-G (n = 35)**:  Age M = 27.29 (SD = 3.97);  62.9% university educated;  91.4% first time pregnancy.  **Intervention 2 - BA-G (n = 35):**  Age M = 27.51 (SD =4.65);  68.6% university educated;  88.6% first pregnancy  **Control (n = 35)**  Age M = 27.36 (SD = 4.19);  78% university educated;  92% first pregnancy.  No differences between groups | WDEQ-A^1^ | **Emotional Freedom Technique (EFT):** is a psychophysiological intervention that combines elements of cognitive behavioral therapy (CBT), exposure therapy and somatic stimulation using acupressure points (i.e. tapping). Three EFT sessions were performed when the woman had no pain during 0-3cm dilation; 4-7cm dilation and 8-10cm dilation.  A total of nine EFT sessions were conducted with each pregnant woman**.**  **Breathing awareness** This group was exposed to abdominal breathing awareness. The researcher explained this intervention by demonstrating it for 10 min in the pregnant women's room in the latent (0-3cm dilation) phase of labour. The women were asked to lie in a comfortable position and breathe in and out calmly and smoothly at the beginning of each contraction. | SMC | Yes – women selected a number from a bowl, and an online system determined allocation | Childbirth experience^6^  Pain and distress during labour | **Childbirth experience**  EFT M = 59.17 (SD = 18.52);  BA: M = 59.57 (SD = 18.76)  Control: M = 71.74 (SD = 13.74)  p<.001 (lower score = better birth experience).  **Pain and distress**  **0-3cm cervical dilation**  EFT: M = 1.91 (SD = 1.52)  BA: M = 2.80 (SD = 1.81)  P = .055  **4-7cm cervical dilation**  EFT: M = 2.51 (SD = 1.40)  BA: M = 4.00 (SD = 1.48), p <.001  **8-10cm cervical dilation**  EFT: M = 3.86 (SD = 1.44)  BA: M = 5.94 (SD = 1.78)  P <.001 | Low |
| 66 | 48 | (Phumdoung, Youngvanichsate, & Wongmuneeworn, 2011)  Thailand  RCT | Demographics not presented by group, but by parity:  **Primiparous**  Age M – 22.95 (SD = 4.91)  63.1% Buddhist  27.7% had 9 years of education  **Multiparous**  Age M = 26.59 (SD = 4.59)  72.4% Buddhist  32.9% had 9 years of education  70 women participated in the music group; and 75 in the control group. | 100mm visual analogue scale, left phrase “no fear of childbirth at all”, right phrase “the most fear of childbirth” | **Marching songs and cheerful music -**  Marching songs were chosen as it was hypothesised that it would stimulate labour 1) the U.S.national anthem, “The Star-Spangled Banner” (114 beats/min); 2) the “Marines’ Hymn” (106 beats/min); 3) “The U.S. Field Artillery March” (119 beats/min); and 4) “The Stars and Stripes Forever” (113 beats/min).  This was followed by cheerful instrumental music hypothesised to help relax the women, from “The Four Seasons” and other works by Vivaldi: 1) Spring Concerto No. 1 in E major (109 beats /min); 2) Summer Concerto No. 2 in G minor (107 beats/min); 3) Oboe Concerto in C major (100 beats/min); and 4) Piano Concerto in C Major (78 beats/min).  The duration of the music was 30 minutes and had to repeat after using for 30 minutes if the women were still in second-stage labour. | Not stated | Yes – Random block design | FOC  Power  Length of labour  Sense of control | **FOC**  Intervention: M = 48.31 (SD = 27.04)  Control: M = 58.69 (SD = 31.70)  t = 1.419, p = .161  **Sense of power**  Intervention: M = 62.78 (SD = 21.48)  Control: M = 59.33 (SD = 24.00)  t = 0.610, p = .544  **Self-control**  Intervention: M = 55.25 (SD = 21.19)  Control: M = 54.87 (SD = 22.52)  t = 0.068, p = .946  **Duration of second-stage labour**  Intervention: M = 25.90 (SD = 16.24)  Control: M = 28.63 (SD = 21.25)  t = 0.581, p = .564 | Medium |

**Note:** ^*^Included in the meta-analysis; ▲ = FOC measure used in meta-analysis; ► = Birth outcome measure used in meta-analysis; ▼ = Both FOC and birth outcome used in meta-analysis.

1 = Wijma Delivery Expectancy Questionnaire (Wijma, Wijma, & Zar, 1998); 2 = Areskog’s questions (Areskog, Kjessler, & Uddenberg, 1982); 3 = Fear of Birth Scale (Ternström, Hildingsson, Haines, & Rubertsson, 2016); 4 = Diagnostic and Statistical Manual of Mental Disorders 4^th^ edition (American Psychiatric Association, 2000); 5 = Efficacy of delivery questionnaire (Khorsandi et al., 2008); 6 = Wijma Delivery Experience Questionnaire (Wijma et al., 1998); 7 = Feelings of Fear and Security Associated with Pregnancy and Childbirth (Melender, 2002);8 = Childbirth Attitudes Questionnaire and Childbirth Self Efficacy Questionnaire (Lowe, 2000); 9 = birth related concerns (Nurmi, 1991); 10 = Childbirth self-efficacy (Ip, Tang, & Goggins, 2009); 11 = the authors used their own measure by provide no details; 12 = Mother-infant attachment (Müller, 1994); 13 = Delivery Satisfaction Scale (T. Saisto, Salmela-Aro, Nurmi, & Halmesm??ki, 2001); 14 = Preparedness for childbirth scale, developed by the authors. 14 questions about self-efficacy (e.g. How well do you think you can keep active during childbirth?), social support (e.g. How easily do you think you can ask for help and advice during the childbirth?) and dealing with possible setbacks (e.g. If there is a problem during childbirth, I trust that I will receive help) on a seven-point scale ranging from 1 = not at all to 7 = a lot; 15 = Edinburgh Postnatal Depression Scale (Cox, Holden, & Sagovsky, 1987); 16 = Traumatic Events Scale (Wijma, Soderquist, Carlsson, & Wijma, 2000); 17 = Satisfaction with life scale (Diener, Emmons, Larsem, & Griffin, 1985); 18 = Positive and Negative Affect Scale (Crawford & Henry, 2004); 19 = Visual Analogue Scale (1 = no fear; 10 = a lot of fear) (H. Rouhe et al., 2013); 20 = Euro Quality of Life – 5 dimensions (Rabin, Gudex, Selai, & Herdman, 2014); 21 = Impact of Events Scale (Horowitz, Wilner, & Alvarez, 1979); 22 = Pittsburgh Sleep Quality Scale (Buysse, Reynolds, Monk, Berman, & Kupfer, 1989); 23 = Pictorial Representation of Attachment Measure (PRAM) (van Bakel, Maas, Vreeswijk, & Vingerhoets, 2013); 24 Psychological wellbeing measure made by authors after extended review of literature and revised by five experts in the field of psychiatric nursing and psychiatric medicine to test its content validity. It consists of 29-items questionnaire with a response rate of 1-3 (score 3 for strongly agree, score 2 for moderately agree, score 1 for slight agree) with higher scores representing higher psychological wellbeing. The maximum possible total score was: 3 × 29 = 87 and the minimum possible total score was: 29 ×1=29; 24 = WHOQOL-BREF was developed by the World Health Organization as a brief version of the WHOQOL-100 instrument, contained 26 questions (World Health Organization, 1996); 25 = Beck Anxiety Inventory (Beck & Steer, 1990); 26 = Beck Depression Inventory (Beck, Steer, & Brown, 1996)

**Table S3. Methodological quality of studies**

| **Author and year** | **Q.1** |  | **Q.2** |  | **Q.3** |  | **Q.4** |  | **Q.5** |  | **Q.6** |  | **Q.7** |  | **Total** | **%** | **Label** |
| --- | --- | --- | --- | --- | --- | --- | --- | --- | --- | --- | --- | --- | --- | --- | --- | --- | --- |
| (Ahmadi et al., 2018) | No | 0 | Yes | 0 | No | 0 | Unknown | 0 | Yes | 1 | Yes | 1 |  | 1 | 3 | 60 | Medium |
| (Airo (Toivanen) et al., 2018; H. Rouhe et al., 2013; Hanna Rouhe, Salmela-Aro, Toivanen, Tokola, Halmesmäki, Ryding, et al., 2015; Hanna Rouhe, Salmela-Aro, Toivanen, Tokola, Halmesmäki, & Saisto, 2015; Ryding et al., 2018; Salmela-Aro et al., 2012) | Yes | 1 | Yes | 0 | No | 0 | Unknown | 0 | Yes | 1 | Yes | 1 |  | 1 | 4 | 80 | Low |
| (Andaroon et al., 2017) | Yes | 1 | Yes | 0 | No | 0 | Unknown | 0 | Yes | 1 | Yes | 1 |  | 1 | 4 | 80 | Low |
| (Baleghi et al., 2016) | No | 0 | Yes | 0 | No | 0 | Unknown | 0 | Yes | 1 | Yes | 1 | Participant characteristics are minimum | 0 | 2 | 40 | Medium |
| (Baylis et al., 2019; Hildingsson & Rubertsson, 2019; Larsson et al., 2019, 2017; Rondung et al., 2018) | Yes | 1 | Yes | 0 | No | 0 | Unknown | 0 | Yes | 1 | Yes | 1 |  | 1 | 4 | 80 | Low |
| (Bergström et al., 2013) | Yes | 1 | Yes | 0 | No | 0 | Unknown | 0 | Yes | 1 | Yes | 1 |  | 0 | 3 | 60 | Medium |
| (Bulez et al., 2019) | No | 0 | Yes | 0 | No | 0 | Unknown | 0 | No | 0 | No - needed to report WDEQ scores after control | 0 |  | 1 | 1 | 20 | High |
| (Byrne et al., 2014; Fisher et al., 2012) | No | 0 | Yes | 0 | No | 0 | Unknown | 0 | Yes | 1 | Yes | 1 |  | 1 | 3 | 60 | Medium |
| (El-Malky et al., 2018) | No | 0 | Yes | 0 | No | 0 | Unknown | 0 | No | 0 | No | 0 |  |  | 0 | 0 | High |
| (Fenwick et al., 2015; J. Toohill et al., 2017; Jocelyn Toohill et al., 2014; Turkstra et al., 2017) | Yes | 1 | Yes | 0 | No | 0 | No | 0 | Yes | 1 | Yes | 1 |  | 1 | 4 | 80 | Low |
| (Gökçe İsbir et al., 2016) | No | 0 | Yes | 0 | No | 0 | Unknown | 0 | Yes | 1 | Yes | 1 |  | 1 | 3 | 60 | Medium |
| (Guder et al., 2018) | No | 0 | Yes | 0 | No | 0 | Unknown | 0 | Yes | 1 | Yes | 1 |  | 1 | 3 | 60 | Medium |
| (Guszkowska, 2014) | No | 0 | Yes | 0 | No | 0 | Unknown | 0 | Yes | 1 | Yes | 1 | Reporting of outcomes difficult to follow in some places | 0 | 2 | 40 | Medium |
| (Haapio et al., 2017) | Yes | 1 | Yes | 0 | No | 0 | Unknown | 0 | Yes | 1 | Yes | 1 |  | 1 | 4 | 80 | Low |
| (Halvorsen et al., 2010) | No | 0 | Yes | 0 | No | 0 | Unknown | 0 | Yes | 1 | Yes | 1 |  | 1 | 3 | 60 | Medium |
| (Henriksen et al., 2018) | No | 0 | Yes | 0 | No | 0 | Unknown | 0 | No | 0 | No – differences in birth outcomes were not reported | 0 |  | 0 | 0 | 0 | High |
| (Hildingsson & Rubertsson, 2019; Hildingsson et al., 2018; Hildingsson, Rubertsson, et al., 2019) | Yes | 1 | Yes | 0 | No | 0 | Unknown | 0 | Yes | 1 | Yes | 1 |  | 1 | 4 | 80 | Low |
| (Hunter et al., 2011) | Yes | 1 | Yes | 0 | No | 0 | Unknown | 0 | Yes | 1 | Yes | 1 |  | 1 | 4 | 80 | Low |
| (Irmak Vural & Aslan, 2019) | Yes | 1 | Yes | 0 | No | 0 | Yes | 1 | Yes | 1 | Yes | 1 |  | 1 | 5 | 100 | Low |
| (Karabulut et al., 2016) | No | 0 | Yes | 0 | No | 0 | Unknown | 0 | Yes | 1 | Yes | 1 | There is a higher education level in intervention group | 0 | 2 | 40 | Medium |
| (Khedr & Eldeen, 2017) | Yes | 1 | Yes | 0 | No | 0 | No | 0 | Yes | 1 | Yes | 1 |  | 1 | 4 | 80 | Low |
| (Kizilirmak & Başer, 2016) | No | 0 | Yes | 0 | No | 0 | Unknown | 0 | Yes | 1 | Yes | 1 |  | 1 | 3 | 60 | Medium |
| (Klabbers et al., 2018, 2019) | Yes | 1 | Yes | 0 | No | 0 | Unknown | 0 | Yes | 1 | Yes | 1 | Potential differences in education and parity across groups but not clear if these are significant. Slightly lower WDEQ in control group (not clear if significant). Some of the same transferred to other treatments so potentially self-selecting sample | 0 | 3 | 60 | Medium |
| (Kordi et al., 2017) | Yes | 1 | Yes | 0 | No | 0 | Unknown | 0 | Yes | 1 | Yes | 1 |  | 1 | 4 | 80 | Low |
| (Kulkarni et al., 2014) | No | 0 | Yes | 0 | No | 0 | Unknown | 0 | Yes | 1 | Yes | 1 | Did not report how FOC was identified | 0 | 2 | 40 | Medium |
| (Larsson et al., 2015) | No | 0 | Yes | 0 | No | 0 | Unknown | 0 | Yes | 1 | Yes | 1 |  | 1 | 3 | 60 | Medium |
| (Lyberg & Severinsson, 2010a, 2010b) | No | 0 | Yes | 0 | No | 0 | Yes | 1 | Yes | 1 | Yes | 1 | Did not report how FOC was identified | 0 | 3 | 60 | Medium |
| (Masoumi et al., 2016) | Yes | 1 | Yes | 0 | No | 0 | Unknown | 0 | Yes | 1 | Yes | 1 |  | 1 | 4 | 80 | Low |
| (Narita et al., 2018) | No | 0 | Yes | 0 | No | 0 | Unknown | 0 | Yes | 1 | No - no means between groups | 0 |  | 1 | 2 | 40 | Medium |
| (Navaee & Abedian, 2015) | No | 0 | Yes | 0 | No | 0 | Unknown | 0 | Yes | 1 | Yes | 1 | Did not report demographics for each group separately | 0 | 2 | 40 | Medium |
| (Nerum et al., 2006) | No | 0 | Yes | 0 | No | 0 | Unknown | 0 | Yes | 1 | No – FOC score not reported | 0 |  | 1 | 2 | 40 | Medium |
| (K. Nieminen et al., 2015; Katri Nieminen et al., 2016) | No | 0 | Yes | 0 | No | 0 | Unknown | 0 | Yes | 1 | Yes | 1 |  | 1 | 3 | 60 | Medium |
| (Ozdemir et al., 2018) | Yes | 1 | Yes | 0 | No | 0 | Unknown | 0 | Yes | 1 | Yes | 1 |  | 1 | 4 | 80 | Low |
| (Phumdoung et al., 2011) | No | 0 | Yes | 0 | No | 0 | Unknown | 0 | Yes | 1 | Yes | 1 | Did not report demographics for each group separately | 0 | 2 | 40 | Medium |
| (Pour-Edalati et al., 2019) | No | 0 | Yes | 0 | No | 0 | Unknown | 0 | Yes | 1 | Yes | 1 |  | 1 | 3 | 60 | Medium |
| (Rondung et al., 2018) | Yes | 1 | Yes | 0 | No | 0 | No | 0 | Yes | 1 | Yes | 1 |  | 1 | 4 | 80 | Low |
| (T. Saisto, Salmela-Aro, & Nurmi, 2001) | Yes | 1 | Yes | 0 | No | 0 | Unknown | 0 | Yes | 1 | No – FOC score not reported | 0 |  | 1 | 3 | 60 | Medium |
| (Terhi Saisto et al., 2006) | No | 0 | Yes | 0 | No | 0 | Unknown | 0 | Yes | 1 | No - FOC score not reported? | 0 |  | 1 | 2 | 40 | Medium |
| (Serçekuş & Başkale, 2016) | No | 0 | Yes | 0 | No | 0 | Unknown | 0 | Yes | 1 | Yes | 1 |  | 1 | 3 | 60 | Medium |
| (Sezen & Ünsalver, 2019) | No | 0 | Yes | 0 | No | 1 | Unknown | 1 | No | 0 | Yes | 1 | There is a significant difference in age between groups | 0 | 2 | 40 | Medium |
| (Sjogren, 1998) | No | 0 | Yes | 0 | No | 0 | Unknown | 0 | Yes | 1 | Yes | 1 | Did not report demographics for each group separately | 0 | 2 | 40 | Medium |
| (Soltani et al., 2017) | Yes | 1 | Yes | 0 | No | 0 | Unknown | 0 | No | 0 | Yes | 1 |  | 1 | 3 | 60 | Medium |
| (Sydsjö et al., 2012) | No | 0 | Yes | 0 | No | 0 | Unknown | 0 | Yes | 1 | Yes | 1 |  | 1 | 3 | 60 | Medium |
| (Sydsjo et al., 2014) | No | 0 | Yes | 0 | No | 0 | Unknown | 0 | Yes | 1 | No - FOC score not reported | 0 |  | 1 | 2 | 40 | Medium |
| (Sydsjö et al., 2015) | No | 0 | Yes | 0 | No | 0 | Unknown | 0 | Yes | 1 | Yes | 1 |  | 1 | 3 | 60 | Medium |
| (Taheri et al., 2014) | Yes | 1 | Yes | 0 | No | 0 | Unknown | 0 | Yes | 1 | Yes | 1 |  | 1 | 4 | 80 | Low |
| (Uçar & Golbasi, 2019) | No | 0 | Yes | 0 | No | 0 | Unknown | 0 | Yes | 1 | Yes | 1 |  | 1 | 3 | 60 | Medium |
| (Wahlbeck et al., 2018) | Yes | 1 | Yes | 0 | No | 0 | Unknown | 0 | Yes | 1 | Yes | 1 |  | 1 | 4 | 80 | Low |

Adapted questions from Cochrance risk of bias tool (Higgins et al., 2011)

Q1. Were participants randomly allocated?

Q2. Were participants aware of their allocation?

Q3. Were participants and personnel providing the intervention blind?

Q4. Were people performing analyses blind?

Q5. Were attritions and exclusions reported?

Q6. Were all outcomes reported?

Q7. Were there any other sources of bias?

**Table S4****. Description of studies in meta-analysis**

| Attribute | | n | % |
| --- | --- | --- | --- |
| Risk of bias | |  |  |
|  | Low | 10 | 36 |
|  | Medium | 18 | 64 |
| Country | |  |  |
|  | Sweden | 5 | 18 |
|  | Iran | 7 | 25 |
|  | Netherlands | 1 | 4 |
|  | Australia | 3 | 11 |
|  | Egypt | 1 | 4 |
|  | Finland | 3 | 11 |
|  | Turkey | 6 | 21 |
|  | Cyprus | 1 | 4 |
|  | Poland | 1 | 4 |
| RCT | |  |  |
|  | Yes | 12 | 43 |
|  | No | 15 | 54 |
|  | Partly | 1 | 4 |
| FOC pre-test | |  |  |
|  | Yes | 18 | 100 |
| Sample | |  |  |
|  | Women with FOC | 14 | 50 |
|  | All women | 14 | 50 |
| Proportion of nulliparous women | |  |  |
|  | 100% | 16 | 57 |
|  | 51-75% | 6 | 21 |
|  | 25-50% | 4 | 14 |
|  | Not Reported | 2 | 7 |
| Type of intervention | |  |  |
|  | Internet CBT | 2 | 7 |
|  | Counselling/psychotherapy | 7 | 25 |
|  | Antenatal education | 10 | 36 |
|  | Midwife-led counselling (known midwife) | 2 | 7 |
|  | Exercise (e.g. pilates, yoga) | 2 | 7 |
|  | Face to face CBT | 3 | 11 |
|  | Mindfulness | 1 | 4 |
|  | Hypnobirthing | 1 | 4 |
| Intensity of intervention | |  |  |
|  | High 6 or more sessions | 8 | 38 |
|  | Medium 3-5 sessions | 10 | 48 |
|  | Low 2 or less sessions | 3 | 14 |
|  | Not reported | 6 |  |
| Type of control | |  |  |
|  | Routine care | 22 | 79 |
|  | Counselling | 4 | 14 |
|  | Midwife-led counselling | 2 | 8 |
| Outcome measured | | | |
|  | FOC | 17 | 61 |
|  | FOC and birth outcome | 6 | 21 |
|  | Birth outcome | 5 | 18 |
| FOC measure used | | | |
|  | WDEQ-A | 16 | 70 |
|  | CAQ | 4 | 17 |
|  | FOBS | 1 | 4 |
|  | Own measure | 2 | 9 |
| Year of study | |  |  |
|  | Median: 2017  Range: 2006 - 2019 |  |  |

**Supplementary Information References**

Ahmadi, L., Karami, S., Faghihzadeh, S., Jafari, E., Oskoei, A., & Kharaghani, R. (2018). Effect of Couples Counseling Based on the Problem-Solving Approach on the Fear of Delivery, Self-Efficacy, and Choice of Delivery Mode in the Primigravid Women Requesting Elective Cesarean Section. *Preventative Care in Nursing & Midwifery Journal*, *7*(4), 32–40.

Airo (Toivanen), R., Korja, R., Saisto, T., Rouhe, H., Muotka, J., & Salmela–Aro, K. (2018). Changes in emotions and personal goals in primiparous pregnant women during group intervention for fear of childbirth. *Journal of Reproductive and Infant Psychology*. https://doi.org/10.1080/02646838.2018.1462477

American Psychiatric Association. (2000). *Diagnostic and Statistical Manual of Mental Disorders, Fourth Edition, Text Revision (DSM-IV-TR)*. https://doi.org/10.1176/appi.books.9780890423349

Andaroon, N., Kordi, M., Kimiaei, S., & Esmaeily, H. (2017). The effect of individual counseling program by a midwife on fear of childbirth in primiparous women. *Journal of Education and Health Promotion*. https://doi.org/10.4103/jehp.jehp_172_16

Areskog, B., Kjessler, R., & Uddenberg, N. (1982). Identification of women with significant fear of childbirth during late pregnancy. *Gynecologic and Obstetric Investigation*. https://doi.org/10.1159/000299490

Baleghi, M., Akerdi, E. M., & Pasha, Y. Z. (2016). The effect of relaxation on childbirth and an increase in natural childbirth. *Journal of Babol University of Medical Sciences*.

Baylis, R., Ekdahl, J., Haines, H., & Rubertsson, C. (2019). Women’s experiences of internet-delivered Cognitive Behaviour Therapy (iCBT) for Fear of Birth. *Women and Birth*. https://doi.org/10.1016/j.wombi.2019.05.006

Beck, A. T., & Steer, R. A. (1990). Manual for the Beck Anxiety Inventory. *Behaviour Research and Therapy*.

Beck, A. T., Steer, R. A., & Brown, G. K. (1996). Manual for the Beck depression inventory-II. *San Antonio, TX: Psychological Corporation*.

Bergström, M., Rudman, A., Waldenström, U., & Kieler, H. (2013). Fear of childbirth in expectant fathers, subsequent childbirth experience and impact of antenatal education: Subanalysis of results from a randomized controlled trial. *Acta Obstetricia et Gynecologica Scandinavica*. https://doi.org/10.1111/aogs.12147

Bulez, A., Ceber Turfan, E., & Sogukpinar, N. (2019). Evaluation of the effect of hypnobirthing education during antenatal period on fear of childbirth. *The European Research Journal*, *5*(2), 350–354.

Buysse, D. J., Reynolds, C. F., Monk, T. H., Berman, S. R., & Kupfer, D. J. (1989). The Pittsburgh sleep quality index: A new instrument for psychiatric practice and research. *Psychiatry Research*. https://doi.org/10.1016/0165-1781(89)90047-4

Byrne, J., Hauck, Y., Fisher, C., Bayes, S., & Schutze, R. (2014). Effectiveness of a mindfulness-based childbirth education pilot study on maternal self-efficacy and fear of childbirth. *Journal of Midwifery and Women’s Health*. https://doi.org/10.1111/jmwh.12075

Cox, J. L., Holden, J. M., & Sagovsky, R. (1987). Detection of Postnatal Depression: Development of the 10-item Edinburgh Postnatal Depression scale. *British Journal of Psychiatry*. https://doi.org/10.1192/bjp.150.6.782

Crawford, J. R., & Henry, J. D. (2004). The Positive and Negative Affect Schedule (PANAS): Construct validity, measurement properties and normative data in a large non-clinical sample. *British Journal of Clinical Psychology*. https://doi.org/10.1348/0144665031752934

Diener, E., Emmons, R. A., Larsem, R. J., & Griffin, S. (1985). The Satisfaction With Life Scale. *Journal of Personality Assessment*. https://doi.org/10.1207/s15327752jpa4901_13

El-Malky, E., El-Homosy, S., Ashour, E., & Shehada, Y. (2018). Effectiveness of Antenatal Nursing Intervention on Childbirth’s Fears, Psychological - Wellbeing and Pregnancy Outcomes in Primipara’s Women. *Journal of Nursing Science*, *4*(2), 17–24.

Fenwick, J., Toohill, J., Gamble, J., Creedy, D. K., Buist, A., Turkstra, E., … Ryding, E. L. (2015). Effects of a midwife psycho-education intervention to reduce childbirth fear on women’s birth outcomes and postpartum psychological wellbeing. *BMC Pregnancy and Childbirth*. https://doi.org/10.1186/s12884-015-0721-y

Fisher, C., Hauck, Y., Bayes, S., & Byrne, J. (2012). Participant experiences of mindfulness-based childbirth education: A qualitative study. *BMC Pregnancy and Childbirth*. https://doi.org/10.1186/1471-2393-12-126

Gökçe İsbir, G., İnci, F., Önal, H., & Yıldız, P. D. (2016). The effects of antenatal education on fear of childbirth, maternal self-efficacy and post-traumatic stress disorder (PTSD) symptoms following childbirth: an experimental study. *Applied Nursing Research*. https://doi.org/10.1016/j.apnr.2016.07.013

Guder, D., Yalvac, S., & Vural, G. (2018). The effect of pregnancy Pilates-assisted childbirth preparation training on childbirth fear and neonatal outcomes: a quasi-experimental/quantitative research. *Quality and Quantity*, *52*, 2667–2679.

Guszkowska, M. (2014). The effect of exercise and childbirth classes on fear of childbirth and locus of labor pain control. *Anxiety, Stress and Coping*. https://doi.org/10.1080/10615806.2013.830107

Haapio, S., Kaunonen, M., Arffman, M., & Åstedt-Kurki, P. (2017). Effects of extended childbirth education by midwives on the childbirth fear of first-time mothers: an RCT. *Scandinavian Journal of Caring Sciences*. https://doi.org/10.1111/scs.12346

Halvorsen, L., Nerum, H., Sørlie, T., & Øian, P. (2010). Does counsellor’s attitude influence change in a request for a caesarean in women with fear of birth? *Midwifery*. https://doi.org/10.1016/j.midw.2008.04.011

Henriksen, L., Borgen, A., Risløkken, J., & Lukasse, M. (2018). Fear of birth: Prevalence, counselling and method of birth at five obstetrical units in Norway. *Women and Birth*. https://doi.org/10.1016/j.wombi.2018.11.008

Higgins, J. P. T., Altman, D. G., Gøtzsche, P. C., Jüni, P., Moher, D., Oxman, A. D., … Sterne, J. A. C. (2011). The Cochrane Collaboration’s tool for assessing risk of bias in randomised trials. *BMJ (Online)*. https://doi.org/10.1136/bmj.d5928

Hildingsson, I., Karlström, A., Rubertsson, C., & Haines, H. (2019). Women with fear of childbirth might benefit from having a known midwife during labour. *Women and Birth*. https://doi.org/10.1016/j.wombi.2018.04.014

Hildingsson, I., & Rubertsson, C. (2019). Childbirth experiences among women with fear of birth randomized to internet-based cognitive therapy or midwife counseling. *Journal of Psychosomatic Obstetrics & Gynecology*, 1–10. https://doi.org/10.1080/0167482X.2019.1634047

Hildingsson, I., Rubertsson, C., Karlström, A., & Haines, H. (2018). Caseload midwifery for women with fear of birth is a feasible option. *Sexual and Reproductive Healthcare*. https://doi.org/10.1016/j.srhc.2018.02.006

Hildingsson, I., Rubertsson, C., Karlström, A., & Haines, H. (2019). A known midwife can make a difference for women with fear of childbirth- birth outcome and women’s experiences of intrapartum care. *Sexual and Reproductive Healthcare*. https://doi.org/10.1016/j.srhc.2019.06.004

Horowitz, M., Wilner, N., & Alvarez, W. (1979). Impact of event scale: A measure of subjective stress. *Psychosomatic Medicine*. https://doi.org/10.1097/00006842-197905000-00004

Hunter, L., Bormann, J., Belding, W., Sobo, E. J., Axman, L., Reseter, B. K., … Miranda Anderson, V. (2011). Satisfaction and use of a spiritually based mantram intervention for childbirth-related fears in couples. *Applied Nursing Research*. https://doi.org/10.1016/j.apnr.2009.06.002

Ip, W. Y., Tang, C. S. K., & Goggins, W. B. (2009). An educational intervention to improve women’s ability to cope with childbirth. *Journal of Clinical Nursing*. https://doi.org/10.1111/j.1365-2702.2008.02720.x

Irmak Vural, P., & Aslan, E. (2019). Emotional freedom techniques and breathing awareness to reduce childbirth fear: A randomized controlled study. *Complementary Therapies in Clinical Practice*. https://doi.org/10.1016/j.ctcp.2019.02.011

Karabulut, O., Coşkuner Potur, D., Doğan Merih, Y., Cebeci Mutlu, S., & Demirci, N. (2016). Does antenatal education reduce fear of childbirth? *International Nursing Review*. https://doi.org/10.1111/inr.12223

Khedr, N., & Eldeen, M. (2017). Effect of Healthy Instructions on Reducing Pregnant Women’s Fear of Normal Delivery and Preferences for Cesarean Delivery. *American Journal of Nursing Science*, *6*(3), 176–184. https://doi.org/0.11648/j.ajns.20170603.15

Khorsandi, M., Ghofranipoor, F., Heidarneia, A., Fagheihzade, S., Akbarzadeh, A., & Vafaei, M. (2008). Efficacy of delivery in pregnant women. *Medical Journal of The Islamic Republic of Iran (MJIRI)*, *26*(89–95).

Kizilirmak, A., & Başer, M. (2016). The effect of education given to primigravida women on fear of childbirth. *Applied Nursing Research*. https://doi.org/10.1016/j.apnr.2015.04.002

Klabbers, G. A., Paarlberg, K. M., & Vingerhoets, J. J. M. (2018). Does haptotherapy benefit mother-child bonding in women with high fear of childbirth? *International Journal of Haptonomy and Haptotherapy*, *3*(1), 1–7.

Klabbers, G. A., Wijma, K., Paarlberg, K. M., Emons, W. H. M., & Vingerhoets, A. J. J. M. (2019). Haptotherapy as a new intervention for treating fear of childbirth: a randomized controlled trial. *Journal of Psychosomatic Obstetrics and Gynecology*. https://doi.org/10.1080/0167482X.2017.1398230

Kordi, M., Bakhshi, M., Masoudi, S., & Esmaily, H. (2017). Effect of a childbirth psychoeducation program on the level of fear of childbirth in primigravid women. *Evidence Based Care Journal*. https://doi.org/10.22038/EBCJ.2017.25676.1575

Kulkarni, A., Wright, E., & Kingdom, J. (2014). Web-Based Education and Attitude to Delivery by Caesarean Section in Nulliparous Women. *Journal of Obstetrics and Gynaecology Canada*. https://doi.org/10.1016/S1701-2163(15)30478-3

Larsson, B., Hildingsson, I., Ternström, E., Rubertsson, C., & Karlström, A. (2019). Women’s experience of midwife-led counselling and its influence on childbirth fear: A qualitative study. *Women and Birth*. https://doi.org/10.1016/j.wombi.2018.04.008

Larsson, B., Karlström, A., Rubertsson, C., & Hildingsson, I. (2015). The effects of counseling on fear of childbirth. *Acta Obstetricia et Gynecologica Scandinavica*. https://doi.org/10.1111/aogs.12634

Larsson, B., Karlström, A., Rubertsson, C., Ternström, E., Ekdahl, J., Segebladh, B., & Hildingsson, I. (2017). Birth preference in women undergoing treatment for childbirth fear: A randomised controlled trial. *Women and Birth*. https://doi.org/10.1016/j.wombi.2017.04.004

Lowe, N. K. (2000). Self-efficacy for labor and childbirth fears in nulliparous pregnant women. *Journal of Psychosomatic Obstetrics and Gynecology*. https://doi.org/10.3109/01674820009085591

Lyberg, A., & Severinsson, E. (2010a). Fear of childbirth: Mothers’ experiences of team-midwifery care - a follow-up study. *Journal of Nursing Management*. https://doi.org/10.1111/j.1365-2834.2010.01103.x

Lyberg, A., & Severinsson, E. (2010b). Midwives’ supervisory styles and leadership role as experienced by Norwegian mothers in the context of a fear of childbirth. *Journal of Nursing Management*. https://doi.org/10.1111/j.1365-2834.2010.01083.x

Masoumi, S. Z., Kazemi, F., Oshvandi, K., Jalali, M., Esmaeili-Vardanjani, A., & Rafiei, H. (2016). Effect of Training Preparation for Childbirth on Fear of Normal Vaginal Delivery and Choosing the Type of Delivery Among Pregnant Women in Hamadan, Iran: A Randomized Controlled Trial. *Journal of Family & Reproductive Health*.

Melender, H. L. (2002). Experiences of fears associated with pregnancy and childbirth: A study of 329 pregnant women. *Birth*. https://doi.org/10.1046/j.1523-536X.2002.00170.x

Moher, D., Shamseer, L., Clarke, M., Ghersi, D., Liberati, A., Petticrew, M., … Whitlock, E. (2016). Preferred reporting items for systematic review and meta-analysis protocols (PRISMA-P) 2015 statement. *Revista Espanola de Nutricion Humana y Dietetica*. https://doi.org/10.1186/2046-4053-4-1

Müller, M. E. (1994). A questionnaire to measure mother-to-infant attachment. *Journal of Nursing Measurement*.

Narita, Y., Shinohara, H., & Kodama, H. (2018). Resting Heart Rate Variability and the Effects of Biofeedback Intervention in Women with Low-Risk Pregnancy and Prenatal Childbirth Fear. *Applied Psychophysiology Biofeedback*. https://doi.org/10.1007/s10484-018-9389-1

Navaee, M., & Abedian. (2015). Effect of role play education on primiparous women’s fear of natural delivery and their decision on the mode of delivery. *Iranian Journal of Nursing and Midwifery Research*, *20*(1), 41–46.

Nerum, H., Halvorsen, L., Sørlie, T., & Øian, P. (2006). Maternal request for cesarean section due to fear of birth: Can it be changed through crisis-oriented counseling? *Birth*. https://doi.org/10.1111/j.1523-536X.2006.00107.x

Nieminen, K., Malmquist, A., Wijma, B., Ryding, E. L., Andersson, G., & Wijma, K. (2015). Nulliparous pregnant women’s narratives of imminent childbirth before and after internet-based cognitive behavioural therapy for severe fear of childbirth: A qualitative study. *BJOG: An International Journal of Obstetrics and Gynaecology*. https://doi.org/10.1111/1471-0528.13358

Nieminen, Katri, Andersson, G., Wijma, B., Ryding, E. L., & Wijma, K. (2016). Treatment of nulliparous women with severe fear of childbirth via the Internet: A feasibility study. *Journal of Psychosomatic Obstetrics and Gynecology*. https://doi.org/10.3109/0167482X.2016.1140143

Nurmi, J. E. (1991). How do adolescents see their future? A review of the development of future orientation and planning. *Developmental Review*. https://doi.org/10.1016/0273-2297(91)90002-6

Ozdemir, M. E., Cilingir, I. U., Ilhan, G., Yildiz, E., & Ohanoglu, K. (2018). The effect of the systematic birth preparation program on fear of vaginal delivery and quality of life. *Archives of Gynecology and Obstetrics*. https://doi.org/10.1007/s00404-018-4835-0

Phumdoung, S., Youngvanichsate, S., & Wongmuneeworn, W. (2011). The Effects of Instrumental Marching and Cheerful Music on Women’s Sense of Power, Self-Control, Fear of Childbirth, and Second-Stage Duration During Their Second Stage of Labor. *Songklanagarind Medical Journal*, *29*(4).

Pour-Edalati, M., Moghadam, N., Shahesmaeili, A., & Salehi-Nejad, P. (2019). Exploring the Effect of Mindfulness-Based Stress Reduction on Childbirth Fear Among Single-Child Mothers in the City of Kerman, Iran (2017): A Clinical Trial Study. *Medical Surgery Nursing Journal*, *7*(4), e90247.

Rabin, R., Gudex, C., Selai, C., & Herdman, M. (2014). From translation to version management: A history and review of methods for the cultural adaptation of the euroqol five-dimensional questionnaire. *Value in Health*. https://doi.org/10.1016/j.jval.2013.10.006

Rondung, E., Ternström, E., Hildingsson, I., Haines, H. M., Sundin, Ö., Ekdahl, J., … Rubertsson, C. (2018). Comparing internet-based cognitive behavioral therapy with standard care for women with fear of birth: Randomized controlled trial. *Journal of Medical Internet Research*. https://doi.org/10.2196/10420

Rouhe, H., Salmela-Aro, K., Toivanen, R., Tokola, M., Halmesmäki, E., & Saisto, T. (2013). Obstetric outcome after intervention for severe fear of childbirth in nulliparous women - Randomised trial. *BJOG: An International Journal of Obstetrics and Gynaecology*. https://doi.org/10.1111/1471-0528.12011

Rouhe, Hanna, Salmela-Aro, K., Toivanen, R., Tokola, M., Halmesmäki, E., Ryding, E. L., & Saisto, T. (2015). Group psychoeducation with relaxation for severe fear of childbirth improves maternal adjustment and childbirth experience-a randomised controlled trial. *Journal of Psychosomatic Obstetrics and Gynecology*. https://doi.org/10.3109/0167482X.2014.980722

Rouhe, Hanna, Salmela-Aro, K., Toivanen, R., Tokola, M., Halmesmäki, E., & Saisto, T. (2015). Life satisfaction, general well-being and costs of treatment for severe fear of childbirth in nulliparous women by psychoeducative group or conventional care attendance. *Acta Obstetricia et Gynecologica Scandinavica*. https://doi.org/10.1111/aogs.12594

Ryding, E. L., Persson, A., Onell, C., & Kvist, L. (2003). An evaluation of midwives’ counseling of pregnant women in fear of childbirth. *Acta Obstetricia et Gynecologica Scandinavica*. https://doi.org/10.1034/j.1600-0412.2003.820102.x

Ryding, E. L., Read, S., Rouhe, H., Halmesmäki, E., Salmela-Aro, K., Toivanen, R., … Saisto, T. (2018). Partners of nulliparous women with severe fear of childbirth: A longitudinal study of psychological well-being. *Birth*. https://doi.org/10.1111/birt.12309

Saisto, T., Salmela-Aro, K., & Nurmi, J. E. (2001). A randomized controlled trial of intervention in fear of childbirth. *Obstetrics and Gynecology*. https://doi.org/10.1016/S0029-7844(01)01552-6

Saisto, T., Salmela-Aro, K., Nurmi, J. E., & Halmesm??ki, E. (2001). Psychosocial predictors of disappointment with delivery and puerperal depression: A longitudinal study. *Acta Obstetricia et Gynecologica Scandinavica*. https://doi.org/10.1034/j.1600-0412.2001.800108.x

Saisto, Terhi, Toivanen, R., Salmela-Aro, K., & Halmesmäki, E. (2006). Therapeutic group psychoeducation and relaxation in treating fear of childbirth. *Acta Obstetricia et Gynecologica Scandinavica*. https://doi.org/10.1080/00016340600756920

Salmela-Aro, K., Read, S., Rouhe, H., Halmesmäki, E., Toivanen, R. M., Tokola, M. I., & Saisto, T. (2012). Promoting positive motherhood among nulliparous pregnant women with an intense fear of childbirth: RCT intervention. *Journal of Health Psychology*. https://doi.org/10.1177/1359105311421050

Serçekuş, P., & Başkale, H. (2016). Effects of antenatal education on fear of childbirth, maternal self-efficacy and parental attachment. *Midwifery*. https://doi.org/10.1016/j.midw.2015.11.016

Sezen, C., & Ünsalver, B. Ö. (2019). Group art therapy for the management of fear of childbirth. *Arts in Psychotherapy*. https://doi.org/10.1016/j.aip.2018.11.007

Sjogren, B. (1998). Fear of childbirth and psychosomatic support - a follow up of 72 women. *Acta Obstetricia Et Gynecologica Scandinavica*, *77*, 819–825.

Soltani, F., Eskandari, Z., Khodakarami, B., Parsa, P., & Roshanaei, G. (2017). The effect of self-efficacy oriented counselling on controlling the fear of natural delivery in primigravida women. *Journal of Pharmaceutical Sciences and Research*.

Stroup, D. F., Berlin, J. A., Morton, S. C., Olkin, I., Williamson, G. D., Rennie, D., … Thacker, S. B. (2000). Meta-analysis of observational studies in epidemiology: A proposal for reporting. *Journal of the American Medical Association*. https://doi.org/10.1001/jama.283.15.2008

Sydsjo, G., Bladh, M., Lilliecreutz, C., Persson, A. M., Vyoni, H., & Josefsson, A. (2014). Obstetric outcomes for nulliparous women who received routine individualized treatment for severe fear of childbirth - a retrospective case control study. *BMC Pregnancy and Childbirth*. https://doi.org/10.1186/1471-2393-14-126

Sydsjö, G., Blomberg, M., Palmquist, S., Angerbjörn, L., Bladh, M., & Josefsson, A. (2015). Effects of continuous midwifery labour support for women with severe fear of childbirth. *BMC Pregnancy and Childbirth*. https://doi.org/10.1186/s12884-015-0548-6

Sydsjö, G., Sydsjö, A., Gunnervik, C., Bladh, M., & Josefsson, A. (2012). Obstetric outcome for women who received individualized treatment for fear of childbirth during pregnancy. *Acta Obstetricia et Gynecologica Scandinavica*. https://doi.org/10.1111/j.1600-0412.2011.01242.x

Taheri, Z., Mazaheri, M. A., Khorsandi, M., Hassanzadeh, A., & Amiri, M. (2014). Effect of educational intervention on self-efficacy for choosing delivery method among pregnant women in 2013. *International Journal of Preventive Medicine*.

Ternström, E., Hildingsson, I., Haines, H., & Rubertsson, C. (2016). Pregnant women’s thoughts when assessing fear of birth on the Fear of Birth Scale. *Women and Birth*. https://doi.org/10.1016/j.wombi.2015.11.009

Toohill, J., Callander, E., Gamble, J., Creedy, D. K., & Fenwick, J. (2017). A cost effectiveness analysis of midwife psycho-education for fearful pregnant women - a health system perspective for the antenatal period. *BMC Pregnancy and Childbirth*. https://doi.org/10.1186/s12884-017-1404-7

Toohill, Jocelyn, Fenwick, J., Gamble, J., Creedy, D. K., Buist, A., Turkstra, E., & Ryding, E. L. (2014). A Randomized Controlled Trial of a Psycho-Education Intervention by Midwives in Reducing Childbirth Fear in Pregnant Women. *Birth*. https://doi.org/10.1111/birt.12136

Turkstra, E., Mihala, G., Scuffham, P. A., Creedy, D. K., Gamble, J., Toohill, J., & Fenwick, J. (2017). An economic evaluation alongside a randomised controlled trial on psycho-education counselling intervention offered by midwives to address women’s fear of childbirth in Australia. *Sexual and Reproductive Healthcare*. https://doi.org/10.1016/j.srhc.2016.08.003

Uçar, T., & Golbasi, Z. (2019). Effect of an educational program based on cognitive behavioral techniques on fear of childbirth and the birth process. *Journal of Psychosomatic Obstetrics and Gynecology*. https://doi.org/10.1080/0167482X.2018.1453800

van Bakel, H. J. A., Maas, A. J. B. M., Vreeswijk, C. M. J. M., & Vingerhoets, A. J. J. M. (2013). Pictorial representation of attachment: Measuring the parent-fetus relationship in expectant mothers and fathers. *BMC Pregnancy and Childbirth*. https://doi.org/10.1186/1471-2393-13-138

Wahlbeck, H., Kvist, L. J., & Landgren, K. (2018). Gaining hope and self-confidence—An interview study of women’s experience of treatment by art therapy for severe fear of childbirth. *Women and Birth*. https://doi.org/10.1016/j.wombi.2017.10.008

Wijma, K., Soderquist, J., Carlsson, I., & Wijma, B. (2000). Prevalence of posttraumatic stress disorder among gynaecological patients with a history of sexual and physical abuse. *Journal of Interpersonal Violence*, *15*, 944–958.

Wijma, K., Wijma, B., & Zar, M. (1998). Psychometric aspects of the W-DEQ; A new questionnaire for the measurement of fear of childbirth. *Journal of Psychosomatic Obstetrics and Gynaecology*. https://doi.org/10.3109/01674829809048501

World Health Organization. (1996). *WHOQOL-BREF Introduction, administration, scoring and generic version of the assessment, programme on mental health.* Geneva: World Health Organization.
